# Macrophage Zc3h12c Limits Tissue Inflammation and Injury via Alternative Splicing of Pre‐mRNA

**DOI:** 10.1002/advs.202506707

**Published:** 2025-08-20

**Authors:** Chenyu Li, Julian Aurelio Marschner, Yoshihiro Kusunoki, Ningxin Zhang, Xiaoxin Li, Hao Deng, Zhibo Zhao, Kanako Watanabe‐Kusunoki, Zhihui Zhu, Yan Xu, Stefanie Steiger, Maciej Lech, Katalin Susztak, Christian Schulz, Hans‐Joachim Anders

**Affiliations:** ^1^ Division of Nephrology Department of Medicine IV Hospital of the Ludwig‐Maximilians University 80336 Munich Germany; ^2^ Department of Nephrology The Affiliated Hospital of Qingdao University Qingdao 266005 China; ^3^ Department of Pharmacy Ludwig‐Maximilians‐Universität München 81377 Munich Germany; ^4^ Department of Rheumatology Endocrinology and Nephrology Faculty of Medicine and Graduate School of Medicine Hokkaido University Sapporo 060‐8638 Japan; ^5^ Department of Nephrology Shanxi Provincial People's Hospital The Fifth Clinical Medical College of Shanxi Medical University Taiyuan Shanxi 030012 China; ^6^ Renal Electrolyte and Hypertension Division Department of Medicine University of Pennsylvania Perelman School of Medicine Philadelphia 19104 USA; ^7^ Institute for Diabetes Obesity and Metabolism University of Pennsylvania Perelman School of Medicine Philadelphia 19104 USA; ^8^ Department of Genetics University of Pennsylvania Perelman School of Medicine Philadelphia 19104 USA; ^9^ Department of Cardiology Klinikum der Universität München Ludwig‐Maximilians‐Universität 81377 Munich Germany; ^10^ DZHK (German Centre for Cardiovascular Research) partner site Munich Heart Alliance 80636 Munich Germany; ^11^ Department of Immunopharmacology Mannheim Institute for Innate Immunoscience (MI3) Medical Faculty Mannheim Heidelberg University 68167 Mannheim Germany

**Keywords:** alternative splicing, innate immunity, kidney injury, macrophages, RNA binding protein

## Abstract

RNA‐binding proteins regulate post‐transcriptional gene translation, but the macrophage‐specific role of Zc3h12c remains poorly characterized. Here, the role of Zc3h12c in macrophages is characterized using Tnfrsf11a^Cre^‐Zc3h12c^flox/flox^ mice. Both Tnfrsf11a and Zc3h12c are highly expressed in the kidney tissue from patients with chronic kidney disease and showed a positive association with an interstitial fibrosis score. Single cell RNA sequencing demonstrated abundant Tnfrsf11a expression in murine kidney macrophages and a correlation with the induction of chemokines, macrophage phagocytosis, and activation upon kidney injury. In various kidney injury models, Tnfrsf11a^Cre^‐Zc3h12c^flox/flox^ mice suffered from more injury and inflammation in the kidney, characterized by an increase in Ccr2 positive leukocyte infiltration. Mechanistic in vitro studies revealed that Zc3h12c suppresses macrophage activation toward a pro‐inflammatory phenotype, modulates macrophage survival, migration, and phagocytosis. Both in silico and in vitro analysis indicated that Zc3h12c regulates the pro‐inflammatory cytokines/chemokines and chemokine receptors expression and modulates the alternative splicing of pre‐mRNAs STAT1. Thus, macrophage‐derived Zc3h12c limits tissue inflammation and injury, potentially via alternate splicing of pre‐mRNAs.

## Graphical abstract

This work defines the context‐dependent function of Zc3h12c in macrophages using the Tnfrsf11a‐Cre system. It reveals Zc3h12c's dual mechanism—regulating RNA degradation and alternative splicing—with STAT1 as a key target. The findings elucidate a novel post‐transcriptional pathway and identify STAT1 isoforms as potential therapeutic targets for kidney injury.

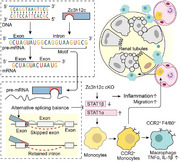

## 1 Introduction

Macrophages (Mφ) are a group of highly plastic cells that can polarize into different functional phenotypes based on the signals they receive from their microenvironment.^[^ ^1^ ^]^ It is their plasticity in activation that involves them in diverse physiological and pathological processes, including all phases of tissue injury and repair.^[^ ^2^ ^]^ For example, during the early stage of acute kidney injury (AKI), exposure to damage‐associated molecular patterns induces a pro‐inflammatory phenotype in both kidney‐resident and monocyte‐derived infiltrating Mφ in the tubulointerstitial compartment. These pro‐inflammatory Mφ are a source of cytokines and chemokines, leading to the recruitment of more immune cells in a positive auto‐amplification loop promoting acute tubular necrosis, i.e., necroinflammation. Subsequently, they can switch their phenotype to support the resolution of inflammation, tubule repair, and adaptation through the secretion of factors such as transforming growth factor beta, Interleukin‐22, and fibronectin.^[^ ^3^, ^4^, ^5^, ^6^ ^]^ A dynamic balance guarantees inflammatory outcomes in different regions of the injured organ as well as across different phases of the injury and healing process, with trade‐offs on both sides. For example, over‐activation of anti‐inflammatory Mφ increases the production of transforming growth factor beta and galectin‐3, which drives irreversible interstitial fibrosis of the kidney, i.e., chronic kidney disease (CKD).^[^ ^7^ ^]^ On the other hand, persistent Mφ‐driven inflammation leads to more parenchymal cell death and organ failure.

Inflammation phenotyping relies on transcriptomic processes, whereby the transcription of immune‐related genes and cytokines is subsequently followed by the translation of mRNA into protein.^[^ ^8^, ^9^ ^]^ However, RNA‐binding proteins provide another level of regulation, i.e., not all RNAs translate into proteins.^[^ ^10^ ^]^ The Regnase family of RNA‐binding proteins, including Regnase‐1—4 (also known as monocyte chemoattractant protein‐induced proteins, MCPIP1‐4), plays a pivotal role in modulating immune responses, particularly within Mφ.^[^ ^11^, ^12^, ^13^, ^14^ ^]^ These proteins are categorized as Cys‐Cys‐Cys‐His‐type (CCCH) Zinc Finger proteins; thus, they are also referred to as Zinc Finger CCCH Domain‐Containing Protein 12 A‐D (Zc3h12a‐d).^[^ ^15^ ^]^ All members from this family possess two conserved functional domains, a zinc finger domain which binding of RNA and the Pilt‐N‐terminus (PIN)‐like domain that enables RNA cleaving.^[^ ^15^, ^16^, ^17^, ^18^ ^]^ Zc3h12a and Zc3h12d are predominantly expressed in the spleen, lymph nodes, and thymus,^[^ ^12^, ^19^ ^]^ whereas Zc3h12b is exclusively expressed in the intestine^[^ ^12^ ^]^ and brain tissue.^[^ ^20^ ^]^ In contrast, Zc3h12c is hardly expressed in any of these organs but shows relatively high expression levels in the heart, lung, brain, liver, and kidney.^[^ ^12^ ^]^ The pronounced expression of Zc3h12c in extra‐lymphoid tissues, particularly within the kidney, may reflect its functional association with tissue‐resident and mature Mφ.^[^ ^21^, ^22^, ^23^ ^]^

The functional landscape of the Regnase family remains incompletely characterized, particularly for Zc3h12c. To date, only Zc3h12a has been well‐defined: its deficiency in mice develop anemia, hypergammaglobulinemia, autoantibodies, and tissue inflammation.^[^ ^11^ ^]^ This phenotype stems from its role as a critical negative regulator of Mφ activation.^[^ ^18^ ^]^ Zc3h12a achieves this by directly targeting mRNAs encoding pro‐inflammatory cytokines, including IL‐6, IL‐12p40, and IL‐1β, for degradation^[^ ^16^, ^24^ ^]^–cytokines also implicated in kidney Mφ function.^[^ ^25^ ^]^ Consequently, the absence of Zc3h12a in Mφ results in heightened production of these cytokines upon stimulation, underpinning the spontaneous autoinflammation observed in knockout models.^[^ ^26^, ^27^, ^28^, ^29^ ^]^ In contrast, Zc3h12c has been established to exert an anti‐inflammatory function, mediated through the regulation of TNF and IL‐6 production in Mφ and plasmacytoid dendritic cells.^[^ ^21^, ^22^ ^]^ However, significant controversy persists regarding its precise phenotype and mechanistic basis. The Zc3h12c‐deficiency did not affect skin inflammation in an imiquimod‐induced psoriasis model,^[^ ^30^ ^]^ but myeloid cell‐specific Zc3h12c‐deficiency did protect from psoriasis lesions.^[^ ^23^ ^]^ The reason for these discrepancies in phenotype may be due to differences in the targeted deletion sites of Zc3h12c and the broad range of targets for the LysM‐Cre approach.^[^ ^31^ ^]^ Collectively, these conflicting results suggest that Zc3h12c may exert distinct functions in different immune cell subsets, highlighting the need for more precise Cre–driver lines to dissect its cell type–specific roles.

Tnfrsf11a, as a receptor for a transmembrane protein from the TNF superfamily, plays a central role in osteoclastogenesis.^[^ ^32^, ^33^, ^34^ ^]^ It serves as a novel and precise marker that effectively identifies general tissue‐resident Mφ while not targeting other hematopoietic cells and their offspring.^[^ ^35^, ^36^ ^]^ Recent evidence showed that 20–30% of peripheral monocytes, 80% of Mφ,^[^ ^37^, ^38^ ^]^ and 100% of kidney‐resident Mφ expressed Tnfrsf11a.^[^ ^39^ ^]^ It is also co‐expressed with Mφ markers CD206 and CD80,^[^ ^37^ ^]^ and is associated with cell chemotaxis.^[^ ^35^ ^]^ Here, we used Tnfrsf11a‐Cre instead of Csf1r‐Cre^[^ ^40^ ^]^ or LysM‐Cre^[^ ^41^ ^]^ to explore the specific deletion of Zc3h12c in tissue injury and repair within the context of Tnfrsf11a^+^ Mφ. To this end, we generated Zc3h12c^flox/flox^; Tnfrsf11a‑Cre mice, allowing us to selectively delete Zc3h12c in Tnfrsf11a‑expressing cells and thereby interrogate its function in Mφ, particularly in the context of kidney injury and repair, a function that has yet to be elucidated. Based on the utilization of this innovative mouse strain, we hypothesized that Zc3h12c would act as a regulator of Mφ activation and therefore modulate early tissue necroinflammation as well as the subsequent healing phase, leading to either tissue reconstitution or persistent atrophy and scarring via its effects on pre‐mRNA.

## 2 Results

### 2.1 Tissue‐Resident and Infiltrating Tnfrsf11a^+^ Mφ Express Zc3h12c upon Acute Tissue Injury

**Figure 1:**
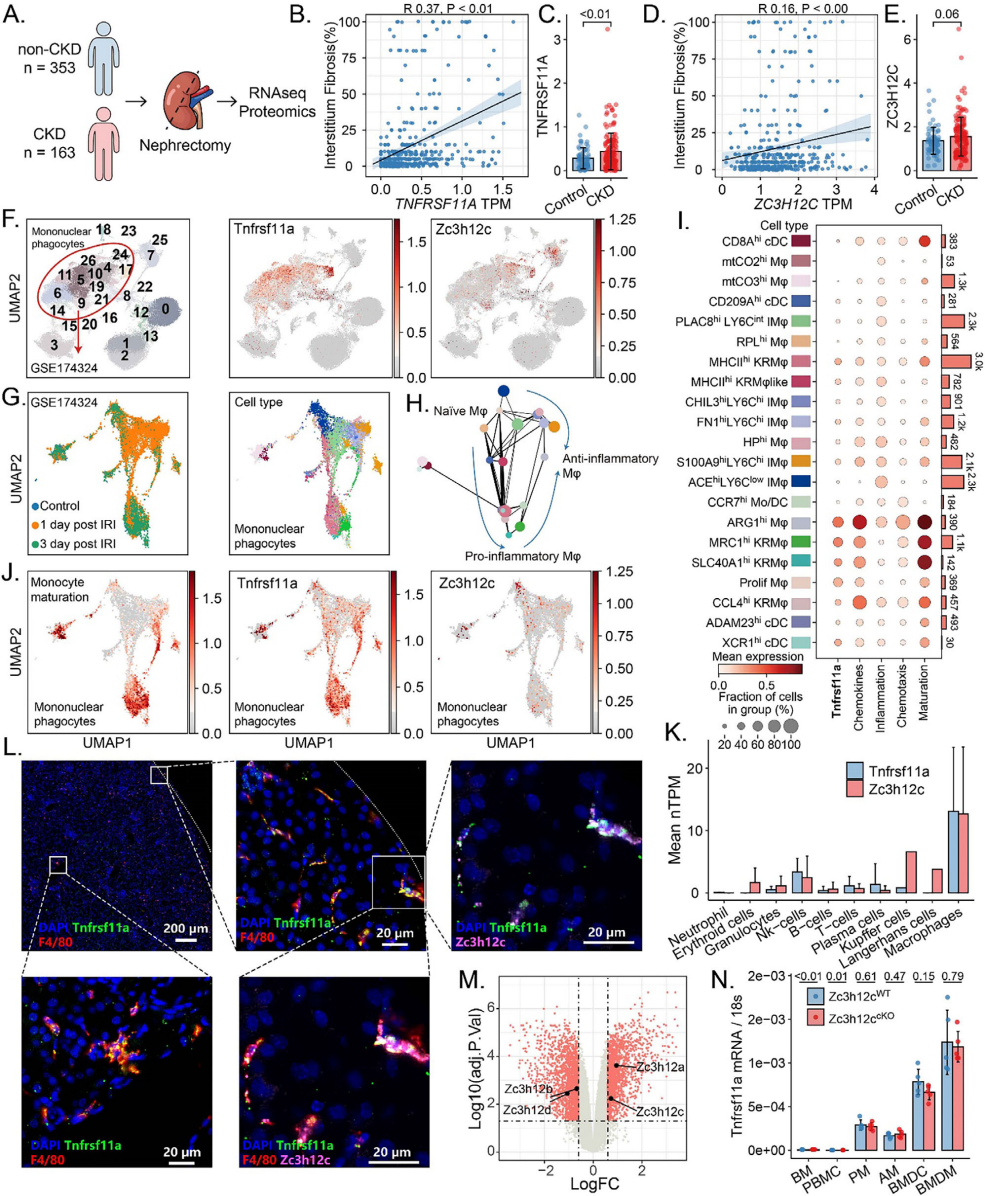
Zc3h12c and Tnfrsf11a are associated with monocytes maturation in the kidney. A) Schematic illustrating the study design of omics data from nephrectomy samples of non‐CKD and CKD patients. B–E) Scatter plots showing the correlation between TNFRSF11A (B) and ZC3H12C (D) transcript per million (TPM) values with interstitial fibrosis percentage in kidney samples, with corresponding Pearson correlation coefficient (R) and p‐values. Box plots for the expression levels of TNFRSF11A (C) and ZC3H12C (E) in non‐CKD and CKD kidney tissue. F) UMAP plot for sorted mononuclear phagocytic cells from blood (by CD11b^+^Ly6c^+^), kidney (CD11b^+^F4/80^+^), and spleen (CD11b^+^) of healthy and post‐ischemic mice,1‐and 3‐days after kidney ischemia‐reperfusion injury (IRI). The clusters were segmented by the Louvain algorithm with a resolution of 1. According to markers, the clusters in the red circle represent various subsets of mononuclear phagocytes. Zc3h12c and Tnfrsf11a are highly expressed in mononuclear phagocytes. G) Partition‐based graph abstraction of mononuclear phagocytes in blood and kidney after IRI, colored by the corresponding grouping and cell annotation based on the H) pseudo‐time trajectory analysis. I) Cell annotation and dot plot for the mononuclear phagocytes in blood and kidney after IRI. The dot size represents the proportion of cells in the group, and the colors represent mean expression. The bar plot on the right side of the box shows the numbers of cells in each group. Cells were annotated based on markers from ImmGen mouse immune cell datasets. J) The activation for phagocytosis, inflammation, chemotaxis, trafficking, and monocyte maturation was assessed based on the average expression of corresponding genes. K) Immunofluorescence staining for Tnfrsf11a (green), F4/80 (red), Zc3h12c (pink) in healthy mouse kidney. L) Human ZC3H12C and TNFRSF11A mRNA expression in various mononuclear phagocytes from the ProteinAtlas datasets. M) Expression of Zc3h12c mRNA in kidney Mφ after IRI (GSE52004). The vertical dashed lines indicate the cut‐off for a 1.5‐fold change (FC), while the horizontal dashed line marks the cutoff for the adjusted p‐value of 0.05. The mRNA levels for N) Tnfrsf11a in bone marrow (BM), peripheral blood mononuclear cell (PBMC) alveolar Mφ (AM), BM derived dendritic cells (BMDC), and BM derived Mφ (BMDM) from Tnfrsf11a‐Zc3h12c^cKO^ and wildtype (WT) mice. All quantitative data are means ± SD (n = 516). Student t‐test was employed for the statistical examination. IM, infiltrating monocytes; Mo, Monocyte; KRM, kidney resident Mφ; Mo, Mφ; NK, natural killer cell; Tnfrsf11a, TNF Receptor Superfamily Member 11a; Zc3h12c, Zinc Finger CCCH‐Type Containing 12C.

First, we characterized the expression of Tnfrsf11a and Zc3h12c in a large‐scale RNA‐seq and proteomics analysis of human kidney tissues obtained from nephrectomy (**Figure** **1**A). Both Tnfrsf11a and Zc3h12c mRNA were highly expressed in the CKD patient kidney samples and were positively associated with the interstitial fibrosis score (Figure 1B–E). However, Tnfrsf11a protein levels did not correlate significantly with kidney function (Figure S1, Supporting Information). Another high‐resolution dataset from the Kidney Precision Medicine Project, which involves single‐nucleus and single‐cell RNA‐seq of 159 human kidney tissue (0.5 million cells per nuclei) identifies Tnfrsf11a as expressed in Mφ and dendritic cells, emphasizing its cell‐type‐specific role in kidney immune regulation (Figure S2, Supporting Information).

We further characterized the expression of *Tnfrsf11a* and *Zc3h12c* in AKI using the scRNA‐seq data^[^ ^48^ ^]^ of sorted mononuclear phagocytic cells from blood (CD11b^+^Ly6c^+^), kidney (CD11b^+^F4/80^+^), and spleen (CD11b^+^) of healthy and post‐ischemic mice,1‐and 3‐days after kidney IRI (Figure 1F; Figure S3A, Supporting Information). Among 29 999 of these cells, which were defined as mononuclear phagocytes (Figure 1G), Mφ highly expressed *Tnfrsf11a*, particularly those found in the kidney (Figure S3B, Supporting Information), while other kidney immune cells did not express *Tnfrsf11a*. *Tnfrsf11a* expression correlated with the enrichment of genes associated with phagocytosis, chemokines, and monocyte maturation, particularly on days 1 and 3 post‐IRI in blood and kidney Mφ (Figure 1G–I; Figures S4 and S5A, Supporting Information). *Tnfrsf11a* expression was highest in most mature Mφ and kidney Mφ (Figure 1J), highlighting Tnfrsf11a induction during kidney injury. Expression of Zc3h12c was associated with Mφ clusters (Figure 1F). This was also observed in human transcriptome data,^[^ ^61^, ^62^ ^]^ which documents *TNFRSF11A/ZC3H12C* coexpression in various Mφ (Figure 1K). Furthermore, the scRNA‐seq^[^ ^49^ ^]^ of sorted Mφ by CD45^+^TCRb^−^CD19^−^NK1.1^−^Gr‐1^−^CD11b^INT^F4/80^HI^ from murine kidney at 12 h, 1‐, 6‐ and 28‐days post IRI gave a similar result (Figure S5B, Supporting Information). Immunofluorescence of healthy mouse kidneys showed TNFRSF11/ZC3H12C protein in F4/80^+^ Mφ (Figure 1L), and in‐depth bulk RNA‐seq^[^ ^63^ ^]^ confirmed upregulation of Zc3h12c transcripts in Mφ after kidney IRI (Figure 1M). Together, both tissue‐resident and infiltrating mature Tnfrsf11a^+^ Mφ express Zc3h12c.

As *Zc3h12c* is upregulated in the post‐ischemic kidney Mφ, we sought to study the effect of Mφ Zc3h12c in kidneys after IRI in vivo. Here, we specifically opted for the Tnfrsf11a‐Cre line because it preferentially targets activated Mφ and avoids the pitfall of widespread myeloid knockout (as discussed in the Introduction and Discussion). In this strain (Figure S6, Supporting Information), we confirmed for Mφ from various organs while the other regnase family members remained unchanged (Figure 1N; Figure S7, Supporting Information). RNA‐seq results confirmed deletion of the Zc3h12c exon 4, while other exons remained unaffected (Figure S6B, Supporting Information), indicating that the Tnfrsf11a‐Cre^[^ ^42^ ^]^ Zc3h12c‐flox/flox^[^ ^12^ ^]^ conditional knockout (Tnfrsf11a‐Zc3h12c^cKO^) effectively occurred.

### 2.2 Macrophage Zc3h12c Limits Kidney Injury in Various Mouse Models

**Figure 2:**
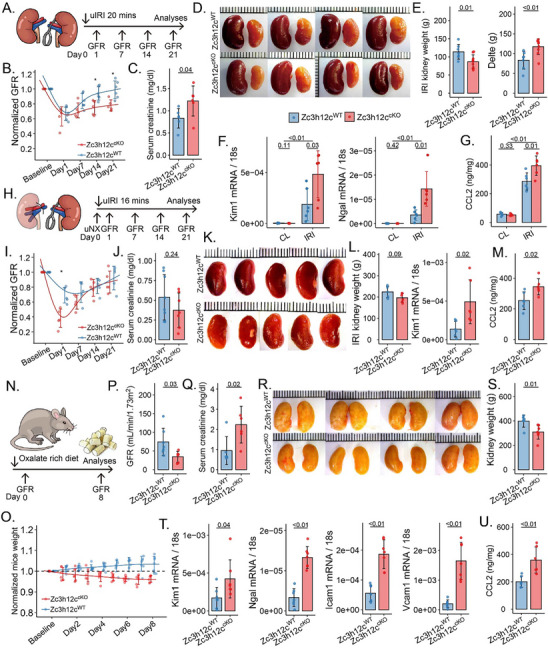
Lack of Zc3h12c in Tnfrsf11a^+^ Mφ aggravates three models of kidney injury. **Model I – unilateral ischemia‐reperfusion injury (uIRI)**: A) A diagram illustrating the set‐up of the experiment uIRI without unilateral nephrectomy (uNX). The uIRI was induced by clamping the kidney pedicle for 20 min. Organs were collected in two different states: a healthy state and on day 21 following IRI. B) The glomerular filtration rate (GFR) normalized to baseline in response to the genotype, C) serum creatinine level, D) kidney macroscopic appearance, E) post‐ischemic kidney weight, contralateral minus post‐ischemic kidney weight (Delta), wildtype (WT) mouse data in blue and Tnfrsf11a‐Zc3h12c^cKO^ mouse data in red. F) The qPCR for the kidney injury markers in contralateral (CL) and IRI kidneys harvested at day 21 after IRI. G) CCL2 protein level in the kidney. **Model II ‐uIRI with uNX**: H) A diagram illustrating the set‐up for uIRI with uNX one week before clamping the kidney pedicle for 16 min. Organs were collected on day 21 following IRI. I) The GFR was normalized to baseline in response to the genotype. J) Serum creatinine level. K) Kidney macroscopic appearance. L) post‐ischemic kidney weight and Kim1 *mRNA* level, and M) CCL2 protein level in the kidney in post‐ischemic kidneys harvested at day 21 after IRI. **Model III – Calcium oxalate (CaOx) nephropathy**: N) Schematic of experimental set‐up for CaOx model. Organs were taken at day 8 after starting the oxalate‐rich diet. O) The mouse weight curves upon change of diet in response to genotype. P) GFR. Q) Serum creatinine level. R) Kidney macroscopic appearance. S) Kidney weight at sacrifice. T) The qPCR for the kidney injury markers and U) CCL2 protein level in kidneys harvested at day 8. All curves were fitted using the cubic spline algorithm. All quantitative data are means ± SD (n = 5–7 biological replicates). Two‐way ANOVA or *t*‐test was employed for the statistical examination.

To investigate the role of Mφ Zc3h12c in acute tissue injury, WT and Tnfrsf11a‐Zc3h12c^cKO^ mice underwent 20 min of kidney uIRI (**Figure** **2**A). In this model of sustained postischemic AKI, all mice experienced an acute drop in GFR at 24 h after reperfusion. However, whereas WT mice largely recovered GFR by 3 weeks, Tnfrsf11a‐Zc3h12c^cKO^ mice did not (Figure 2B,C). At day 21, post‐ischemic kidneys from Tnfrsf11a‐Zc3h12c^cKO^ mice were significantly smaller in weight and size than those from WT mice (Figure 2D,E). Furthermore, the Tnfrsf11a‐Zc3h12c^cKO^ mice post‐ischemic kidney expressed higher levels of kidney injury, inflammation, and fibrosis markers such as *Kim1, Ngal, Ccl2* (as well as the protein levels)*, Ccr2, Ccr5, Icam1, Vcam1, αSMA, Fn*, and *Tgf‐β* (Figure 2F,G; Figure S8, Supporting Information).

Next, we tested 16 min of uIRI combined with contralateral uNX (Figure 2H). The uNX prior to ischemia of the contralateral kidney is discussed to protect the post‐ischemic kidney from prolonged ischemia, as the blood flow through the injured organ is necessarily higher compared to post‐ischemic kidneys with an intact contralateral kidney. In this model, Tnfrsf11a‐Zc3h12c^cKO^ mice displayed a more severe drop in GFR compared to WT mice 1 day post IRI (Figure 2I,J). Despite full recovery of GFR in both Tnfrsf11a‐Zc3h12c^cKO^ and WT mice at day 21, Tnfrsf11a‐Zc3h12c^cKO^ kidneys still showed signs of ongoing injury, with elevated levels of *Kim1 and Ngal* mRNA (Figure 2K,L). Additionally, expression of mRNA for *Tgf‐β, Tgf‐β receptor, Ccl2* (as well as the protein levels, Figure 2M), *Ccr2*, and *Ccr5* were found to be elevated in cKO kidneys (Figure S9, Supporting Information), consistent with increased kidney injury/inflammation in our first uIRI model without uNX.

To further study the role of Mφ Zc3h12c in other type of tissue injury, we fed mice an oxalate‐rich diet to induce nephrocalcinosis‐related progressive kidney injury (Figure 2N). This is a disease model characterized by the deposition of CaOx crystals in the kidneys, progressive tubule damage, and inflammation like those seen in humans with oxalate nephropathy.^[^ ^44^, ^45^ ^]^ In the nephrocalcinosis model, Tnfrsf11a‐Zc3h12c^cKO^ mice lost body weight more rapidly and had a lower GFR and higher serum creatinine levels on day 8 (Figure 2O–Q). By day 8, their kidneys were smaller than those of WT controls (Figure 2R,S). As in the uIRI models, mRNA markers for kidney injury, inflammation, and fibrosis were increased in kidneys from Tnfrsf11a‐Zc3h12c^cKO^ mice (Figure 2T,U; Figure S9B, Supporting Information). These results consistently suggest that Mφ Zc3h12c attenuates kidney injury, inflammation, and fibrosis.

### 2.3 CCR2^+^ Leukocyte Accumulation in the Kidney of Tnfrsf11a‐Zc3h12c^cKO^ Mice

**Figure 3:**
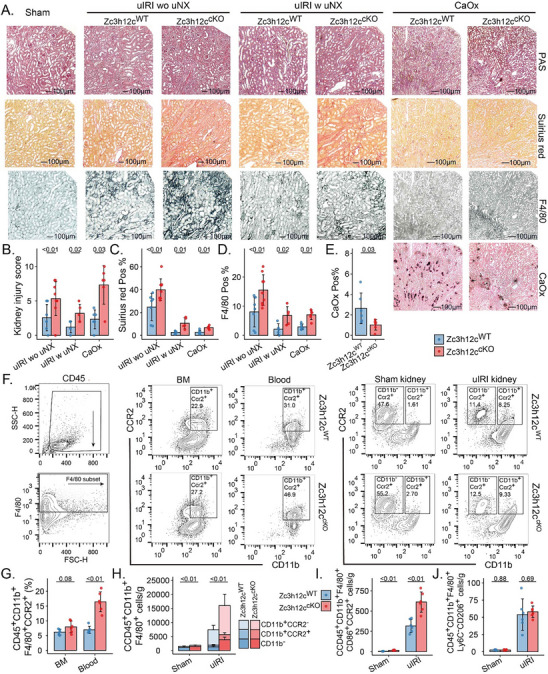
Lack of Zc3h12c in Tnfrsf11a^+^ Mφ increases kidney injury, Mφ infiltration, and fibrosis upon injury. A) Histopathology of Tnfrsf11a‐Zc3h12c^cKO^ and wildtype (WT) mice kidneys in three models of kidney injury: unilateral ischemia‐reperfusion injury (uIRI), uIRI with unilateral nephrectomy (uNX), and calcium oxalate (CaOx) nephropathy. B) Kidney injury score was quantified using Periodic acid‐Schiff staining. C) Interstitial fibrosis was quantified using Sirius red staining. D) Mφ were visualized using F4/80 staining. E) CaOx crystal deposition was quantified using Pizzolato staining. F) Gating strategy for CD45^+^F4/80^+^CD11b^+^CCR2^+^ cells from bone marrow (BM), blood, and in kidney one day after IRI by flow cytometry. G) Percentages for cells with CD45^+^CD11b^+^F4/80^+^CCR2^+^ in BM and blood. Absolute numbers for H) CD45^+^F4/80^+^CD11b^−^CCR2^+^ and CD45^+^CD11b^+^F4/80^+^CCR2^+^ cells, pro‐inflammatory Mφ I) and anti‐inflammatory Mφ J) Mφ in sham and post‐ischemic kidneys of Tnfrsf11a‐Zc3h12c^cKO^ and WT mice one day after IRI. All quantitative data are means ± SD (n = 5–7 biological replicates). Two‐way ANOVA or *t*‐test was employed for the statistical examination.

Histopathology (**Figure** **3**A) revealed that Tnfrsf11a‐Zc3h12c^cKO^ mice were more susceptible to tissue injury (Figure 3B) and developed more interstitial fibrosis/tubular atrophy (IFTA, Figure 3C). F4/80 immunostaining revealed more Mφ (Figure 3D) in Tnfrsf11a‐Zc3h12c^cKO^ mice kidneys, consistent with the higher expression of Mφ‐attracting chemokines even 21 days post‐IRI. This is particularly striking given that Tnfrsf11a‐Zc3h12c^cKO^ mice showed less crystal deposition in the CaOx model (Figure 3E). Indeed, 1‐day post‐IRI, these cKO mice had more tissue injury and inflammation (Figure S10, Supporting Information), characterized by increased CD45^+^CD11b^+^F4/80^+^CCR2^+^ cells in blood (Figure 3F,G) and kidney (Figure 3H; Figure S11, Supporting Information) relative to WT. Additionally, we then gated CD45⁺CD11b⁺F4/80⁺ Mφ into pro‐ and anti‐inflammatory subsets (CD86⁺CCR2⁺ vs Ly6C^−^CD206⁺, Figure S12, Supporting Information). The number of pro‐inflammatory Mφ was higher in cKO kidneys post‐IRI (Figure 3I), whereas anti‐inflammatory Mφ remained unchanged (Figure 3J). Together, these findings indicate that Zc3h12c in Mφ limits kidney injury and inflammation by reducing monocyte recruitment and by restraining Mφ activation toward a pro‐inflammatory phenotype.

### 2.4 Zc3h12c Regulates Macrophage Activation

The scRNAseq data suggested that Tnfrsf11a identifies a subset of blood monocytes and most bone marrow‐derived Mφ (BMDM) with a strong mature phenotype after kidney IRI. Indeed, 20–30% of monocytes, and almost 100% of mature Mφ in healthy kidneys expressed Tnfrsf11a.^[^ ^39^ ^]^ The expression of Tnfrsf11a increased with the migration of Mφ from the bone marrow and blood into the tissue, reaching its highest levels after stimulation with L929 supernatant in vitro (Figure 1I). Therefore, Tnfrsf11a is involved in the maturation of Mφ. We then investigated whether Zc3h12c participates in the activation of Tnfrsf11a‐positive Mφ. To answer this, we cultured BMDM and induced Mφ differentiation with 100 ng mL^−1^ lipopolysaccharide (LPS) and 10 ng mL^−1^ interferon γ (IFNγ), 20 ng mL^−1^ interleukin (IL) 4 or IL10 for 24 h. The expression levels of *Zc3h12a* and *Zc3h12c* were significantly upregulated in M(LPS/IFNγ) Mφ, but *Zc3h12b* and *Zc3h12d* remained unchanged. All *Zc3h12* responded to IL10, while only *Zc3h12a* was upregulated with IL‐4 stimuli (Figure S13, Supporting Information). The deletion of Zc3h12c exon4 in LPS/IFNγ‐treated Mφ was successfully achieved in Tnfrsf11a‐Zc3h12c^cKO^ Mφ as shown in Figure S4 (Supporting Information), which is consistent with a naïve Tnfrsf11a‐Zc3h12c^cKO^ Mφ phenotype. Additionally, we observed a compensatory upregulation of *Zc3h12a* but not *Zc3h12b and Zc3h12d* in Tnfrsf11a‐Zc3h12c^cKO^ LPS/IFNγ‐treated Mφ (Figure S13, Supporting Information).

**Figure 4:**
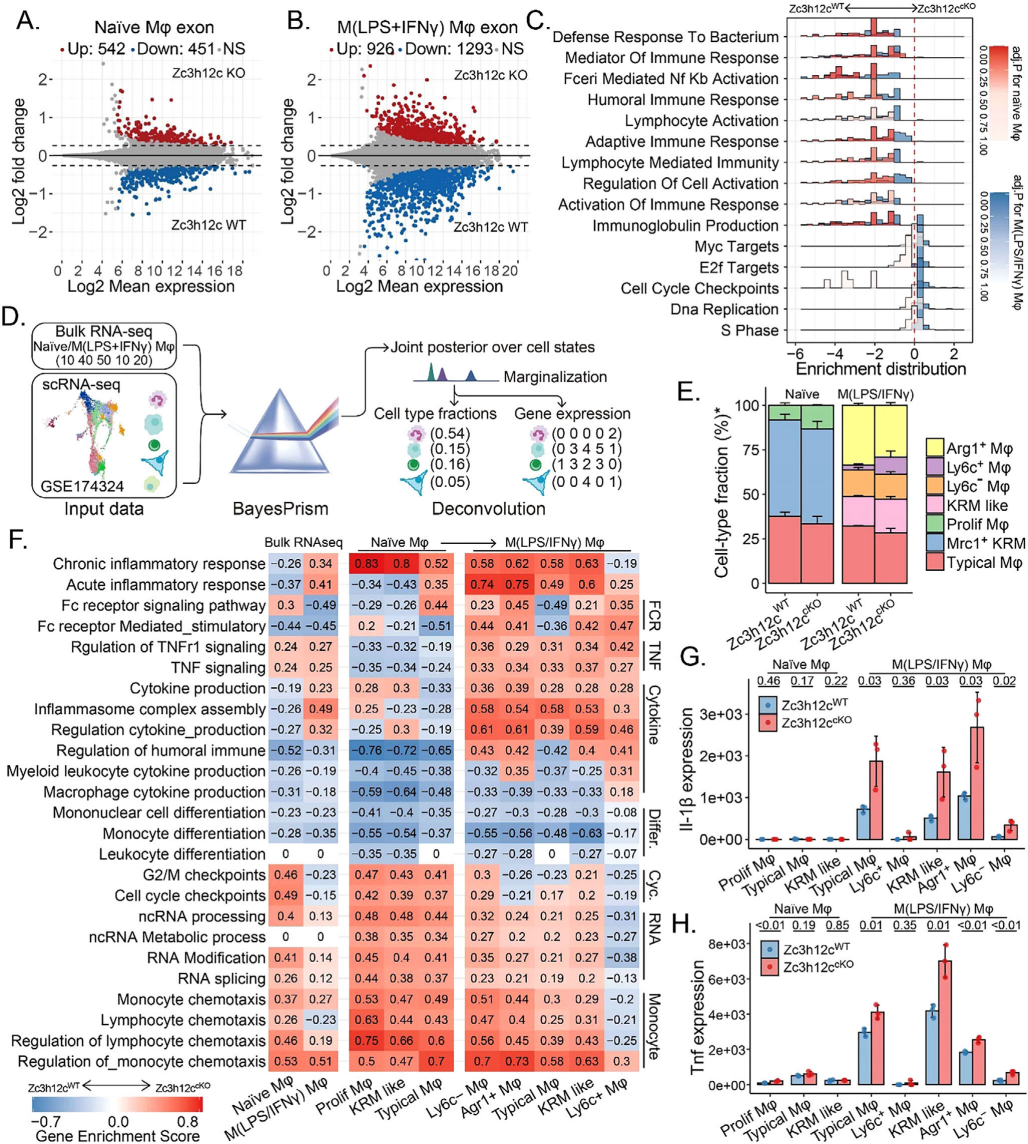
Integrated transcriptomics reconstructs Mφ landscapes. MA plots were created to display the shrink log2‐fold change on exons between Tnfrsf11a‐Zc3h12c^cKO^ and wildtype (WT), A) treatment naïve, and pro‐inflammatory Mφ B) Mφ. Genes with differential expression and an adjusted p‐value lower than 0.05 are identified by the colors red or blue. C) Density ridges for the enriched gene sets. The expression distributions of core‐enriched gene sets for Gene Set Enrichment Analysis are displayed through the density ridge plot, where the gradient red color represents the adjusted *p*‐values for M(LPS/IFN) and blue for naïve Mφ. BayesPrism workflow D) of scRNA‐seq (GSE174324) and bulk RNA‐seq transcriptomic data integration and deconvolution based on BayesPrism to infer joint E) cell type fraction, F) Gene Set Enrichment Analysis, and G,H) selected gene expression in response to Zc3h12c^cKO^ in Tnfrsf11a naïve and M(LPS/IFNγ) Mφ. All quantitative data are means ± SD (n = 3 biological replicates). Student *t*‐test was employed for the statistical examination.

Next, we performed bulk RNA‐seq analysis for Tnfrsf11a‐Zc3h12c^cKO^ Mφ. Lack of Zc3h12c significantly changed Mφ transcriptomes with 926 and 1293 in naïve Mφ (Data S1, Supporting Information) and 542 upregulated and 451 downregulated genes in M(LPS/IFNγ) Mφ (Data S2, Supporting Information) when comparing Tnfrsf11a‐Zc3h12c^cKO^ and WT (**Figure** **4**A,B; Figure S14, Supporting Information). Gene set enrichment analysis (GSEA) showed that the absence of Zc3h12c suppressed the “immune response” in Mφ but promoted DNA repair and cell cycle transcripts only in naïve Mφ (Figure 4C). As this seemed inconsistent with the in vivo results, we used BayesPrism^[^ ^60^ ^]^ to deconvolute bulk RNA‐seq to study the underlying cell subtype (Figure 4D). The Bayesian statistical model jointly infers cell type composition and cell type‐specific gene expression profiles within the bulk RNA‐seq count matrix (Figure S15, Supporting Information). By using this technique, we demonstrated that the lack of Zc3h12c led to more “proliferating Mφ” in naïve Mφ and significantly increased the fraction of pro‐inflammatory Ly6C^+^ Mφ in M(LPS/IFNγ) Mφ (Figure 4E). In addition to determining cell type proportions, BayesPrism offers an opportunity to perform GSEA using cell type‐specific gene expression. Like before, three subtype deconvoluted Zc3h12c^cKO^ naïve Mφ showed more enrichment in transcripts responsible for chemotaxis and cell cycle checkpoints, while Zc3h12c^cKO^ M(LPS/IFNγ) Mφ tend to induce cytokine production (Figure 4F,G), acute inflammatory response, and TNF signaling (Figure 4H; Figure S16, Supporting Information). However, the enrichment of monocyte differentiation was downregulated in all Mφ subtypes. Together, these data support the in vivo finding that Zc3h12c in Mφ suppresses monocyte recruitment and immune activation toward a pro‐inflammatory Mφ phenotype in mice.

### 2.5 Zc3h12c Suppresses Migration and Phagocytosis in Inflammatory Mφ

**Figure 5:**
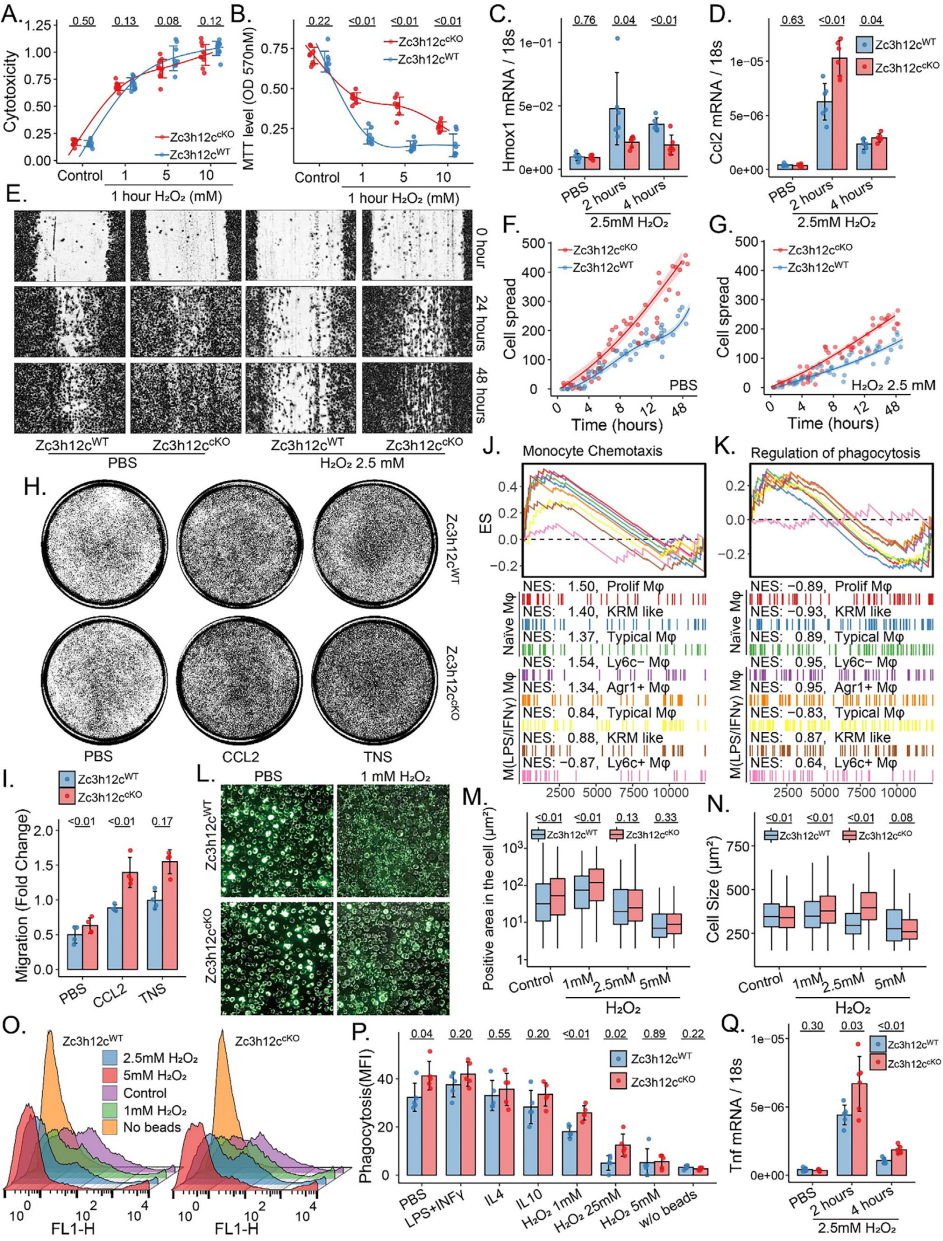
Zc3h12c‐deficiency modulates phagocytosis and migration in Mφ. A) Cytotoxicity (calculated by the LDH OD450) and B) metabolic activity in bone‐marrow‐derived Mφ (BMDM) from Tnfrsf11a‐Zc3h12c^cKO^ and wildtype (WT) mice after being stimulated with 1, 5, and 10 mm H_2_O_2_ for 1 h. C,D) The qPCR for the *Hmox1* and *Ccl2* in BMDM, which were incubated with 2.5 mm H_2_O_2_ for 2 and 4 h. E) Scratch‐induced migration of BMDM from Tnfrsf11a‐Zc3h12c^cKO^ and WT mice after H_2_O_2_ incubation. Images were taken at the 0, 24, and 48 h after scratch in F) control or upon exposure to 2.5 mm H_2_O_2_ G) for 1 h. Cell spread represents the cell number that migrate into the gap. J,K) Transwell migration assay on BMDM exposed to either 10 ng mL^−1^ of CCL2 or tubular cell necrotic soup (TNS) in the bottom well for 12 h. H,I) Selected enrichment plots from Gene Set Enrichment Analysis based on the gene enrichment profiles on Tnfrsf11a‐Zc3h12c^cKO^ compared with WT refer to BayesPrism inferred Mφ subtype. The top portion of the plot shows the running enrichment score (ES). The bottom portion of the plot shows where the members of the gene set appear in the Ranked list of genes, with the normalized ES (NES). For phagocytic capacity, the Mφ were first incubated with or without 0, 1, 2.5, and 5 mm H_2_O_2_ for 1 h, then exposed to fluorescent latex beads with or without rabbit lgG‐FITC, which were quantified by the L) CellPose Neural Convolutional Network and O) flow cytometry, respectively. M) Positive area in the cell represents the fluorescent beads positive area in N) the area of cell segmentation. P) Quantitative analysis by flow cytometry for phagocytic capacity (mean fluorescence intensity, MFI) on a group of 100 ng mL^−1^ LPS+10 ng mL^−1^ IFNγ, 10 ng mL^−1^ IL10, and 10 ng mL^−1^ IL4 for 24 h or 1, 5, and 10 mm H_2_O_2_ incubation for 1 h. Q) The qPCR for *Tnf*. All quantitative data are means ± SD. All quantitative data are means ± SD (n = 5–7 biological replicates). Two‐way ANOVA or *t*‐test was employed for the statistical examination.

Next, we tested the functions of Zc3h12c‐deficient Mφ in vitro. Naïve Tnfrsf11a‐Zc3h12c^cKO^ Mφ exhibited higher metabolic activity following mild and rapid H_2_O_2_ stimulation (1 and 5 mm for 1 h) than WT, but under severe oxidative stress (10 mm or longer), there was no significant difference in cytotoxicity or metabolic activity between genotypes (**Figure** **5**A,B; Figure S13A—D, Supporting Information). *Hmox1* mRNA levels were downregulated but *Ccl2*, *Icam1*, *Vcam1*, *Cxcl1* were upregulated in Tnfrsf11a‐Zc3h12c^cKO^ Mφ upon H_2_O_2_ stimulation (Figure 5C,D; Figure S17, Supporting Information). Furthermore, Tnfrsf11a‐Zc3h12c^cKO^ Mφ migrated faster even after H_2_O_2_ and LPS stimulation (Figure 5E–G; Figure S18, Supporting Information), and were found to be more sensitive to CCL2 and necrotic tubular cell supernatant (Figure 5J,K). This finding was in line with the results from the GSEA, showing that Zc3h12c‐deficiency enhances monocyte/Mφ chemotaxis and migration (Figure 5H). GSEA also indicated that Zc3h12c can modulate the phagocytic capability (Figure 5I), hence we studied Mφ phagocytosis by two techniques (Figure 5L–P, Supplementary materials). Both techniques revealed that the lack of Zc3h12c enhanced the phagocytic capacity in Mφ, to phagocytose more beads under oxidative stress (Figure 5L–P). Moreover, lack of Zc3h12c drives more Tnf mRNA expression (Figure 5Q). Together, Zc3h12c suppresses migration and activation of inflammatory Mφ as well as their phagocytic capacity.

### 2.6 Zc3h12c Deletion Drives Pro‐Inflammatory Mφ Activation

**Figure 6:**
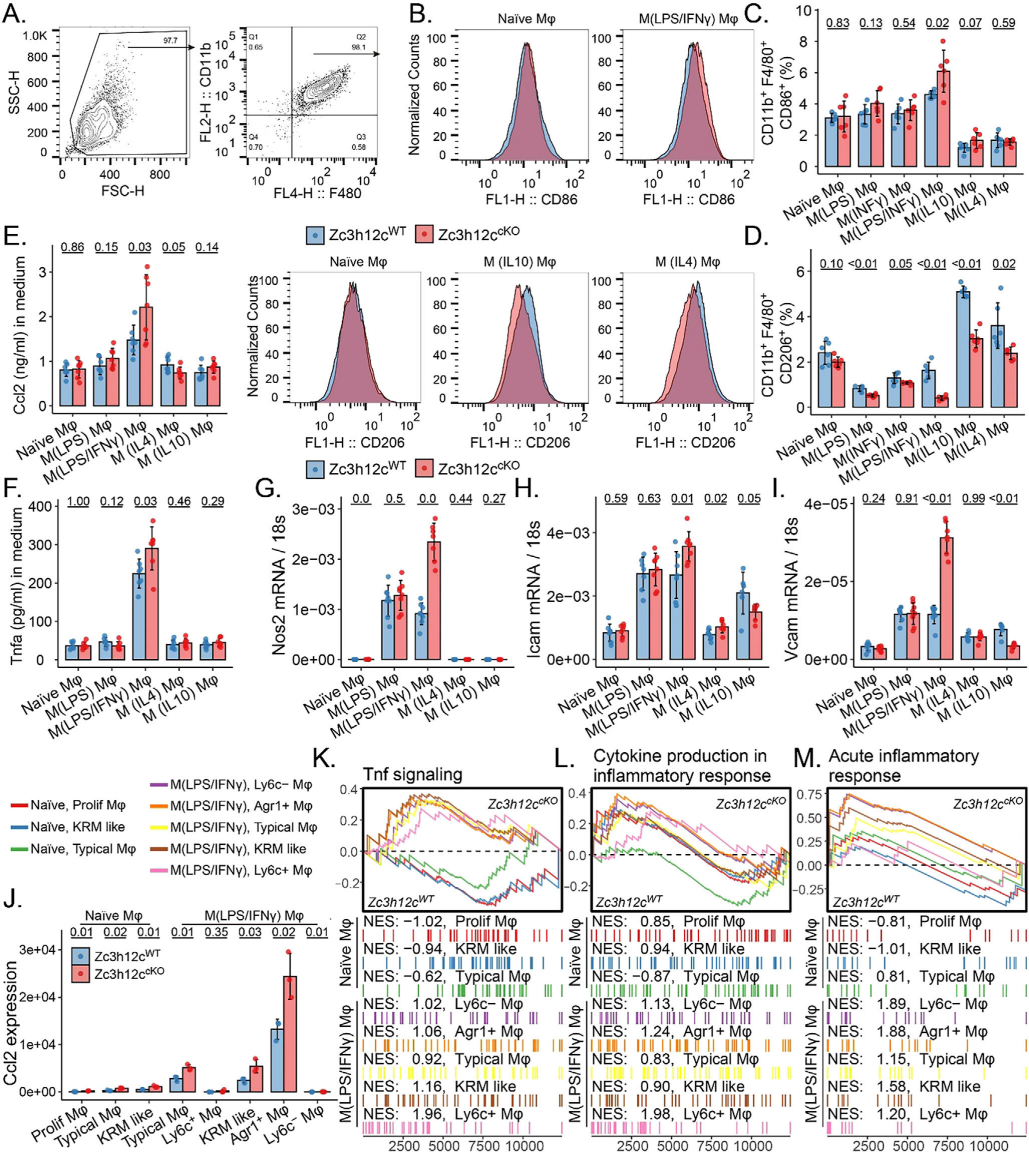
Zc3h12c in Mφ activation. A) Gating strategy for pro‐inflammatory and anti‐inflammatory bone‐marrow‐derived Mφ (BMDM) from Tnfrsf11a‐Zc3h12c^cKO^ and wildtype (WT) mice. BMDM were incubated with 100 ng mL^−1^ LPS+10 ng mL^−1^ IFNγ for 24 h for pro‐inflammatory Mφ activation, 10 ng mL^−1^ IL10 and 10 ng mL^−1^ IL4 for 24 h for anti‐inflammatory Mφ activation. B) Density plot for CD86 in LPS/IFNγ‐stimulated Mφ and CD206 in IL10/IL4‐induced Mφ. C) Proportion for pro‐inflammatory polarized Mφ. D) Proportion of anti‐inflammatory polarized Mφ. E,F) The protein level for Ccl2, Tnf, and G–I) qPCR for the Nos2, Icam1, and Vcam1 in pro‐ and anti‐inflammatory polarized Mφ. J) Ccl2 expression and K–M) Selected enrichment plots from Gene Set Enrichment Analysis based on the gene enrichment profiles on Tnfrsf11a‐Zc3h12c^cKO^ compared with WT refer to BayesPrism inferred Mφ subtype. The top portion of the plot shows the running enrichment score (ES) for the gene set. The bottom portion of the plot shows where the members of the gene set appear in the Ranked list of genes, with the normalized ES (NES). All quantitative data are means ± SD (n = 6–9 biological replicates). Student *t*‐test was employed for the statistical examination.

Next, we sought to validate Mφ activation in Tnfrsf11a‐Zc3h12c^cKO^ BMDM in vitro (**Figure** **6**A,B). As a result, the proportion of CD86^+^ pro‐inflammatory Mφ was increased in Tnfrsf11a‐Zc3h12c^cKO^ Mφ compared to WT when stimulated with LPS and IFNγ (Figure 6C). Consistently, the fraction of alternatively activated Mφ was also lower in the Tnfrsf11a‐Zc3h12c^cKO^ group, regardless of whether IL4 or IL10 was used (Figure 6D). The Tnfrsf11a‐Zc3h12c^cKO^ M(LPS/IFNγ) Mφ expressed higher levels of pro‐inflammatory mediators, including Ccl2, *Tnfa, Ccr2/5*, *Il1b*, *Icam1*, and *Vcam1*, particularly in LPS/IFNγ‐stimulated Mφ (Figure 6E–I; Figure S18, Supporting Information), consistent with the BayesPrism results (Figure 6J; Figure S19, Supporting Information). GSEA indicated that the Zc3h12c deletion might activate TNF signaling (Figure 6K), cytokine production, and acute inflammatory response in M(LPS/IFNγ) Mφ (Figure 6L,M). Overall, the results suggest that Zc3h12c regulates Mφ activation.

### 2.7 Zc3h12c Regulates Alternative Splicing

**Figure 7:**
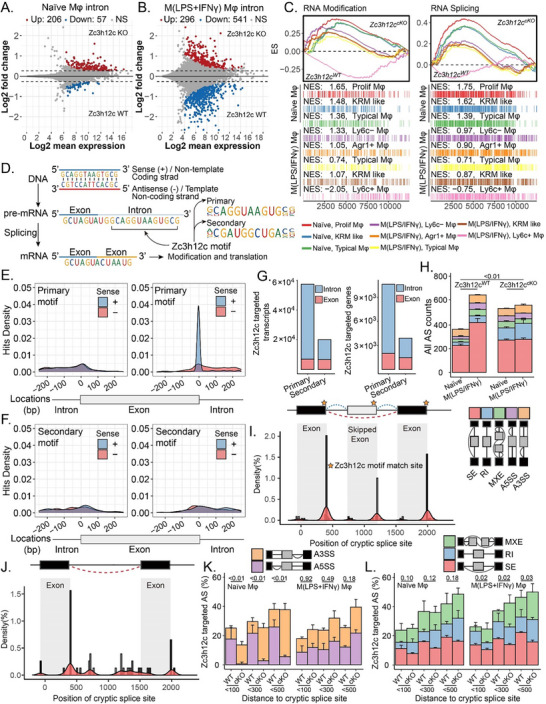
Zc3h12c targeting pre‐RNA and alternating alternative splicing. MA plots were created to display the shrink log2 fold change on exons between Tnfrsf11a‐Zc3h12c^cKO^ and wildtype (WT) naïve A) and M(LPS/IFNγ) Mφ B). Genes that have undergone differential expression testing and have an adjusted *p*‐value lower than 0.05 are identified by the colors red or blue. C) Selected enrichment plots from Gene Set Enrichment Analysis based on the gene enrichment profiles on Tnfrsf11a‐Zc3h12c^cKO^ compared with WT refer to BayesPrism inferred Mφ subtype. The top portion of the plot shows the running enrichment score (ES) for the gene set. The bottom portion of the plot shows where the members of the gene set appear in the Ranked list of genes, with the normalized ES (NES). D) Primary and secondary sense strand motif of Zc3h12c. Zc3h12c's E) primary and F) secondary motifs mostly bind introns, while the primary motif specifically targeting the junction between intron and exon. G) The number of motifs hit on the transcripts of genes, illustrated for introns and exons. The plots of the counts of H) alternative splicing events. Density plot for the Zc3h12c motif targeted location on the I) skipped exon and G) retained intron. K,L) Proportions of Zc3h12c motif targeted alternative splicing in the Zc3h12^cKO^ and WT naïve and M(LPS/IFNγ) Mφ. The *x*‐axis represents the distance (bp) between the Zc3h12c primary motif‐matched nucleotide sequences position and the cryptic splice site. All quantitative data are means ± SD. SE, Exon; MXE, mutually exclusive exon; RI, retained intron; A3(5)SS, alternative 3(5)'‐splice site. All quantitative data are means ± SD (n = 3 biological replicates). Student *t*‐test was employed for the statistical examination.

To identify the mechanism by how Zc3h12c regulates Mφ functions, we analyzed the Mφ transcriptome for RNA splicing. Indeed, loss of Zc3h12c altered intron retention events. (**Figure** **7**A,B). In naïve Mφ, 206 introns had increased inclusion and 57 had decreased inclusion in cKO versus WT (Figure 7A,B). Upon LPS/IFNγ stimulation, 296 introns showed increased retention and 541 showed decreased retention in cKO. These numbers are far from a 1:1 distribution, suggesting a selective effect on splicing patterns. GSEA suggested that the lack of Zc3h12c might modify RNA and RNA splicing (Figure 7C), i.e., RNA regulation, in Zc3h12c‐deficient pro‐inflammatory Mφ. Using RNA‐SELEX,^[^ ^64^ ^]^ we identified the primary and secondary motifs of Zc3h12c as 3′‐GCAGGUAAGUGCG‐5′ and 3′‐ACGAUGGCUGACC‐5′, respectively (Figure 7D). Interestingly, we observed that Zc3h12c primary motifs tend to specifically target the sense‐strands at the exon‐intron‐junction (Figure 7E) in contrast to the non‐specific targeting pattern observed for the secondary motif (Figure 7F). The Zc3h12c primary motif was found in 50545 introns and 13466 exons, corresponding to 8632 transcripts from 2455 genes (Figure 7G; Data S3–S6, Supporting Information). The exon‐intron‐junction is essential for pre‐RNA splicing,^[^ ^65^ ^]^ hence we studied the role of Zc3h12c on alternative splicing. By counting the events of both skipped exon and retained introns, we found that WT M(LPS/IFNγ) Mφ exhibited a higher number of skipped exons and retained more introns than treatment naïve Mφ (Figure 7H, P<0.01). However, Zc3h12c^cKO^ Mφ displayed similar numbers of skipped exon events in naïve and M(LPS/IFNγ, P = 0.46) Mφ, indicating that the presence of Zc3h12c leads to more skipped exon events in Mφ upon activation. Conversely, when Zc3h12c was absent, a higher probability of retained intron was observed, regardless of Mφ activation state (p = 0.15).

In order to further locate Zc3h12c‐related alternative splicing, we examined the matching position for the Zc3h12c primary motif. Mapping the primary Zc3h12c motif genome‐wide revealed enrichment at exon–intron junctions: specifically, at the 3′ splice sites of upstream exons involved in alternative splicing (Figure 7I) and at junctions flanking retained introns (Figure 7J). We examined alternative splicing events that specifically possess Zc3h12c primary motif‐matched nucleotide sequences (Figure 7K,L). Notably, although the frequency of the alternative 3(5)' splice site [A3(5)SS] is relatively low compared to the skipped exon, Zc3h12c targeted almost 40% of the A3(5)SS. In Zc3h12c cKO Mφ (naïve state), we observed more A3SS and fewer A5SS events, whereas in WT the pattern was reversed. This bias was seen in naïve Mφ but not after LPS/IFNγ stimulation. Moreover, 20% of the skipped exons, retained intron, and mutually exclusion exons events were targeted by Zc3h12c (Figure 7L). Thus, the presence of Zc3h12c led to more skipped exon but less retained intron and mutually exclusion exons events. This supports the hypothesis that Zc3h12c specifically targeted pre‐RNA and regulated alternative splicing by counterpoising the proportion of various AS events, and this regulation appears particularly dependent on the activation of Mφ.

### 2.8 Zc3h12c Regulates STAT1 Alternative Splicing

**Figure 8:**
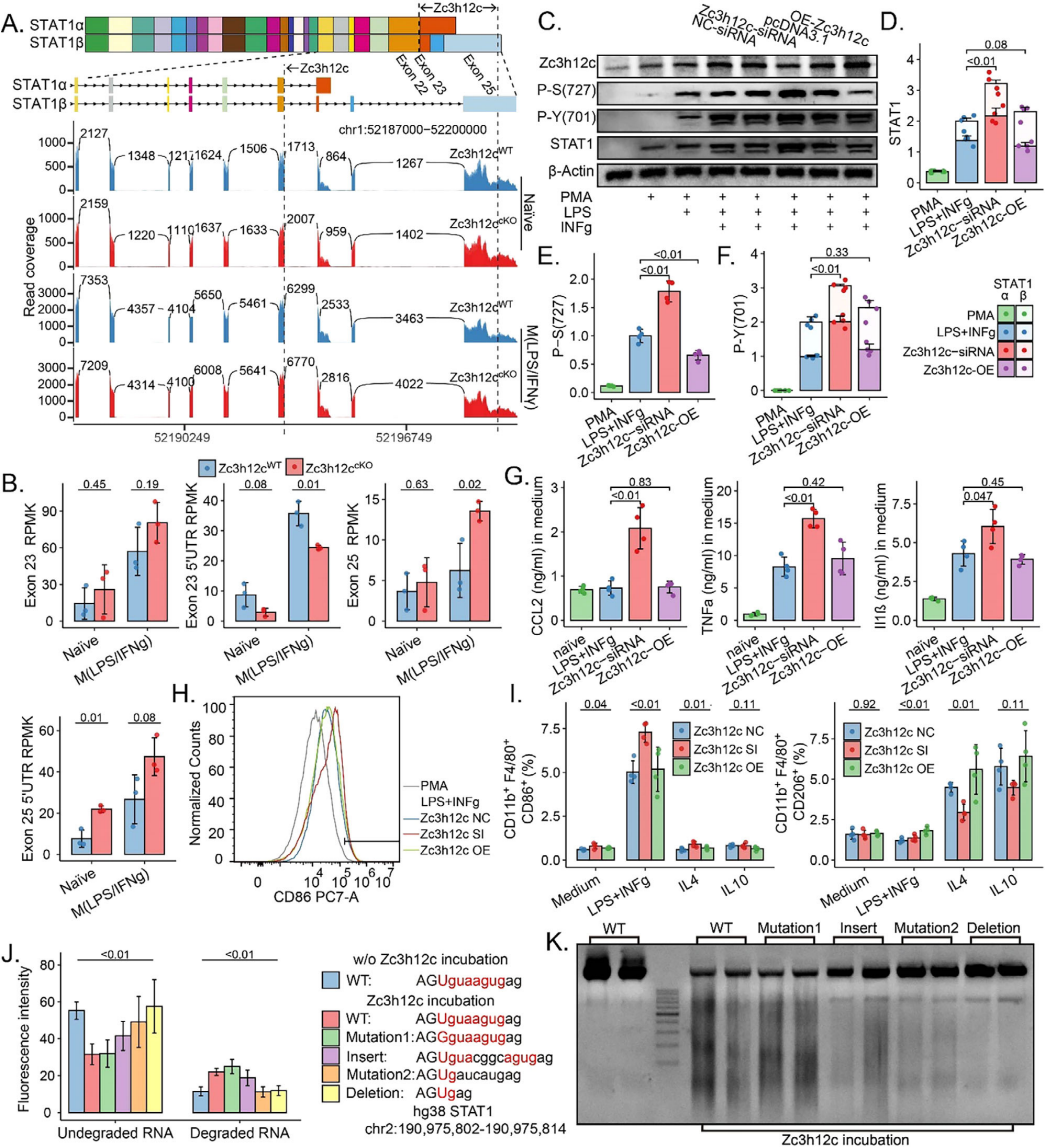
Zc3h12c Regulates STAT1 Expression, Phosphorylation, and Macrophage Function. A) Genomic organization of the STAT1 locus and RNA‐seq read coverage at the STAT1 locus in WT and Zc3h12c^cKO^ bone marrow‐derived macrophages under naive or M(LPS/IFNγ) conditions. The top panel illustrates the gene structure of STAT1α and STAT1β. Below are RNA‐seq tracks showing read coverage for the indicated genotypes and conditions. Numbers above peaks indicate peak heights. The vertical dashed line indicates the Zc3h12c targeting motif site. B) Quantitative analysis of Exon 23, Exon 23 5′UTR, and Exon 25 reads per kilobase million (RPMK) in wild type (WT) and Zc3h12c^cKO^ BMDMs under naive or M(LPS/IFNγ) conditions. C) Western blot analysis of Zc3h12c, p‐S(727)‐STAT1, p‐Y(701)‐STAT1, STAT1, and β‐Actin in BMDMs transfected with indicated siRNAs or overexpression plasmids and treated with phorbol 12‐myristate 13‐acetate (PMA) only or LPS+IFNγ as indicated. D–F) Quantification of STAT1, p‐S(727)‐STAT1, and p‐Y(701)‐STAT1 protein levels normalized to β‐Actin from (C). Data are presented as mean ± SEM (n = 3). p‐values are indicated above the bars (unpaired *t*‐test). G) ELISA analysis of TNFα, CCL2, and IL‐1β levels in culture supernatants of BMDMs transfected with indicated siRNAs or overexpression plasmids and treated with PMA or LPS+IFNγ. H) Flow cytometry histogram showing CD86 expression on macrophages transfected with indicated Zc3h12c siRNAs or overexpression plasmids and treated with PMA or LPS+IFNγ.I) Flow cytometry analysis of CD86+ and CD206+ populations in CD11b+F4/80+ macrophages under Medium, LPS+IFNγ, IL‐4, or IL‐10 conditions with Zc3h12c negative control (NC) siRNA, siRNA (SI), or overexpression (OE) plasmids of Zc3h12c. J) In vitro RNA degradation assay showing fluorescence intensity of undegraded and degraded RNA with or without WT Zc3h12c incubation and with different STAT mutants. The sequences of the RNA substrate and Zc3h12c mutants are listed. K) Representative gel image of in vitro RNA degradation assay with WT and mutant Zc3h12c incubation. All quantitative data are means ± SD (n = 3–9 biological replicates, n = 4 technical replicates for RNA decay assays). Two‐way ANOVA or *t*‐test was employed for the statistical examination.

To further investigate the mechanisms underlying the phenotypes observed in Zc3h12c^cKO^ induced macrophage activation, we sought to identify downstream targets of Zc3h12c. Considering the known roles of Zc3h12 proteins in RNA regulation and their potential impact on alternative splicing, we focused on STAT1, a key transcription factor involved in macrophage function and inflammatory responses. To examine whether Zc3h12c regulates STAT1 expression, we first examined STAT1 locus transcript levels in WT and Zc3h12c^cKO^ using RNA‐sequencing. RNA‐seq analysis revealed reduced read coverage across the STAT1 locus, particularly within and around Exon 23 and its 5′ UTR region in Zc3h12c^cKO^ Mφ compared to WT cells, especially upon M(LPS/IFNγ) stimulation (**Figure** **8**A,B; Figure S14E, Supporting Information). Western blots showed that siRNA‐mediated Zc3h12c knockdown in PMA‐differentiated THP‐1 Mφ increased total STAT1 protein and phosphorylation at Ser727 and Tyr701 after LPS+IFNγ stimulation (Figure 8C). Intriguingly, while Zc3h12c knockdown also led to a significant increase in the proportion of the STAT1α isoform (Figure 8D–F). Conversely, while overexpression of Zc3h12c showed a trend toward increased STAT1β proportion and phosphorylation, this effect was not statistically significant (Figure 8C–F).

Functionally, Zc3h12c deficiency, achieved by siRNA knockdown (Figure S20, Supporting Information), resulted in increased secretion of pro‐inflammatory cytokines TNFα, CCL2, and IL‐1β upon PMA or LPS+IFNγ stimulation, while Zc3h12c overexpression did not significantly alter the secretion of these cytokines (Figure 8G). Flow cytometry analysis further showed that Zc3h12c knockdown increased the CD11b+F4/80+CD86+ proportion in LPS+IFNγ‐stimulated PMA‐differentiated THP‐1 Mφ (Figure 8H,I). Finally, to biochemically validate direct RNA targeting, we performed in vitro degradation assays using recombinant ZC3H12C protein and synthetic STAT1 RNA substrates: wild‐type sequences containing its binding motif (exon 22 and intron 22 junction, chr2:190975802‐190975814), motif‐mutated variants, insertion, and deletion controls. The assays indicated that Zc3h12c possesses RNA degradation activity, which was modulated by specific mutations in Zc3h12c (Figure 8J,K), suggesting a potential direct mechanism for Zc3h12c‐mediated regulation of STAT1 mRNA. Collectively, these data indicate that Zc3h12c positively regulates STAT1 expression, alternative splicing, and phosphorylation, thereby promoting pro‐inflammatory cytokine production and M1 macrophage polarization.

## 3 Discussion

We had hypothesized that Mφ Zc3h12c would act as a regulator of Mφ activation and therefore modulate injury and repair. Our results obtained from three different kidney injury models in mice with a Mφ‐specific deletion of Zc3h12c consistently show that Mφ Zc3h12c limits necroinflammation and progressive tissue atrophy. Mechanistically, Zc3h12c influences Mφ activation and numerous Mφ functions by increasing the proportion of targeted skipped exon events and decreasing the probability of targeted retained intron events of transcripts, i.e., alternative splicing. This regulation targeted STAT1, with Zc3h12c deficiency skewing its splicing toward the hyperinflammatory STAT1α isoform and driving macrophage activation and cytokine production. Thus, within the Regnase family, Zc3h12c has a Mφ‐state‐dependent immunoregulatory role: its absence leads to increased inflammation and injury.

### 3.1 RNA‑Binding Proteins and Alternative Splicing in Immune Cells

Post‐transcriptional regulation by RNA‑binding protein (RBP) is a key layer of immune control, often exerted through alternative splicing. In immune cells, RBP‐mediated splicing governs gene expression programs by modulating AS by pervasive intron retention and exon‐skipping in activated leukocytes. For example, memory CD4+ T cells exhibit dynamic intron retention patterns affecting transcripts like IL7R and FOXP3 during activation and differentiation.^[^ ^66^, ^67^ ^]^ Similarly, activated Mφ exhibit widespread intron retention changes, wherein inflammatory transcripts lose retained introns upon stimulation, enabling rapid protein production. This process involves specific RBPs as key AS modulators, as demonstrated by Liu et al. using deep RNA‐seq of human monocytes and Mφ to show that the splicing regulator MBNL1 is a major driver of exon usage changes in macrophage activation.^[^ ^68^ ^]^ Other general splicing factors are likely involved. Notably, the KH‐domain splicing factor QKI (Quaking) is a master regulator of alternative splicing during monocyte‐to‐macrophage differentiation.^[^ ^69^ ^]^ QKI controls hundreds of cassette exons and intron retention events in developing myeloid cells, binding ACUAA motifs in the flanking introns to suppress or enhance exon inclusion (e.g., in Bicd2). Indeed, many CCCH‐zinc finger RBPs (besides Zc3h12c) are expressed in myeloid cells and may indirectly influence spliceosome activity. Beyond Zc3h12c itself, several RBPs such as hnRNP M (repressor of inflammatory splicing),^[^ ^70^ ^]^ and SR‐proteins like SRSF1^[^ ^71^ ^]^ and SRSF3^[^ ^72^ ^]^ (which influence macrophage inflammatory transcripts and STAT1 splicing) stand out as candidates to control the exon‐intron balance in Mφ. Our identification of Zc3h12c alongside established regulators reveals a network of splicing modulators.

### 3.2 Tnfrsf11a in Mφ and Tissue Inflammation

Tnfrsf11a encodes the receptor RANK, originally identified for its role in osteoclast differentiation, but it is also expressed on diverse immune cells, including dendritic cells, tissue Mφ, and microglia.^[^ ^39^, ^73^ ^]^ Mφ are intimately entwined with the Tnfrsf11‐Tnfrsf11a (RANKL‐RANK) ligand‐receptor system as both osteoclast precursors and bone‐supporting cells. Osteoclasts derive from the monocyte– Mφ lineage: circulating monocytes and marrow Mφ fuse to form multinucleated osteoclasts under the influence of Tnfrsf11.^[^ ^74^ ^]^ Although they are classically known for osteoimmunologic roles, both are expressed in the kidney and modulate local immune responses. Developmentally, Tnfrsf11a is detectable in embryonic kidneys,^[^ ^39^ ^]^ and in adults Tnfrsf11a is induced on myeloid precursors by factors like M‐CSF.^[^ ^75^ ^]^ In healthy kidney tissue, resident Mφ and dendritic cells are likely to receive local Tnfrsf11 signals to maintain homeostasis. Physiologically, the Tnfrsf11‐Tnfrsf11a axis in Mφ biases toward anti‐inflammatory, reparative phenotypes: Tnfrsf11‐Tnfrsf11a engagement upregulates IL‐10 and other anti‐inflammatory markers.^[^ ^76^ ^]^ Therefore, Tnfrsf11a likely helps restrain overactive innate immunity in normal kidney tissue, maintaining Mφ in a more regulatory state and preventing unwarranted fibrosis. In acute kidney injury, Tnfrsf11‐Tnfrsf11a signaling appears renoprotective. In murine kidney IRI models, blockade of Tnfrsf11a worsens injury, whereas recombinant Tnfrsf11 improves kidney function.^[^ ^77^ ^]^ In type 2 diabetes nephropathy, Chen et al. demonstrated that both Tnfrsf11 and Tnfrsf11a are overexpressed in glomeruli and tubulointerstitium, concomitant with NF‐κB activation and elevated CCL2.^[^ ^78^ ^]^ In human glomerular diseases such as focal segmental glomerulosclerosis, IgA nephropathy, and membranous nephropathy, podocyte and mesangial cells upregulate Tnfrsf11a.^[^ ^79^ ^]^ Our findings establish Tnfrsf11a⁺ Mφ as key regulators of kidney inflammation via Zc3h12c‐dependent RNA splicing. By leveraging Tnfrsf11a‐Cre specificity, we uncovered a novel mechanism where Tnfrsf11a‐expressing Mφ modulate tissue repair via STAT1, offering new therapeutic targets for inflammatory kidney diseases.

### 3.3 Mφ Zc3h12c Limits Necroinflammation and Tissue Remodeling

While our data link Zc3h12c to post‐ischemic kidney injury, related family members are already known to influence ischemic damage in multiple organs. In human ischemic heart disease, Zc3h12a is dramatically elevated, and transgenic overexpression of Zc3h12a in mouse heart protects against inflammation‐induced NF‐κB activation.^[^ ^80^ ^]^ Niu et al. also showed that Zc3h12a markedly improved outcomes after myocardial infarction, reducing inflammation and adverse remodeling.^[^ ^81^ ^]^ Another mouse model showed cardiomyocyte CCL2 triggers Zc3h12a upregulation, and deficiency of Zc3h12a worsens cardiac remodeling and dysfunction.^[^ ^80^, ^82^ ^]^ Likewise, in the brain, Zc3h12a exerts neuroprotective effects: mice lacking Zc3h12a have larger infarcts and blood–brain barrier breakdown after stroke, whereas upregulating Zc3h12a increases ischemic tolerance.^[^ ^83^, ^84^, ^85^ ^]^ Conversely, Zc3h12a deficiency in the brain worsens stroke injury.^[^ ^86^ ^]^ These findings indicate that Zc3h12 family RNases help resolve inflammation and cell death in multiple ischemic contexts. Importantly, in addition to the role of Zc3h12c in ischemic injury, it exhibits distinct immunoregulatory roles compared to the other Regnases. Liu et al.^[^ ^23^ ^]^ conducted a comprehensive analysis of the role of Zc3h12c in inflammation, using both global Zc3h12c knockout and conditional knockout on myeloid cells. Their results suggested that Zc3h12c contributes to inflammation by enhancing TNF in Mφ and reducing IL6 in plasmacytoid dendritic cells, thus the role of Zc3h12c is cell type‐specific. Our results in prior work and this study are consistent with this: Zc3h12c does not significantly influence the inflammatory activation induced by only LPS^[^ ^12^ ^]^ and alternative activation induced by IL4 and IL10 in Mφ, which is consistent with Liu's results.^[^ ^23^ ^]^ In our hands, Zc3h12c‐deficient Mφ are more responsive to IFNγ+LPS stimulation, leading to more CD86⁺ pro‐inflammatory cells, an effect not seen with LPS alone. This suggests Zc3h12c modulates macrophage sensitivity to specific cytokine contexts.Liu et al. also reported that Zc3h12c can degrade IL‐6 and Zc3h12a mRNA.^[^ ^23^ ^]^ Our motif analysis suggests Zc3h12c binds to a widespread set of exon–intron junctions, so its targets include many genes, not just those traditionally linked to inflammation. Thus, Zc3h12c's anti‐inflammatory effects likely involve complex regulation of RNA processing or signaling, beyond direct cytokine mRNA degradation. Overall, both global and macrophage‐specific Zc3h12c deficiency highlight the importance of this RNase in maintaining macrophage‐mediated immune homeostasis.

*The role of Zc3h12c in regulating pre‐mRNAs and alternative splicing* emerges as a potential mechanism underlying its modulation of macrophage activation and inflammatory responses. Like Zc3h12a, which endonucleolytically degrades mRNAs of *Il1β*, *Il6* and *c‐Rel*,^[^ ^11^, ^27^, ^82^, ^88^ ^]^ we propose that Zc3h12c may also target inflammatory transcripts. However, our data suggest Zc3h12c preferentially engages pre‐mRNAs and splicing regulation. Our findings demonstrate that Zc3h12c deficiency skews the balance of splicing events, favoring retained introns over skipped exons, particularly in transcripts critical for macrophage chemotaxis and cytokine signaling. For example, prior studies^[^ ^21^, ^89^ ^]^ suggest Zc3h12c regulates NF‐κB p65 (RelA). We identified a Zc3h12c binding motif near the junction of exon 2 and intron 2 of NF‐κB p65 (chr11:65662157‐65662205; Data S3–S6, Supporting Information), which may influence its splicing or stability. The primary Zc3h12c motif's enrichment at exon–intron boundaries suggests Zc3h12c may directly influence splice‐site selection, akin to other RNA‐binding proteins that fine‐tune transcript processing under stress conditions.^[^ ^64^, ^65^ ^]^ It likely acts post‐transcriptionally to select against certain isoforms via its RNase activity, thereby indirectly shifting the balance of spliced transcripts. This is distinct from bona fide splicing regulators, which bind pre‐mRNA to modulate intron excision or exon inclusion. Importantly, the genes most affected by Zc3h12c loss were enriched in TNF signaling and leukocyte migration pathways, consistent with the heightened pro‐inflammatory phenotype of Zc3h12c‐deficient Mφ. Notably, the Zc3h12a mRNA level was upregulated in Zc3h12c absent, suggesting a compensatory feedback mechanism within the Regnase family. For instance, recent work by Bataclan et al.^[^ ^87^ ^]^ demonstrated that in mast cells, depletion of Zc3h12c significantly increased Zc3h12a expression. This observation is similar to our finding of elevated Zc3h12a in Zc3h12c‐deficient Mφ, yet inflammatory transcripts remained largely changed, indicating partial but incomplete compensation. However, Zc3h12c's mechanism, which relies on RNase‐mediated degradation to influence the balance of spliced transcripts, highlights a potentially divergent regulatory strategy among Regnase proteins, where transcript stability (direct/indirect splicing control) serves as a key regulatory node in macrophage‐driven inflammation.

*Zc3h12c regulates STAT1 expression and splicing*, influencing the balance between its two major isoforms: STAT1α (full‐length) and STAT1β (lacking the C‐terminal transactivation domain). STAT1α drives pro‐inflammatory gene transcription, while STAT1β acts as a dominant‐negative regulator.^[^ ^90^, ^91^, ^92^ ^]^ Our data show that Zc3h12c deficiency increases STAT1α levels and phosphorylation, thus amplifying TNFα and CCL2 production. Mechanistically, Zc3h12c motifs near STAT1 exon 23, promoting exon skipping to favor STAT1β generation, thereby limits STAT1α‐mediated hyperactivation. This splicing regulation is critical, as unchecked STAT1α activity exacerbates macrophage polarization toward a pro‐inflammatory state.^[^ ^93^ ^]^ Additionally, our in vitro RNA decay assays suggest Zc3h12c's RNase activity may directly degrade STAT1 transcripts, as evidenced by RNA decay assays, implying dual control over STAT1 expression and splicing. Beyond Zc3h12c, the Epstein–Barr virus SM protein is a well‑characterized viral regulator: SM binds to the STAT1 transcript and promotes usage of a cryptic 5′ splice site, thereby favoring STAT1β production and dampening STAT1α‑mediated responses.^[^ ^94^ ^]^ Aside from EBV, one report indicated that the EBV‐encoded EB2 protein (homologous to SM) requires SRSF3^[^ ^72^ ^]^ to disrupt STAT1α production.^[^ ^95^ ^]^ These findings position Zc3h12c as a gatekeeper of STAT1 signaling, balancing its isoforms to restrain excessive inflammation—a function distinct from Zc3h12a, which primarily targets IL‐6 and c‐Rel mRNAs.^[^ ^11^, ^21^ ^]^ Direct targeting of ZC3H12C itself remains speculative given its early‐stage characterization, broad RNA‐binding specificity (> 8000 motif‐containing transcripts), and pleiotropic effects. However, our work identifies downstream actionable nodes: the STAT1α:β isoform ratio—imbalanced in Zc3h12c deficiency—could serve as a biomarker for macrophage activation or fibrotic propensity. More pragmatically, splice‐switching oligonucleotides (SSOs) directly modulating STAT1 splicing present a viable therapeutic strategy, analogous to STAT3‐targeting siRNAs in oncology trials (e.g., DCR‐STAT3 siRNA, NCT06098651). Such nucleic acid‐based approaches could correct pathological STAT1 isoform ratios without needing to engage the upstream Zc3h12c machinery. Future studies should prioritize validating STAT1 SSOs in preclinical kidney injury models while exploring ZC3H12C‐independent biomarkers (e.g., STAT1α:β) in human cohorts where proteomic profiling currently excludes ZC3H12C.

### 3.4 Limitation and Outlook

While this study provides robust evidence for Zc3h12c‐mediated regulation of STAT1 alternative splicing and inflammation, several limitations warrant consideration. Definitive proof of direct RNA binding requires further validation; conventional assays using full‐length Zc3h12c are confounded by its RNase activity, necessitating future studies employing isolated zinc finger domains for targeted transcript capture. BayesPrism deconvolution depends on scRNA‐seq reference quality, where rare macrophage states may be underrepresented—its application across diverse tissues thus requires continued validation. Furthermore, reliance on LPS+IFNγ stimulated BMDMs rather than kidney‐resident Mφ presents significant limitations, as BMDMs lack exposure to in vivo renal cues (e.g., hypoxia, uremic toxins) that may shape Zc3h12c function. Consequently, while informative, these results should be interpreted with caution regarding their direct applicability to the complex in vivo kidney microenvironment. Building on our findings, future studies could prioritize therapeutic strategies to modulate the Zc3h12c‐STAT1 axis—such as splice‐switching oligonucleotides correcting STAT1 isoform imbalances or small molecules enhancing Zc3h12c's RNase activity—to mitigate macrophage‐driven inflammation in kidney disease and beyond. Expanding beyond STAT1, exploring Zc3h12c's regulation of parallel inflammatory pathways (e.g., NF‐κB) and its crosstalk with compensatory Zc3h12a networks will elucidate broader roles in fibrosis, cancer, and autoimmune disorders, positioning Zc3h12c as a multifaceted therapeutic node in inflammation biology.

In summary, Zc3h12c plays a significant role in post‐ischemic kidney injury and macrophage function. It modulates macrophage activation (limiting an inflammatory phenotype) and migration. Zc3h12c deletion in Tnfrsf11a‐Cre Mφ exacerbated kidney injury by recruiting more Mφ; thus, contributing to the early injury phase and the transition from AKI to CKD. This effect is mediated through alternative splicing, particularly via dysregulated STAT1 processing in which Zc3h12c deficiency promotes the hyperinflammatory STAT1α isoform, amplifying macrophage activation and cytokine release. Taken together, our data demonstrate a functional role of Zc3h12c in tissue injury and inflammation, and Zc3h12c might represent a new potential therapy target in this context.

## 4 Experimental Section

### Transgenic Mice

To study the role of Zc3h12c in the kidney injury, a series of genetically modified C57BL/6J background mice were developed, including the transgenic Tnfrsf11a‐Cre, and Zc3h12c floxed (Zc3h12c^flox/flox^) mice. The Zc3h12c^flox/flox^ mice^[^ ^12^ ^]^ carry a targeting vector in the intron between exon 3 and exon 4 with a selection cassette flanked by flippase recognition targeting sites, while exon 4 was flanked by a loxP‐site. The splice acceptor together with a poly‐A signal leads to a mature and spliced fusion mRNA of the β‐galactosidase gene sequence in exon 3. The poly‐A signal causes a premature transcriptional stop and consequently a truncated gene product. These mice were crossed with mice expressing FLP recombinase with a general promotor, which generates offspring that have lost major parts of the inserted cassette, including the premature poly‐A signal, but retain two loxP sites within the introns flanking exon 4. Transgenic Tnfrsf11a‐Cre‐Zc3h12c^fl/fl^ mice were developed by cross breeding Tnfrsf11a‐Cre mice^[^ ^42^ ^]^ and homozygous floxed Zc3h12c mice. As described before,^[^ ^42^ ^]^ Tnfrsf11a‐Cre mice were generated by inserting the recombinant Cre gene into the promotor of Tnfrsf11a. The mouse strain was set up with the Zc3h12c deletion in Tnfrsf11a positive cells after crossing with Tnfrsf11a‐Cre mice. Littermate controls without the Tnfrsf11a‐Cre but homozygous for the floxed Zc3h12c gene were considered as wildtype (WT), and those with heterozygous Tnfrsf11a‐Cre and the homozygous for the floxed Zc3h12c were considered as Zc3h12c conditional knockout (Tnfrsf11a‐Zc3h12c^cKO^).

### Animal Studies

All mice were housed under specific‐pathogen‐free conditions under a 12‐h light and dark cycle with a temperature of 22 ± 2 °C. All necessary materials including mice cages, diet, drinking water, and nesting materials, were thoroughly sterilized by autoclaving prior to use. During the experiments, mice had access to a standard chow diet (Ssniff, Soest, Germany) and to water ad libitum. Two models of unilateral renal ischemia‐reperfusion injury (uIRI)^[^ ^43^ ^]^were employed: *20 min uIRI without contralateral nephrectomy (uNX)*, which simulates a state of persistent or sustained ischemia, as the healthy contralateral kidney maintains systemic renal function (Supplementary materials, Table S1, Supporting Information); and *16 min uIRI with uNX*, which leads to increased hemodynamic load and greater blood perfusion to the injured kidney. For the *Calcium oxalate (CaOx) model*, mice were housed in filter‐top cages, with unrestricted access to food and water. The mice were fed the oxalate‐rich diet (Ssniff, Soest, Germany) to produce nephrocalcinosis‐related kidney injury, which involved supplementing the calcium‐free standard diet with 50 µmol g^−1^ of sodium oxalate, as previously described.^[^ ^44^, ^45^ ^]^ All animal procedures, including euthanasia and organ collection for subsequent analyses (Supplementary materials, Table S2–S5, Supporting Information), were conducted in strict accordance with the ARRIVE guidelines,^[^ ^46^, ^47^ ^]^ German animal care legislation and the EU Directive 2010/63/EU as approved by the local government authority (Regierung von Oberbayern)., i.e., the Ethical Committee of the Regierung von Oberbayern, permit no. ROB‐55.2‐2532.Vet_02‐20‐101.

### Flow Cytometry–Kidney Mononuclear Cell Preparation

The mouse kidney was processed by removing its capsule and smashing it onto a plate containing a solution of 2 mL DMEM mixed with 40 U DNase I and 1.5 mg mL^−1^ collagenase A, then was digested at 37 °C while being agitated 45‐min at 120 rpm. The homogenization of the kidney was achieved by squeezing and passing it through a 70 µm filter. The homogenate was subjected to centrifugation for 5 min at 1,200 rpm in 4 °C, and the resulting pellets were rinsed with fluorescence‐activated cell sorting (FACS) buffer (0.1% NaN3, 1% FCS, 500 ml PBS). The sample was then loaded with 40% Percoll and gently overlaid with 70% Percoll before being centrifuged for 30 min with brake‐off. Collect the cells from the interface layer and wash them with FACS buffer, and then centrifuged. In order to further purify leukocytes, the sample was subjected to magnetic bead‐based depletion using CD45 microbeads (Miltenyibiotec, Germany) and LD columns (Miltenyibiotec, Germany). *Cell staining*: Following the washing step with the FACS buffer, the sample underwent treatment with an Fc blocker and was then exposed to an antibody conjugated with a fluorochrome, as specified in Table S6 (Supporting Information), for a duration of 30 min in the absence of light at room temperature. After this step, the sample underwent another washing phase with FACS buffer, followed by centrifugation. Resuspend the pellet in FACS buffer in preparation for flow cytometry analysis.

### High‐Throughput Transcriptome Sequencing–Single Cell RNA‐seq (scRNA‐seq)

The raw data or count matrices were obtained from GSE174324,^[^ ^48^ ^]^ GSE200115,^[^ ^49^ ^]^ and Kidney Precision Medicine Project.^[^ ^50^ ^]^ The scRNA‐seq data were processed using the Scanpy^[^ ^51^ ^]^ pipeline, which facilitated the integration and merging of different batches of data. Quality control was performed by using the threshold values as reported in the original article. The data were normalized using the scran^[^ ^52^ ^]^ package, which included assuming equal size factors, normalizing the library size to counts per million, and log‐transforming the count data. The 3000 most variable genes were used for the principal component analysis. To eliminate technical differences and preserve biological differences, the harmony integration pipeline was employed, which reduces data dimensions and removes batch effects.^[^ ^53^ ^]^ The reduction of data dimensions and Uniform Manifold Approximation and Projection (UMAP) were applied for unsupervised clustering based on the first 50 integrated principal components. The resolution of clustering was determined accordingly. The gene enrichment score was evaluated by “scanpy.tl.score_genes()” functions.^[^ ^54^ ^]^

### RNA‐Sequencing

The library construction and sequencing process was carried out by Beijing Genomics Institute utilizing the DNBSEQ (G400) platform. The RNA samples were subjected to 100‐base‐pair paired‐end sequencing. The bioinformatics workflow, which included data filtering, mapped transcript prediction, analysis of differential gene expression, and gene ontology, was carried out in accordance with the protocols of hisat2,^[^ ^55^ ^]^ samtools,^[^ ^56^ ^]^ FeatureCounts^[^ ^57^ ^]^ /eisaR,^[^ ^58^ ^]^ clusterProfiler.^[^ ^59^ ^]^ BayesPrism was conducted by following the original paper's pipeline^[^ ^60^ ^]^ based on the scRNA‐seq dataset from GSE174324.^[^ ^48^ ^]^

### In Vitro RNA Degradation Assay

STAT1 alternative splicing variants (STAT1α and STAT1β) were amplified from human cDNA using specific primers and cloned into a T7‐driven transcription vector. In vitro, transcription was performed using the MEGAscript T7 Transcription Kit (Thermo Fisher Scientific) according to the manufacturer's protocol. RNA products were treated with 1 U DNase I (Thermo Fisher Scientific) at 37 °C for 15 min, followed by inactivation with 5 mm EDTA. The RNA was purified using the RNeasy Mini Kit (Qiagen) with an additional 70% ethanol wash step and quantified using Nanodrop 2000 (Thermo Fisher Scientific for accuracy (A260/A280 > 2.0; A260/A230 > 2.2). RNA integrity was assessed by denaturing 1.5% agarose gel electrophoresis. Purified RNA was aliquoted and stored at −80 °C to avoid freeze‐thaw cycles. Recombinant human ZC3H12C WT was purchased from Euprotein. The proteins were stored at −80 °C in the manufacturer‐supplied storage buffer and thawed on ice before use.

In vitro degradation assays were performed in 10 µL reactions containing 200 ng STAT1α/β RNA with 1 µg ZC3H12C protein in 5 µL reaction buffer (20 mm Tris‐HCl [pH 8.0], 100 mm KCl, 1 mm MgCl_2_, 1 mm DTT, 25 µm ZnSO_4_, 0.2 U µL^−1^ RNasin). After incubation at 37 °C (thermal cycler) for 0–20 min, reactions were terminated with 2× RNA loading dye (NEB) at 95 °C for 2 min. Samples were resolved on 6% denaturing urea‐PAGE (120 V, 45 min in 1× TBE) and stained with SYBR Gold (Thermo Fisher Scientific). Three technical replicates were quantified using ImageJ after normalizing to no‐protein controls.

### Statistics

For pairwise comparison, continuous variables were subjected to *t*‐test for normally distributed variables or the Mann–Whitney U test for non‐normally distributed, and categorical variables were subjected to χ2 test or Fisher's exact test. For multiple comparisons, continuous variables were subjected to two‐way analysis of variance followed by Tukey Post‐hoc‐Test. The restricted cubic splines were used for data interpolation and curve fitting. All data are presented as mean ± standard deviation (SD). A P‐value lower than 0.05 was considered to indicate statistical significance. Python or R was used for all figures preparation and statistical analyses.

## Conflict of Interest

The authors declare no conflict of interest.

## Author Contributions

C.L. designed and conducted both in vivo and in vitro experiments, performed data analysis, and prepared the manuscript. J.A.M. contributed to the experimental strategy. Y.K. conducted in vivo and in vitro experiments. Z.Z. carried out in vivo experiments and performed data analysis. S.S. designed and conducted FACS experiments and analyzed the resulting data. N.Z. and Y.X. conducted in vivo experiments and performed immunofluorescence staining. X.L. and H.D. conducted RNA decay experiments. M.L. contributed to the design of in vivo and in vitro experiments. C.S. developed the study concept. H.J.A. contributed to the study concept, experimental design, data analysis, secured funding, and prepared the manuscript. All authors reviewed, edited, and approved the final version of the manuscript.

## Supporting information



Supporting Information

## Data Availability

The data that support the findings of this study are openly available in GEO at https://www.ncbi.nlm.nih.gov/geo/, reference number 236259.
