# Supplementary material for: Macrophage Zc3h12c Limits Tissue Inflammation and Injury via Alternative Splicing of Pre‐mRNA
Source: Adv Sci (Weinh). 2025 Aug 20;12(40):e06707. doi: 10.1002/advs.202506707 (PMC12561471; doi:10.1002/advs.202506707)
Supplement: Supplementary file 1 — Supporting Information [file ADVS-12-e06707-s001.docx]

**Supplementary materials for “****Macrophage** **Zc3h12c limits tissue inflammation and injury via alternative splicing of pre-mRNA”**

Chenyu Li^1, 2*^, Julian Aurelio Marschner^1, 3^, Yoshihiro Kusunoki^1, 4^, Ningxin Zhang^2^, Xiaoxin Li^1^, Hao Deng^1^, Zhibo Zhao^5^, Kanako Watanabe-Kusunoki^1, 4^, Zhihui Zhu^1^, Yan Xu^2^, Stefanie Steiger^1^, Maciej Lech^1^, Katalin Susztak^6, 7, 8^, Christian Schulz^9, 10, 11^, Hans-Joachim Anders^1*^

1. Division of Nephrology, Department of Medicine IV, Hospital of the Ludwig-Maximilians-University, Munich, Germany
2. Department of Nephrology, The Affiliated Hospital of Qingdao University, Qingdao, China
3. Department of Pharmacy, Ludwig-Maximilians-Universität München, Munich, Germany.
4. Department of Rheumatology, Endocrinology, and Nephrology, Faculty of Medicine and Graduate School of Medicine, Hokkaido University, Sapporo, Japan
5. Department of Nephrology, Shanxi Provincial People's Hospital, The Fifth Clinical Medical College of Shanxi Medical University, Taiyuan, Shanxi, China.
6. Renal, Electrolyte, and Hypertension Division, Department of Medicine, University of Pennsylvania, Perelman School of Medicine, Philadelphia
7. Institute for Diabetes, Obesity, and Metabolism, University of Pennsylvania, Perelman School of Medicine, Philadelphia
8. Department of Genetics, University of Pennsylvania, Perelman School of Medicine, Philadelphia
9. Department of Cardiology, Klinikum der Universität München, Ludwig-Maximilians-Universität, Munich, Germany.
10. DZHK (German Centre for Cardiovascular Research), partner site Munich Heart Alliance, Munich, Germany.
11. Department of Immunopharmacology, Mannheim Institute for Innate Immunoscience (MI3), Medical Faculty Mannheim, Heidelberg University, Mannheim, Germany.

***Correspondence:**

Chenyu Li, Ph.D.

Medizinische Klinik und Poliklinik IV

Klinikum der Universität München

Ziemssenstr. 5

D-80336 München

Email: Chenyu.Li@Pennmedicine.upenn.edu

Hans-Joachim Anders, M.D. (Principal Corresponding Author)

Medizinische Klinik und Poliklinik IV

Klinikum der Universität München

Ziemssenstr. 5

D-80336 München

Email: hjanders@med.uni-muenchen.de

**Table of contents**

[Human kidney samples 4](#_Toc203570964)

[Kidney tissue Proteomics and data processing 4](#_Toc203570965)

[Kidney tissue RNA-seq and data processing 4](#_Toc203570966)

[Measurement of glomerular filtration rate 6](#_Toc203570967)

[Creatinine measurement 6](#_Toc203570968)

[Histology 6](#_Toc203570969)

[RNA isolation and polymerase chain reaction (PCR) 7](#_Toc203570970)

[Bone marrow mononuclear cell (BMDM) isolation 8](#_Toc203570971)

[Tubular cell necrotic soup preparation 8](#_Toc203570972)

[Cell viability and cytotoxicity assay 8](#_Toc203570973)

[Transwell migration assay 9](#_Toc203570974)

[Phagocytosis in monocyte-derived Mφ 9](#_Toc203570975)

[Cell Transfection 10](#_Toc203570976)

[Western Blot Analysis 11](#_Toc203570977)

[Motif Discovery and Alternative Splicing Analysis 11](#_Toc203570978)

[Differential Alternative Splicing Analysis 11](#_Toc203570979)

[Reference 12](#_Toc203570980)

[Supplementary Table 1. Receipts for the Narcosis, Antagonist and Analgesia 14](#_Toc203570981)

[Supplementary Table 2. RNA reverse transcription master mix 14](#_Toc203570982)

[Supplementary Table 3. Master mix for the quantitative real-time PCR 14](#_Toc203570983)

[Supplementary Table 4. Primers for the genotype 14](#_Toc203570984)

[Supplementary Table 5. Primers used in this study 15](#_Toc203570985)

[Supplementary Table 6. Antibodies for flow cytometry 15](#_Toc203570986)

[Supplementary Figure 1. Correlation of ZC3H12C and TNFRSF11A Expression with Glomerular Filtration Rate (GFR) and Interstitial Fibrosis. 16](#_Toc203570987)

[Supplementary Figure 2. Cell Type Composition and Tnfrsf11a and Zc3h12c Gene Expression in Human Kidney Tissue using Single-Nucleus and Single-Cell RNA Sequencing. 17](#_Toc203570988)

[Supplementary Figure 3. UMAP plot for sorted immune cells from blood, kidney, and spleen after IRI. 18](#_Toc203570989)

[Supplementary Figure 4. Correlation coefficients heatmap for Tnfrsf11a and Zc3h12c with selected gene set enrichment score. 19](#_Toc203570990)

[Supplementary Figure 5. Dot plot for gene sets enrichment and Tnfrsf11a expression in the kidney mononuclear cell after IRI. 20](#_Toc203570991)

[Supplementary Figure 6. Schematic representation of the targeting strategy for Zc3h12c conditional knockout mice. 21](#_Toc203570992)

[Supplementary Figure 7. Tnfrsf11a and (J-M) Zc3h12a-b mRNA levels in various phagocytes 22](#_Toc203570993)

[Supplementary Figure 8. The qPCR for selected genes in Tnfrsf11a-Zc3h12ccKO 21 days after 20 mins IRI. 23](#_Toc203570994)

[Supplementary Figure 9. The (A) Sirius Red staining and qPCR for kidney 21 days after (B) 16 mins IRI w/o NX and (C) CaOx model. 24](#_Toc203570995)

[Supplementary Figure 10. The qPCR for Kim1, Ngal, Ccl2, Ccr2, Tnf and Tnfrsf11a in kidneys harvested on day 1 after uIRI. 25](#_Toc203570996)

[Supplementary Figure 11. CD45 magnetic beads sorted the immune cell from the blood, bone marrow, and kidney. 26](#_Toc203570997)

[Supplementary Figure 12. The kidney pro-inflammatory and anti-inflammatory Mφ in of Tnfrsf11a-Zc3h12ccKO mice one day after IRI. 27](#_Toc203570998)

[Supplementary Figure 13. Tnfrsf11a and Zc3h12 family member mRNA expression levels in pro-inflammatory or anti-inflammatory polarized Mφ in vitro. 28](#_Toc203570999)

[Supplementary Figure 14. Bulk RNA-seq on Zc3h12c knockout and wild type naïve pro-inflammatory bone-marrow-derived Mφ and alveolar Mφ. 29](#_Toc203571000)

[Supplementary Figure 15. Visualization on outlier genes and concordance plot for BayesPrism. 30](#_Toc203571001)

[Supplementary Figure 16. Selected enrichment plots from the GSEA analysis. 31](#_Toc203571002)

[Supplementary Figure 17. Zc3h12c-deficiency modulates migration in Mφ. 32](#_Toc203571003)

[Supplementary Figure 18. The mRNA level for genes in pro-inflammatory and anti-inflammatory polarized Mφ. 33](#_Toc203571004)

[Supplementary Figure 19. Selected genes expression on BayesPrism inferred joint cell type fraction in Tnfrsf11a-Zc3h12c^cKD^ naïve and pro-inflammatory Mφ. 34](#_Toc203571005)

[Supplementary Figure 20. Zc3h12c Modulates anti-inflammatory macrophage polarization marker CD206 expression in PMA-differentiated THP-1 macrophages. 35](#_Toc203571006)

[Supplementary Data 36](#_Toc203571007)

Human kidney samples

Kidney tissue samples for this study were procured from surgical nephrectomies. Ensuring that only the normal parts of the tissue, specifically those at least 2 cm from any cancerous lesions, were utilized for analysis. An honest broker de-identified the samples and collected corresponding clinical information such as age, race, gender, and diabetes and hypertension status, in addition to creatinine values. The eGFR was subsequently determined using the latest CKD-EPI equations^1^. The use of these samples and data was approved by the institutional review board of the University of Pennsylvania under the category of "exempt," negating the need for informed consent due to the de-identified nature of the study samples.

Kidney tissue Proteomics and data processing

Kidneys were removed, immediately snap-frozen in liquid nitrogen, and stored at −80°C until homogenization. Tissue was cryopulverized using a CryoMill (Retsch, Haan, Germany), and protein was extracted using a lysis buffer of T-PER tissue protein extraction reagent (Thermo Fisher Scientific, Waltham, MA, USA) containing protease inhibitors (Complete; Roche Diagnostics, Basel, Switzerland). Protein concentrations were measured using bicinchoninic acid (BCA) protein assay (Pierce; Thermo Fisher Scientific). Then, we used the SomaScan assay v4.1 platform (SomaLogic, Boulder, CO, USA) for proteomics, where 7,596 targeted proteins or protein complexes in the dataset were measured. The SomaScan platform technology and its performance characteristics have been previously described^2, 3^. This assay, conducted in a single batch, utilizes slow off-rate modified DNA aptamers (SOMAmers) for binding to specific protein targets with high sensitivity and specificity. It allows for the precise quantification of protein levels in terms of relative fluorescence units, measuring 7,288 human protein analytes. To control for variability across kidney extracts, calibrator and buffer samples were incorporated into a 96-well plate format. Quality control measures were implemented according to the manufacturer's guidelines at both the sample and SOMAmer levels. For samples, hybridization controls were utilized, and for SOMAmers, control aptamers were employed for data normalization and calibration purposes. Initially, sample data underwent normalization to mitigate within-run hybridization variability. This was followed by adaptive normalization, which was executed using a maximum likelihood approach, with point estimates and variance derived from a normal distribution based on a U.S. population reference.

Kidney tissue RNA-seq and data processing

RNA isolation, sequencing and analysis were performed as previously published^4^. Total RNA was isolated from kidney tissue using the RNeasy mini kit (Qiagen, Venlo, Netherlands) according to the manufacturer’s instructions, including the DNase digestion step. RNA quality was assessed by Agilent Bioanalyzer 2100. The cDNA library was prepared using NEBNext Ultra II RNA Library Prep Kit for Illumina. Then, cDNA libraries were sequenced on an Illumina NovaSeq 6000 platform using the NovaSeq PE150 protocol. Adaptor and lower quality bases were trimmed with Trim-galore TrimGalore (version 0.4.5). Reads were aligned to the human genome (hg19) using STAR (v2.7.3a). Gene and isoform expression levels of transcript-per-million (TPM) were estimated using RSEM (v1.3.0). We analyzed genes with TPM greater than 0.1 in at least 20% of subjects, resulting in 22,053 genes. We selected a subset of genes that correspond to chromosomes 1 through 22, resulting in a total of 21,538 genes being analyzed. We excluded eight individuals from transcriptomic analysis based on PCA outlier detection, where we removed all individuals who deviated by more than three standard deviations from the mean of the first two principal components.

**Unilateral kidney ischemia-reperfusion surgery**

In order to conduct the procedure in an ethical and controlled manner, mice preemptively received the analgesic Metamizol, followed by anesthetization in accordance with the established protocol (Supplementary Table 1)^5^. During the surgical procedure, measures were taken to maintain the core body temperature of the mice within the recommended range of 36.5°C to 38.5°C^5^. An online rectal temperature monitoring system was utilized to continuously monitor the body temperature of the mice until the wound closure process was completed^5^. To maintain mouse core body temperature stable, mice were kept in an incubator set at 37°C before and after surgery. Additionally, the surgical procedure was performed on an operating table maintained at a constant temperature of 41°C to further ensure a constant core body temperature. The experimental group underwent NX of the right kidney, followed by one week later, left renal pedicle clamping for 16 minutes using a microaneurysm clamp (Medicon, Tuttlingen, Germany). To avoid dehydration or contraction of the uncovered kidney, two drops of physiological saline at a temperature of 37°C were administered to all mice. After removal of the clamp, the kidney recovery was confirmed through observation of blood reperfusion and the return of the kidney to its original color. Then, the muscular layer was sutured using Vicryl while Ethibond was used for the cutaneous layer for the wound closure process. Buprenorphine (0.1mg/kg) was given as analgesia 30 minutes before ending the anesthesia. The same operation was conducted on sham group mice, except for the renal pedicle clamping step.

Measurement of glomerular filtration rate

We measured glomerular filtration rate (GFR) by assessing sinistrin clearance using a transdermal registration method according to the protocol^6^. A sinistrin compound conjugated with fluorescein-isothiocyanate (FITC) was utilized as an exogenous GFR tracer to determine the excretion kinetics. The FITC-sinistrin solution was prepared in 30 mg/ml in physiological saline. The mice were first anesthetized by 20% oxygen isoflurane before the GFR device (Medibeacon, Mannheim, Germany) with the battery was mounted to the shaved neck of the mice via a patch (Medibeacon), with one side of the patch attaching to the GFR device with the battery and the other side attaching to the mice. The device was then secured in place with hypoallergenic silk tape. The device begins recording data after a light-emitting diode flashes. To acquire the baseline signal, the device was left running for approximately 10 minutes. Subsequently, the FITC-sinistrin was administered to the mice via intravenous injection at a dose of 0.15 mg/g. The measurement period is approximately 1.5 hours. The GFR device data were read and processed using the MPD Lab software (MediBeacon).

Creatinine measurement

Before sacrifice, a blood sample was obtained from the retro-orbital plexus under 20% oxygen isoflurane anesthesia. The sample was collected in 1.5 ml tubes with heparin and centrifuged for 10 minutes at 800 rounds per minute. Plasma supernatant was collected and stored at -80°C. To measure creatinine levels in plasma samples, a standardized protocol was implemented utilizing a commercial creatinine assay kit. Standard solutions were created with varying concentrations to establish a standard curve for reference. The working reagent mix was prepared according to the instructions of kit. A 10 µl plasma aliquot was placed onto the 96-well plate and mixed with the reagent immediately prior to measurement. The absorbance was then read at 492 nm at specific time intervals (60, 120, 180 seconds, and 20 minutes) after the plasma-reagent mixture was formed. The adjusted absorbance was normalized by subtracting the blank reading. The final creatinine concentration in the plasma sample was then fitted by applying the adjusted absorbance value to the standard curve.

Histology

*Periodic Acid Schiff staining.* The sections that had been rehydrated were initially treated with 2% periodic acid for 5 minutes and then rinsed with double-distilled water. Subsequently, they were stained with for 20 minutes Schiff solution and rinsed with tap water. Hematoxylin solution was used to counterstain the sections for 2 minutes and then washed with tap water. After drying, the sections were dipped in 90% alcohol and covered with slips. The injury extent of tubular was measured by assessing the corticomedullary junction proportion that demonstrated indications of brush border loss, tubular dilatation and cast formation. A scoring system ranging from 0 to 2 was used to quantify the severity of injury, with 0 indicating no injury and 2 indicating 100% injury. The overall PAS score was graded on a scale of 0 to 8.

*Sirius red staining.* The Weigert's hematoxylin was first used to stain the rehydrated sections for 8 minutes and then washed with double-distilled water. Picro-sirius red was applied for 60 minutes, and then rinse the sections with tap water. Following that, the sections were dipped in 90% ethanol and covered with slips after drying. The extent of kidney fibrosis was determined by calculating the percentage of collagen (Red) in the tissue sections using python and OpenCV^7^.

RNA isolation and polymerase chain reaction (PCR)

The tissue samples were carefully and delicately transferred to a container with 2 ml of lysis buffer, which contains 1 % 2-mercaptoethanol, using forceps. The samples were always kept on the ice to ensure preservation of the RNA integrity. The samples were homogenized for 20 seconds at a level 4 setting on the Ultra-Turrax machine. The homogenized samples were then centrifuged at 6,000g for 5 minutes to separate the supernatant from the pellet. The supernatant was saved and transferred to a new, RNase-free tube. Subsequently, 700 µl of the supernatant were added to an equal amount of 70% ethanol, and the mixture was thoroughly mixed. The rest of the RNA isolation procedure was carried out by following the Qiagen mRNA extraction kit instructions. The RNA samples were subsequently put in storage at -80°C for long-term preservation.

The purified RNA was subjected to a denaturation process by incubating it at 65°C for 10 minutes to breakdown the secondary structures of the RNA. This reaction was subsequently halted by placing the RNA at 4°C. Each sample’s RNA was prepared at a final concentration of 2 µg in 22.45 µl of a pre-prepared master mix (as outlined in Supplementary Table 3). The resulting mixture was then kept at 42°C for 2 hours followed by an 85°C for 5 minutes to generate cDNA. The cDNA was then deposited at -20°C for future use.

The SYBR-Green was utilized as fluorescent dye for qPCR. The cDNA samples were first diluted at a ratio of 1:100 and then mixed with SYBR-Green, Taq polymerase (Supplementary Table 4), and primers (as outlined in the Supplementary Table 5, 6). The mixture was run on a Light Cycler 480 instrument with the following setting: 5-minute pre-incubation at 95°C, 15-second amplification at 95°C, 60°C for 15-second, and 68°C for 20-second, with 40 cycles total. A melting curve as also performed at 95°C for 5-second, 65°C for 60-second. The samples were then cooled to 40°C for 30-second. The cycle threshold values were analyzed using the Light Cycler 480 instrument. For statistical analysis and plotting, the mRNA relative expression was determined using the cycle threshold values normalized by 18s rRNA.

Bone marrow mononuclear cell (BMDM) isolation

After obtaining femurs and tibiae from mice -sacrificed mice by cervical dislocation, the bones were immersed in 75% ethanol for 1 minute before being stored in PBS. Removing the residual tissue from the bones, and the tibiae were separated from the femurs by gently breaking the knee joint. Then removing the terminal caps of the bones by sterile scissors and putting marrow cavity into the cavity to flush the bone marrow with PBS until the bone appeared white. The bone marrow was centrifuged 5-minute at 1,200 rpm for. Then 0.155 M NH4Cl was used to resuspend the pellet to lyse erythrocytes for 10 minutes on ice, passed through a 70 µM strainer, and centrifuged 5-minute at 1,200 rpm. L929-conditioned Dulbecco's Modified Eagle Medium (DMEM) was used to suspend the pellet was and cells were grown in culture dishes in an incubator with 37°C, 5% CO2, and humidity control. Every 3 days, we added fresh medium to the dishes, and harvested the cells after 7 days for further experiments.

Tubular cell necrotic soup preparation

The tubular cell necrotic soup was obtained by harvesting 10 million primary murine TEC in 1 ml PBS, which was then subjected to a freezing-thawing process repeated five times. The samples were then centrifuged 5-minute at 500g, and the supernatant was collected as the necrotic protein solution for stimulation. The necrotic protein solution was mixed to the medium and incubated with cells for one day in a ratio of 1:1 for necrotic tubular TEC to stimulated cells.

Cell viability and cytotoxicity assay

*Cell viability activity:* The cells were cultivated on 96-well plate, with each well containing 1x10^4^ cells, and a 100 µl volume of cell culture medium with a 2.5% FCS concentration. The cells were allowed to adhere overnight and prior to stimulation, all medium was replaced. The positive group was subjected to 2% Tween stimulation for a duration of 30 minutes. Subsequently, the old medium was aspirated and 100 µl 3- (4, 5-Dimethylthiazol-2-yl)-2, 5-Diphenyltetrazolium Bromide (MTT, CT01, Sigma-Aldrich) was put onto the well and incubated for 4 hours to facilitate the reaction. Afterwards, add 100 µl stop solution onto well and the plate was stored overnight in a humid environment. The samples were analyzed using an enzyme-linked immunosorbent assay (ELISA) reader with absorbance read at 570 nm. The reference wave length was required to be greater than 650 nm.

*Cell cytotoxicity:* The cells were seeded onto the 96-well plate at 1x10^4^ cell/well in 100 µl 2.5% FCS medium and left overnight for the cell adhesion. Prior to stimulation, all the medium was replaced. The positive control group was subjected to 2% Tween for 30 minutes. After the stimulation, 50 µl supernatant was transferred onto a new plate. According to the protocol (4744934001, Roche), 50 µl of lactate dehydrogenase (LDH) reagent from the kit was put into well, and immediately read the absorbance at 450 nm was using the ELISA reader at 5-minute intervals. The reference wavelength was required to be greater than 650 nm.

Transwell migration assay

The Transwell migration assay was carried out utilizing 8-micrometer pore size inserts (designated as catalog CLS3464-48EA, supplied by Corning® Costar® Transwell® cell culture inserts) as previously described in reference^8^. In accordance with sterile conditions, the bone marrow-derived Mφ (BMDMs) were seeded on the transwell insert, with 2×105 cells loaded in 200 μl DMEM, and 700 μl DMEM was put into the bottom well. The plate was placed at 5% CO_2_ and 37°C and for 12 hours prior to the initiation of the assay. The bottom surface of the insert was swabbed to remove cells that had migrated through the membrane. Then, add 10 ng/ml of Ccl2 to the bottom well and further incubate for 12 hours. After incubation, the Transwell inserts were collected, and 4% paraformaldehyde was used to fix the cells for 5 minutes. The inserts were then washed twice with PBS and 0.1% crystal violet (Invitrogen) was used to stain the cell for 15 minutes. Carefully remove the cells with a cotton swab which remaining on the top surface of the e insert membrane. Imaging of the insert membrane was performed using the 10x objective lens at Nikon Eclipse Ti2 microscope.

Phagocytosis in monocyte-derived Mφ

The fluorescent latex beads (green-labeled, 1-µm diameter, L4655 Sigma)^9^ and a latex beads-rabbit IgG-FITC Phagocytosis Assay Kit (catalog number 500290 from Cayman)^10^ were used to quantify phagocytic activity. To quantify phagocytosis, cells were seeded onto the 96-well plate with 1x10^4^ per well and incubated at 5% CO and 37°CO_2_ overnight to allow the cells to adhere. Cytochalasin D (1 µg/mL) was added 45 minutes prior to the bead’s incubation, and the beads were then incubated for further analysis.

*Fluorescent latex beads:* The pre-incubation of latex beads was conducted at a concentration of 50 μg/ml at 37°C for 30 minutes in the conditioned medium. Subsequently, 5 μg/ml latex beads were incubated with cells for 3 hours in a 5% CO_2_ and 37°C incubator. The 96-well plate was then washed with warm PBS for three times. Images were acquired using the Nikon DS-Qi2 camera with both transmitted light and fluorescence. For quantitative analysis, the CellPose^11^ Neural Convolutional Network was employed to detect the cells and create masks for cell location, followed by counting the cell number and calculate the positive area within the cell.

*Latex beads-rabbit lgG-FITC:* In accordance with the instructions provided by the Phagocytosis Assay Kit, the latex beads-rabbit lgG-FITC complex was diluted to a 1:100 with pre-warmed culture medium. The complex was then incubated with cells for a period of 3 hours in a controlled 5% CO_2_ and 37°C environment. The 96-well plate was subsequently rinsed three times with warm PBS. In order to differentiate between cells that have internalized the beads and those with beads attached to their surface, the cells were subjected to the trypan blue quenching solution incubation for 2 minutes and washing with FACS buffer. Then, the cells were detached from the plate and resuspended in FACS buffer, ready to undergo flow cytometry analysis^12^.

Cell Transfection

The THP-1 cells were cultured in specific complete medium, containing RPMI-1640, 10% FBS, 0.05mM β-mercaptoethanol and 1% P/S and were seeded in TC-treated 6-well plates. PMA (100 ng/mL) was added to THP-1 cells to induce adherence. Transfection was performed after 24 hours, when complete adhesion was achieved. The Zc3h12c overexpression plasmid was constructed using the pcDNA3.1(+) vector, with an empty pcDNA3.1(+) plasmid serving as a control. Small interfering RNA (siRNA) targeting Zc3h12c (sense: GCAAAGUUACAGUCACGAATT; antisense: UUCGUGACUGUAACUUUGCTT) and a negative control siRNA (sense: UUCUCCGAACGUGUCACGUTT; antisense: ACGUGACACGUUCGGAGAATT) were synthesized. Plasmid and siRNA transfections were performed using a liposome-based transfection method as follows: 200 μL of serum-free medium was added to an EP tube, followed by the addition of either 5 μg Zc3h12c overexpression plasmid, 5 μg empty pcDNA3.1(+) plasmid, Zc3h12c-siRNA, or negative control siRNA. For tubes containing the overexpression plasmid or empty pcDNA3.1(+) plasmid, 6 μL of P3000 reagent was added. Another EP tube was prepared with 200 μL of serum-free medium and 5 μL of Lipofectamine 3000. The contents of both tubes were mixed, incubated at room temperature, and then added to the wells of the 6-well plate, which had been refreshed with new medium. Cells were incubated for 24 hours before subsequent polarization induction.

Western Blot Analysis

THP-1 cells were lysed in 100 μL RIPA buffer per well to extract total protein. Protein concentration was determined using the BCA assay. Equal amounts of protein (30 μg per lane) were subjected to SDS-PAGE and subsequently transferred onto PVDF membranes. Membranes were blocked with 3% BSA and incubated overnight at 4°C with primary antibodies, including β-actin (1:1000, Elabscience, #AN005290L), STAT1 (1:1000, CST, #14994S), Phospho-STAT1 (Tyr701) (1:1000, CST, #9167S), Phospho-STAT1 (Ser727) (1:1000, CST, #8826S), and ZC3H12C (1:1000, Novus, #NBP2-82064). Membranes were then incubated with HRP-conjugated secondary antibodies at room temperature for 1 hour. Protein bands were visualized using a chemiluminescence imaging system (Uvitec Alliance Q9), and grayscale intensity was analyzed using ImageJ software.

Motif Discovery and Alternative Splicing Analysis

The RNA-binding motif for ZC3H12C was obtained from the high-resolution specificity models published in Jolma et al.^13^ The position weight matrix (PWM) for ZC3H12C was converted into MEME Suite-compatible format for FIMO analysis^14^. To identify potential regulatory motifs, we employed FIMO (Find Individual Motif Occurrences), a tool within the MEME Suite (version 5.4.0)^15^.

Differential Alternative Splicing Analysis

To investigate differential alternative splicing events between experimental conditions, we utilized rMATS^16^ (version 4.1.2, Replicate Multivariate Analysis of Transcript Splicing). Input to rMATS consisted of paired-end RNA-Seq reads aligned to the hg38 genome using STAR v.2.7.5b.^17^ rMATS was run using default parameters to compare control versus treatment groups. rMATS identifies and quantifies various types of alternative splicing events, including exon skipping, intron retention, alternative 5' splice sites, alternative 3' splice sites, and mutually exclusive exons. Significant differential splicing events were defined based on a false discovery rate (FDR) adjusted p-value of < 0.05 and a minimum absolute difference in percent spliced in (ΔPSI) of ≥ 0.1. The rMATS output provided detailed information on differentially spliced events, including event type, genomic location, and statistical significance.

Reference

1. Inker LA, Eneanya ND, Coresh J, et al. New Creatinine- and Cystatin C-Based Equations to Estimate GFR without Race. N Engl J Med. 2021; 385: 1737-49.

2. Hirohama D, Abedini A, Moon S, et al. Unbiased Human Kidney Tissue Proteomics Identifies Matrix Metalloproteinase 7 as a Kidney Disease Biomarker. J Am Soc Nephrol. 2023; 34: 1279-91.

3. Niewczas MA, Pavkov ME, Skupien J, et al. A signature of circulating inflammatory proteins and development of end-stage renal disease in diabetes. Nat Med. 2019; 25: 805-13.

4. Sheng X, Guan Y, Ma Z, et al. Mapping the genetic architecture of human traits to cell types in the kidney identifies mechanisms of disease and potential treatments. Nat Genet. 2021; 53: 1322-33.

5. Marschner JA, Schafer H, Holderied A and Anders HJ. Optimizing Mouse Surgery with Online Rectal Temperature Monitoring and Preoperative Heat Supply. Effects on Post-Ischemic Acute Kidney Injury. PLoS One. 2016; 11: e0149489.

6. Scarfe L, Schock-Kusch D, Ressel L, et al. Transdermal Measurement of Glomerular Filtration Rate in Mice. J Vis Exp. 2018.

7. Bradski G. The OpenCV Library. Dr Dobb's Journal of Software Tools. 2000.

8. Berzaghi R, Ahktar MA, Islam A, Pedersen BD, Hellevik T and Martinez-Zubiaurre I. Fibroblast-Mediated Immunoregulation of Macrophage Function Is Maintained after Irradiation. Cancers (Basel). 2019; 11.

9. Triboulet S, Aude-Garcia C, Carriere M, et al. Molecular responses of mouse macrophages to copper and copper oxide nanoparticles inferred from proteomic analyses. Mol Cell Proteomics. 2013; 12: 3108-22.

10. Pireaux V, Sauvage A, Bihin B, et al. Myeloperoxidase-Oxidized LDLs Enhance an Anti-Inflammatory M2 and Antioxidant Phenotype in Murine Macrophages. Mediators Inflamm. 2016; 2016: 8249476.

11. Stringer C, Wang T, Michaelos M and Pachitariu M. Cellpose: a generalist algorithm for cellular segmentation. Nat Methods. 2021; 18: 100-6.

12. Jaggi U, Yang M, Matundan HH, et al. Increased phagocytosis in the presence of enhanced M2-like macrophage responses correlates with increased primary and latent HSV-1 infection. PLoS Pathog. 2020; 16: e1008971.

13. Jolma A, Zhang J, Mondragon E, et al. Binding specificities of human RNA-binding proteins toward structured and linear RNA sequences. Genome Res. 2020; 30: 962-73.

14. Grant CE, Bailey TL and Noble WS. FIMO: scanning for occurrences of a given motif. Bioinformatics. 2011; 27: 1017-8.

15. Bailey TL, Johnson J, Grant CE and Noble WS. The MEME Suite. Nucleic Acids Res. 2015; 43: W39-49.

16. Shen S, Park JW, Lu ZX, et al. rMATS: robust and flexible detection of differential alternative splicing from replicate RNA-Seq data. Proc Natl Acad Sci U S A. 2014; 111: E5593-601.

17. Dobin A, Davis CA, Schlesinger F, et al. STAR: ultrafast universal RNA-seq aligner. Bioinformatics. 2013; 29: 15-21.

Supplementary Table 1. Receipts for the Narcosis, Antagonist and Analgesia

| **Application** | **Drug** | **Administration Method** | **Conc. (mg/kg)** | **Cat.** | **Company** |
| --- | --- | --- | --- | --- | --- |
| Narcosis | Medetomidine | i.p. once prior to surgery | 0.5 | 7725752 | Zoetis |
|  | Midazolam |  | 5 | 4921530 | Ratiopharm |
|  | Fentanyl |  | 0.05 | 2084366 | Janssen-Cilag |
| Antagonist | Atipamezole | s.c. once after the surgery | 5 | 8-00732 | CP-Pharma |
|  | Flumazenil |  | 0.1 | 4470990 | Hexal |
| Analgesia | Buprenorphine | i.p. 30 min before antagonization,  t.i.d. after the surgery for 3 days | 0.1 | 1498870 | Bayer Vital |
|  | Metamizole | p.o. 5min before narcosis induction | 200 | 731672 | Sanofi-Aventis |

* i.p., Intraperitoneal injection; s.c., subcutaneous; p.o. oral administration

Supplementary Table 2. RNA reverse transcription master mix

| **Master mix** | **Concentration** | **Volume (µl)** |
| --- | --- | --- |
| Taq buffer | 5x | 4.5 |
| DTT | 0.1M | 1 |
| dNTPs | 25 mM | 0.45 |
| Rnasin and ribonuclease inhibitor | 40 u/µl | 0.5 |
| Acrylamide | 15 µg/ml | 0.25 |
| Hexanucleotide Mix | 10x | 0.25 |
| Superscript II | 200 u/µl | 0.5 |
| Sample | 2 µg | 15 |
| Total |  | 22.45 |

Supplementary Table 3. Master mix for the quantitative real-time PCR

| **Master mix** | **Concentration** | **Volume (µl)** |
| --- | --- | --- |
| Mix SybrGreen |  | 10 |
| Taq polymerase | 5000 u/ml | 0.16 |
| Reverse primer | 10 µM | 0.6 |
| Forward primer | 10 µM | 0.6 |
| cDNA |  | 0.2 |
| ddH2O |  | 8.44 |
| Total |  | 20 |

Supplementary Table 4. Primers for the genotype

| **Primers** | **Sequence** | **Product** |
| --- | --- | --- |
| **Tnfrsf11a Cre** |  |  |
| Tnfrsf11a-ORF#1618_fw | TCAAGGGTGACATCATCGTGGT | 506 bp |
| Tnfrsf11a-Cre_PGKNeo_fw | GTAACTTCTCCATGGTAGCCTC | 251 bp |
| Tnfrsf11a-ORF#2321_rev | CTCAATAATGCAGGACACCAACG | |
| **Zc3h12cflox** |  | 303 bp |
| gZc3h12c_WT+KO_#3_F | GGAAGAAGTTCATAGATGAGCGG |  |
| gZc3h12c_KO_#2_R | GAACTGATGGCGAGCTCAGAC | |
| **Zc3h12cwild type** |  | 401 bp |
| gZc3h12c_WT_#1_F | CTGGCTGACAGAAATATCTGTC |  |
| gZc3h12c_WT_#1_R | GGTGCTCAGACTTCAACCT | |

Supplementary Table 5. Primers used in this study

| **Gene** | **Forward** | **Reverse** |
| --- | --- | --- |
| *18s* | GCAATTATTCCCCATGAACG | AGGGCCTCACTAAACCATCC |
| *Αsma* | CCTTCGTGACTACTGCCGAG | ATAGGTGGTTTCGTGGATGC |
| *Fibronectin* | GCCACCATTACTGGTCTGGA | GGTTGGTGATGAAGGGGGTC |
| *Tgfβ* | CAACCCAGGTCCTTCCTAAA | GGAGAGCCCTGGATACCAAC |
| *Kim-1* | TCAGCTCGGGAATGCACAA | TGGTTGCCTTCCGTGTCTCT |
| *Ngal* | ATGTCACCTCCATCCTGG | GCCACTTGCACATTGTAG |
| *Il18* | AGAAAGCCGCCTCAAACCTT | TGTCTGATTCCAGGTCTCCATTT |
| *Inos* | AAACCCCTTGTGCTGTTCTCA | GAACATTCTGTGCTGTCCCAG |
| *Tnf* | AGCCTCTTCTCATTCCTGCT | TAGACAAGGTACAACCCATC |
| *Timp2* | GCAACAGGCGTTTTGCAATG | AGGTCCTTTGAACATCTTTATCTG |
| *Ccl2* | CCTGCTGTTCACAGTTGCC | ATTGGGATCATCTTGCTGGT |
| *Cxcl1* | CCGAAGTCATAGCCACACTCA | CTCCCACACATGTCCTCACC |
| *Cxcl2* | CCCAGACAGAAGTCATAGCCAC | CTTCCGTTGAGGGACAGCAG |
| *Ccr2* | GCTGTGTTTGCCTCTCTACCAG | CAAGTAGAGGCAGGATCAGGCT |
| *Ccr5* | GTCTACTTTCTCTTCTGGACTCC | CCAAGAGTCTCTGTTGCCTGCA |
| *Vcam1* | GCTATGAGGATGGAAGACTCTGG | ACTTGTGCAGCCACCTGAGATC |
| *Icam1* | AAACCAGACCCTGGAACTGCAC | GCCTGGCATTTCAGAGTCTGCT |
| *Il1b* | TGGACCTTCCAGGATGAGGACA | GTTCATCTCGGAGCCTGTAGTG |
| *Il6* | TACCACTTCACAAGTCGGAGGC | CTGCAAGTGCATCATCGTTGTTC |
| *Fpr2* | GCCTTTTGGCTGGTTCCTGTGT | CAAATGCAGCGGTCCAAGGCAA |
| *Cd38* | GGTCCAAGTGATGCTCAATGGG | AGCTCCTTCGATGTCGTGCATC |
| *Arg1* | GTGAAGAACCCACGGTCTGT | ATCGGCCTTTTCTTCCTTCCC |
| *Ym2* | GTGACCCTACTGTTAGTGCTGG | GGTACTTCCTGGGTGGCATCAA |
| *Il1a* | ACGGCTGAGTTTCAGTGAGACC | CACTCTGGTAGGTGTAAGGTGC |
| *Reg1* | AACTGGTTTCTGGAGCGAGG | CGAAGGATGTGCTGGTCTGT |
| *Reg2* | ACCTGCAGAACGAAAACCCA | CATCAGGAGGCATGAACCTGT |
| *Zc3h12c* | GAACAGTCCCGCCCTGAC | CATCATAGCACACCACTCGC |
| *Reg4* | CCTCGTGGGCCCAGCTCCAG | TCCCATGGCTCATTGCCACATTACT |
| *Tnfrsf11a* | GGACAACGGAATCAGTGGTC | CCACAGAGATGAAGAGGAGCAG |
| *Il1ra* | CTGTTGGTGAGGAATGTGGCTG | GGCTCAGGATAACAGGTCTGTC |

Supplementary Table 6. Antibodies for flow cytometry

| **Antibody** | **Clone** | **Fluorescent dyes** | **Company** |
| --- | --- | --- | --- |
| anti-mouse F4/80 | BM8 | APC | Biolegend |
| anti-mouse CD192 (CCR2) | SA203G11 | FITC |  |
| anti-mouse CD206 (MMR) | C068C2 | FITC |  |
| anti-mouse Ly-6C | HK1.4 | PerCP/Cyanine5.5 |  |
| anti-mouse CD86 | GL-1 | PerCP |  |
| anti-mouse/human CD11b | M1/70 | PE/Cyanine7 |  |


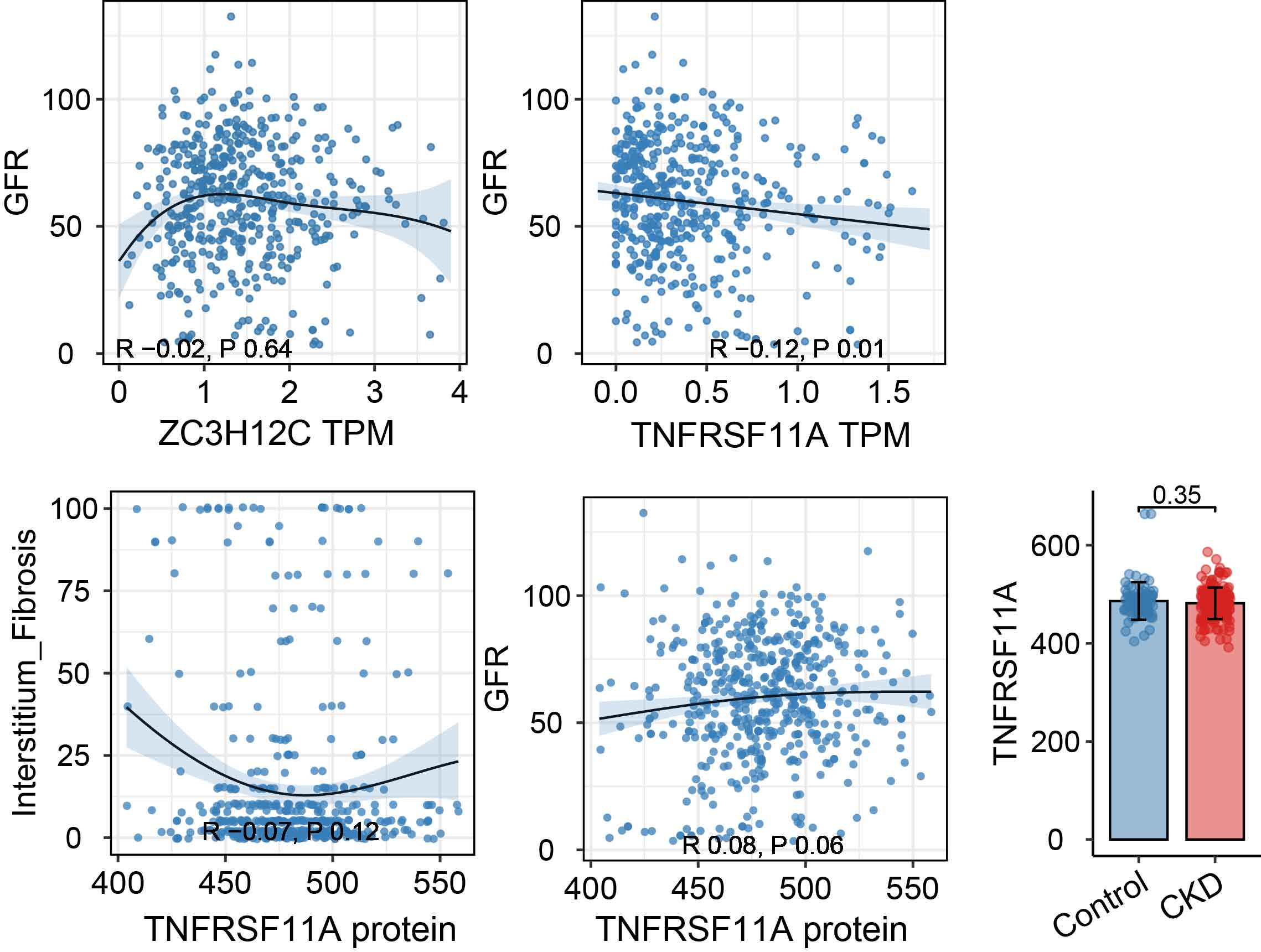


Supplementary Figure 1. Correlation of ZC3H12C and TNFRSF11A Expression with Glomerular Filtration Rate (GFR) and Interstitial Fibrosis.

Top panel showed scatter plots which illustrating the relationship between gene expression or protein levels and clinical parameters in human kidney tissue samples. Correlation between ZC3H12C and TNFRSF11A transcript per million (TPM) from RNA-sequencing data and estimated GFR (eGFR). Bottom panel showed correlation between TNFRSF11A protein levels measured by SomaScan assay and percentage of interstitial fibrosis and eGFR. Correlation coefficients (R) and p-values (P) are indicated in each plot, derived from Pearson correlation analysis. Bar plot comparing TNFRSF11A protein levels, measured by SomaScan assay, in control kidney samples and samples from patients with CKD. P-value indicated above the plot is from a t-test. The eGFR was calculated using CKD-EPI equations. Proteomic data was generated using the SomaScan assay platform, quantifying protein levels in relative fluorescence units. All quantitative data are means ± SD (n = 516). Two-way ANOVA or t-test was employed for the statistical examination.


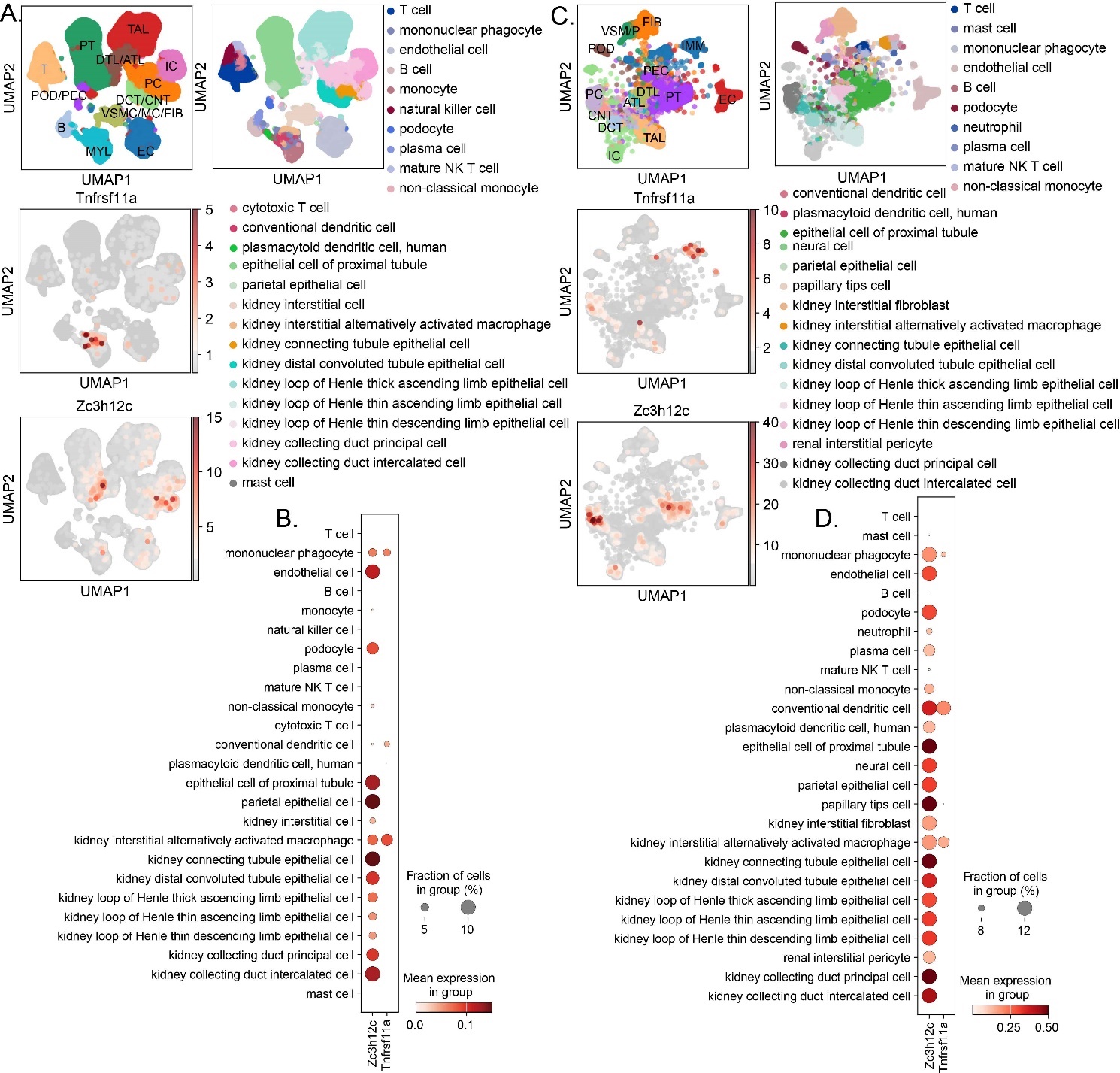


Supplementary Figure 2. Cell Type Composition and Tnfrsf11a and Zc3h12c Gene Expression in Human Kidney Tissue using Single-Nucleus and Single-Cell RNA Sequencing.

Uniform Manifold Approximation and Projection (UMAP) visualization of cell clusters identified from single-nucleus RNA sequencing (snRNA-seq) (A, B) and single-cell RNA sequencing (scRNA-seq) (C, D) of human kidney tissue from the Kidney Precision Medicine Project (KPMP). Each point represents a single nucleus (A) or cell (C), colored by cell type as indicated in the legend. Abbreviations in (A) and (C) represent: POD/PEC (podocyte/parietal epithelial cell), B (B cell), MYL (myeloid lineage), PT (proximal tubule), TAL (thick ascending limb), DTL/ATL (descending thin limb/ascending thin limb), IC (intercalated cell), PC (principal cell), DCT/CNT (distal convoluted tubule/connecting tubule), VSMC/MC/FIB (vascular smooth muscle cell/mesangial cell/fibroblast), EC (endothelial cell). (B, D) Dot plots showing the fraction of cells expressing and the mean expression level of Tnfrsf11a and Zc3h12c genes within each cell type for snRNA-seq (B) and scRNA-seq (D) datasets. Dot size represents the percentage of cells in each cell type expressing the gene, and dot color intensity indicates the average expression level within that cell type. The color scales for gene expression and dot sizes are shown below each dot plot. The figure illustrates the cell type composition and gene expression patterns in human kidney tissue as profiled by snRNA-seq and scRNA-seq, highlighting the expression of Tnfrsf11a and Zc3h12c across different kidney cell populations.


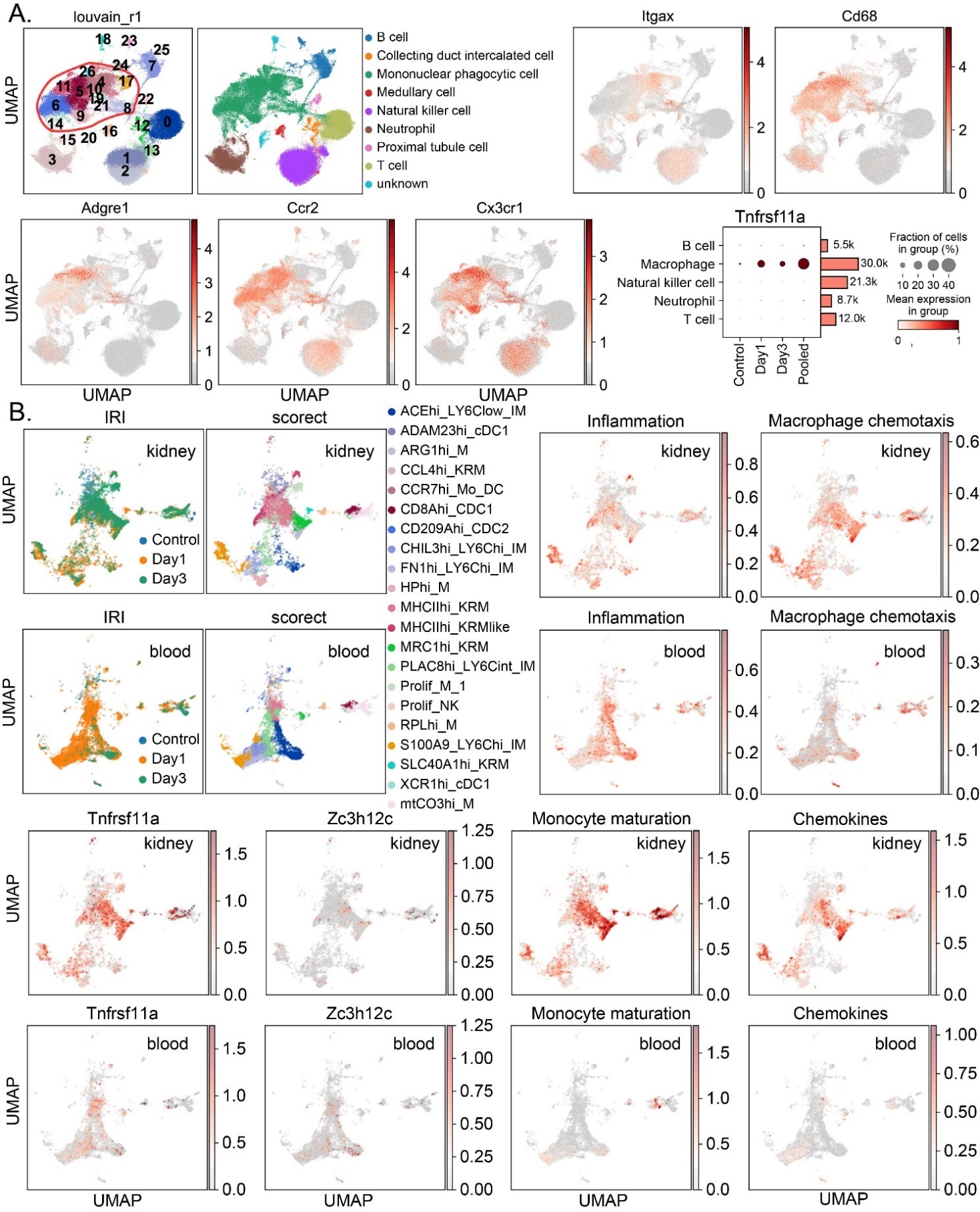


## **Supplementary Figure 3. UMAP plot for sorted immune cells from blood, kidney, and spleen after IRI.**

(A) All cells from blood, kidney, and spleen. the clusters were segmented by the Louvain algorithm with the resolution of 1. According to feature plots of markers (Cd68, Ccr2, Adgre1, Cx3cr1, Itgax and Cd209a), the clusters in the red circle represent different subsets of Mφ. (B) Mφ from blood, and kidney. The cells were annotated based on cell markers from ImmGen mouse immune cell datasets. The activation of genes important for phagocytosis, inflammation, chemotaxis, trafficking, and monocyte maturation were assessed based on the average expression of corresponding function. IM, infiltrating monocytes; DC, dendritic cells; M, Monocyte; KRM, kidney resident Mφ; Mo, Mφ; NK, natural killer cell. (Adopted from GSE174324)


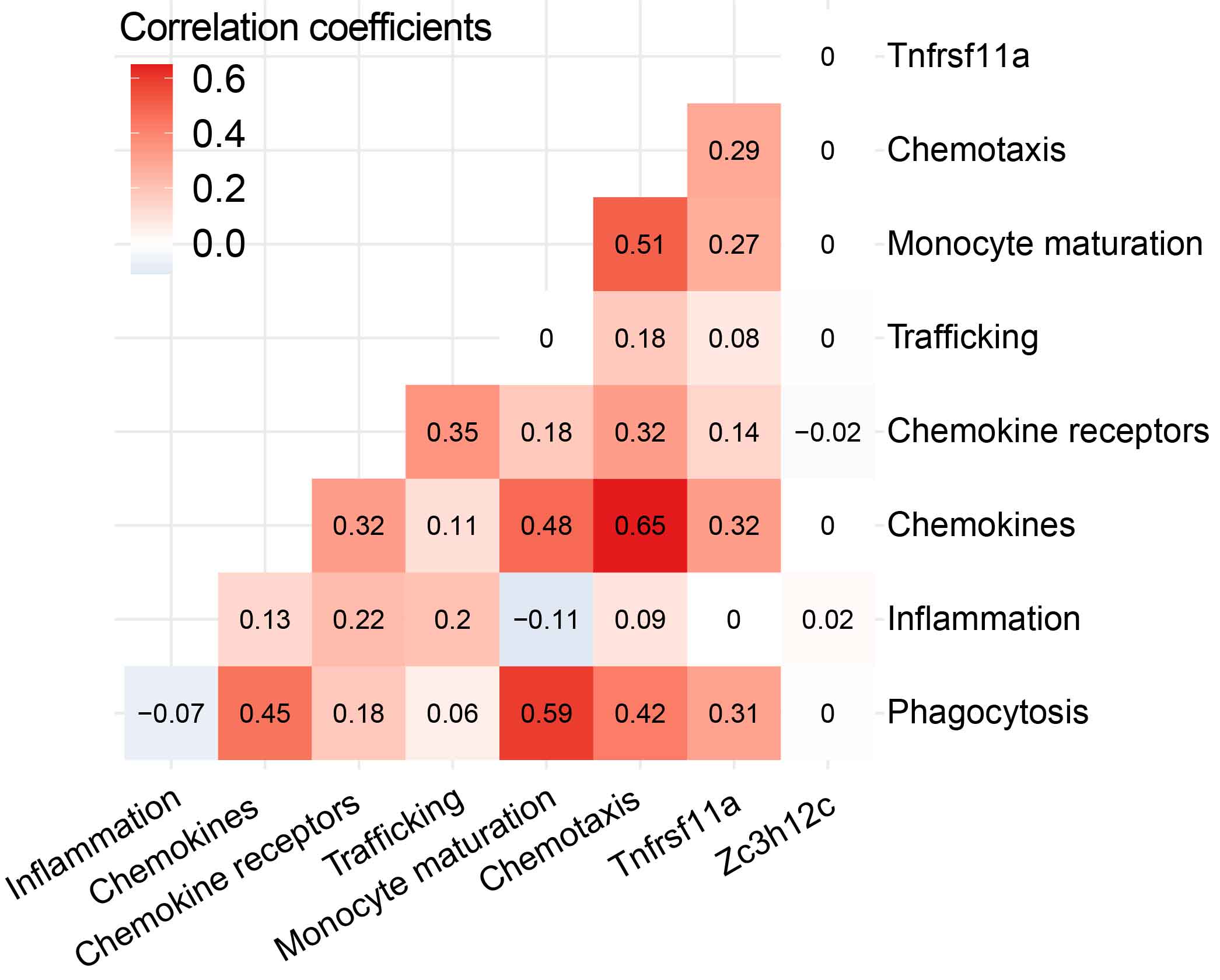


## Supplementary Figure 4. Correlation coefficients heatmap for Tnfrsf11a and Zc3h12c with selected gene set enrichment score.

Heatmap displaying Pearson correlation coefficients between Tnfrsf11a or Zc3h12c mRNA expression and gene set enrichment scores sorted kidney macrophages from the unilateral ischemia-reperfusion injury model. Data derived from single-cell RNA sequencing (scRNA-seq) of macrophage cells (Adopted from GSE174324). Color scale: Red (positive correlation), blue (negative correlation).


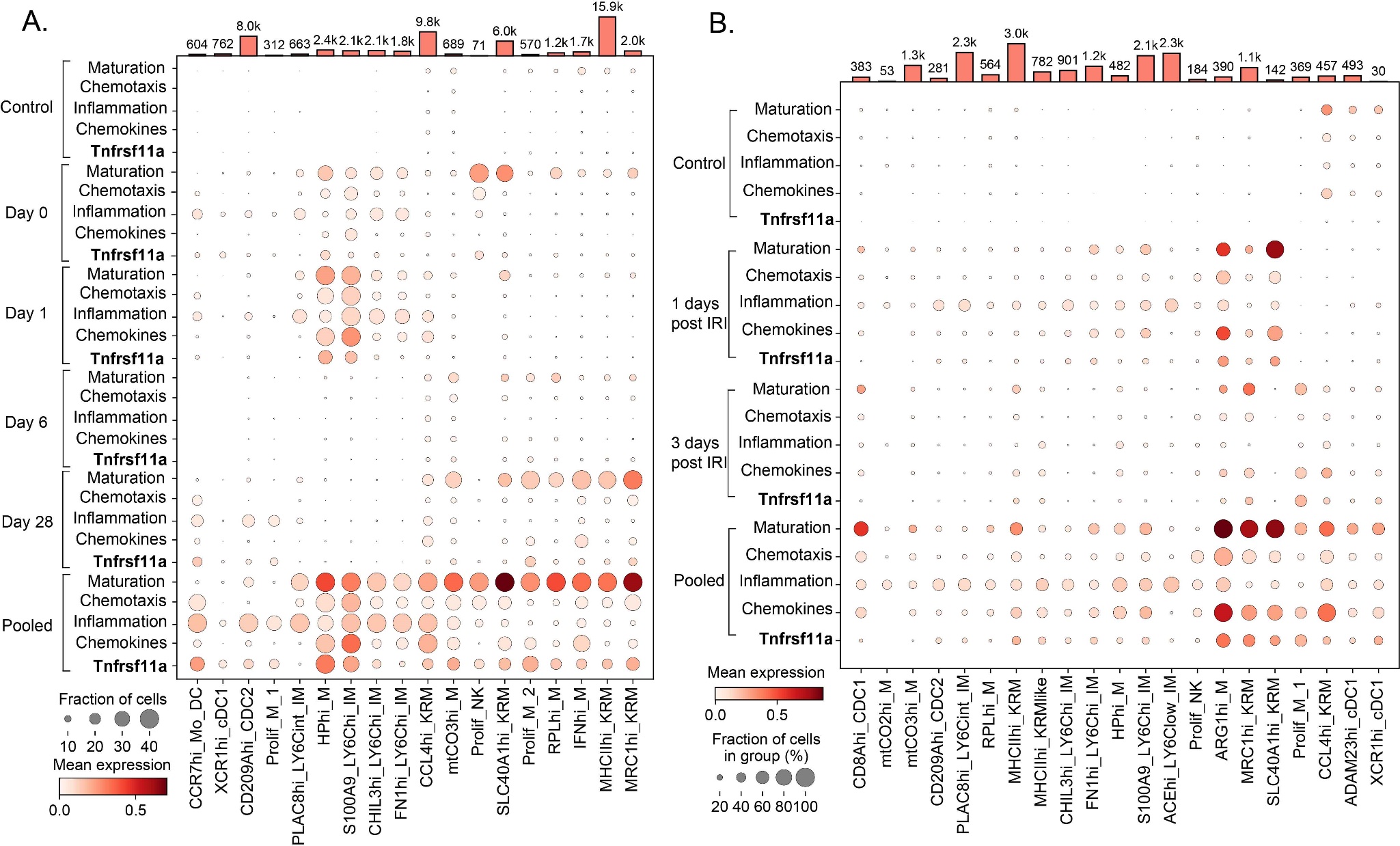


Supplementary Figure 5. Dot plot for gene sets enrichment and Tnfrsf11a expression in the kidney mononuclear cell after IRI.

Adopted from (A) GSE174324 and (B) GSE200115.The dot size represents the cells’ proportion in the group and the color represent the mean expression. The bars plot on the top of the plot are the numbers of the cell in group. IM, infiltrating monocytes; DC, dendritic cells; M, Monocyte; KRM, kidney-resident Mφ; Mo, Mφ; NK, natural killer cell.


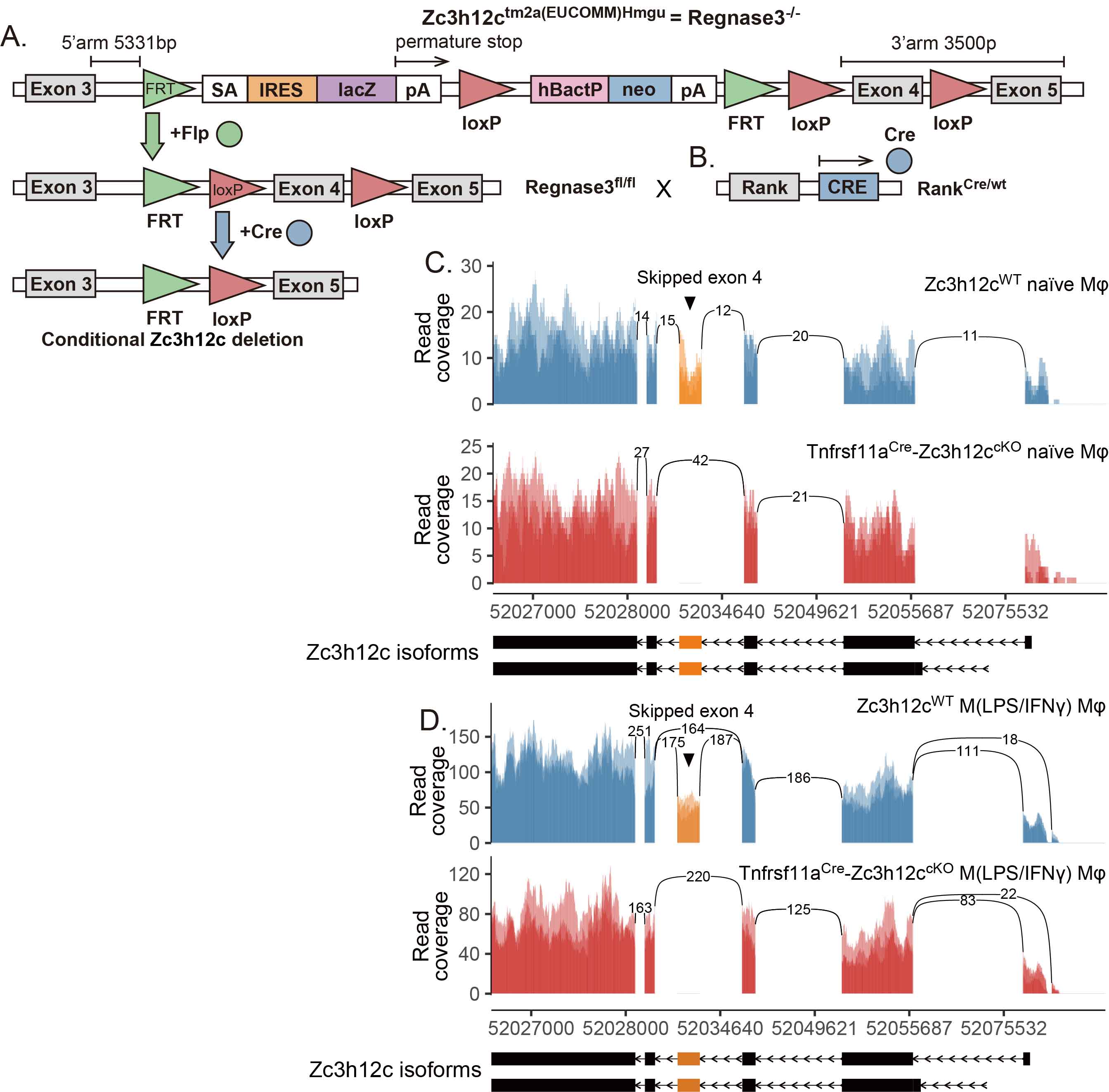


Supplementary Figure 6. Schematic representation of the targeting strategy for Zc3h12c conditional knockout mice.

(A) Targeting vector for Zc3h12c^tm2a (EUCOMM)Hmgu^ mice. Breeding strategies with promotor driven Flippase (FLP) recombinases and to obtain mice with a floxed Zc3h12c (Zc3h12c) allele and then mice with a conditional deletion of Exon 4 in the Zc3h12c gene locus upon the Cre recombinases present. (B) Cre recombinases insert into the Tnfrsf11a promotor. Sashimi plots for the Zc3h12c chr9:52026580-52080668 in Tnfrsf11a-Zc3h12c bone marrow derived (C) naïve and (D) M(LPS/IFNγ) Mφ. Tnfrsf11a-Zc3h12ccKO condition is shown in red plots, while the WT condition is depicted in blue. The X-axis shows the genomic locations, while the Y-axis indicates the transcription intensity. The plots show a "Sashimi-like" region, which is a heavily transcribed region of exon, and the blank intronic regions between exons. The line crossing exonic regions represent the reads of junction and the count number is indicated on it. The exonic structure of the Zc3h12c transcripts NM_001368810.1 and NM_001162921.2 is displayed below Sashimi plot, with the exonic region marked in yellow being the target that was inserted by flox and then deleted by recombinant Tnfrsf11a-Cre.


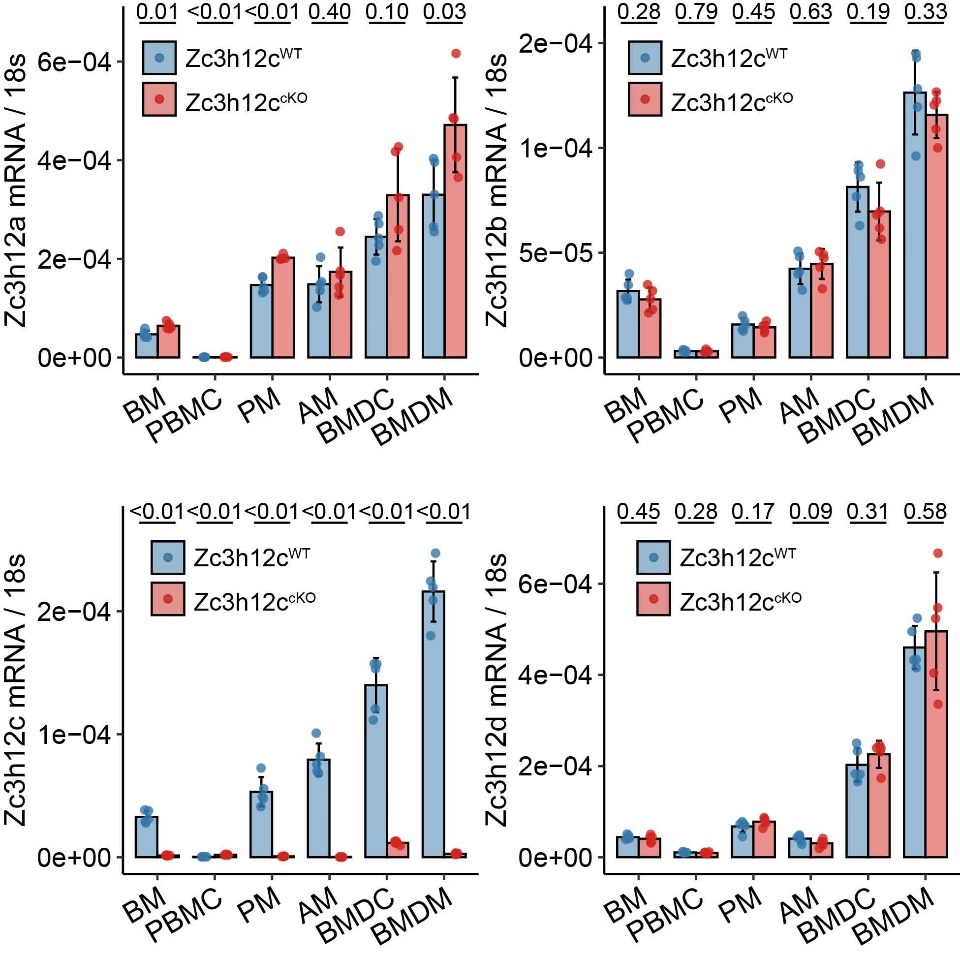


Supplementary Figure 7. Tnfrsf11a and (J-M) Zc3h12a-b mRNA levels in various phagocytes

The mRNA levels for Tnfrsf11a and Zc3h12a-b in bone marrow (BM), peripheral blood mononuclear cell (PBMC) alveolar Mφ (AM), BM derived dendritic cells (BMDC) and BM derived Mφ (BMDM) from Tnfrsf11a-Zc3h12c^cKO^ and wildtype (WT) mice. IM, infiltrating monocytes; Mo, Monocyte; KRM, kidney resident Mφ; Mo, Mφ; NK, natural killer cell; Tnfrsf11a, TNF Receptor Superfamily Member 11a; Zc3h12c, Zinc Finger CCCH-Type Containing 12C. All quantitative data are means ± SD (n = 5~7 biological replicates). Student t-test was employed for statistical comparison.


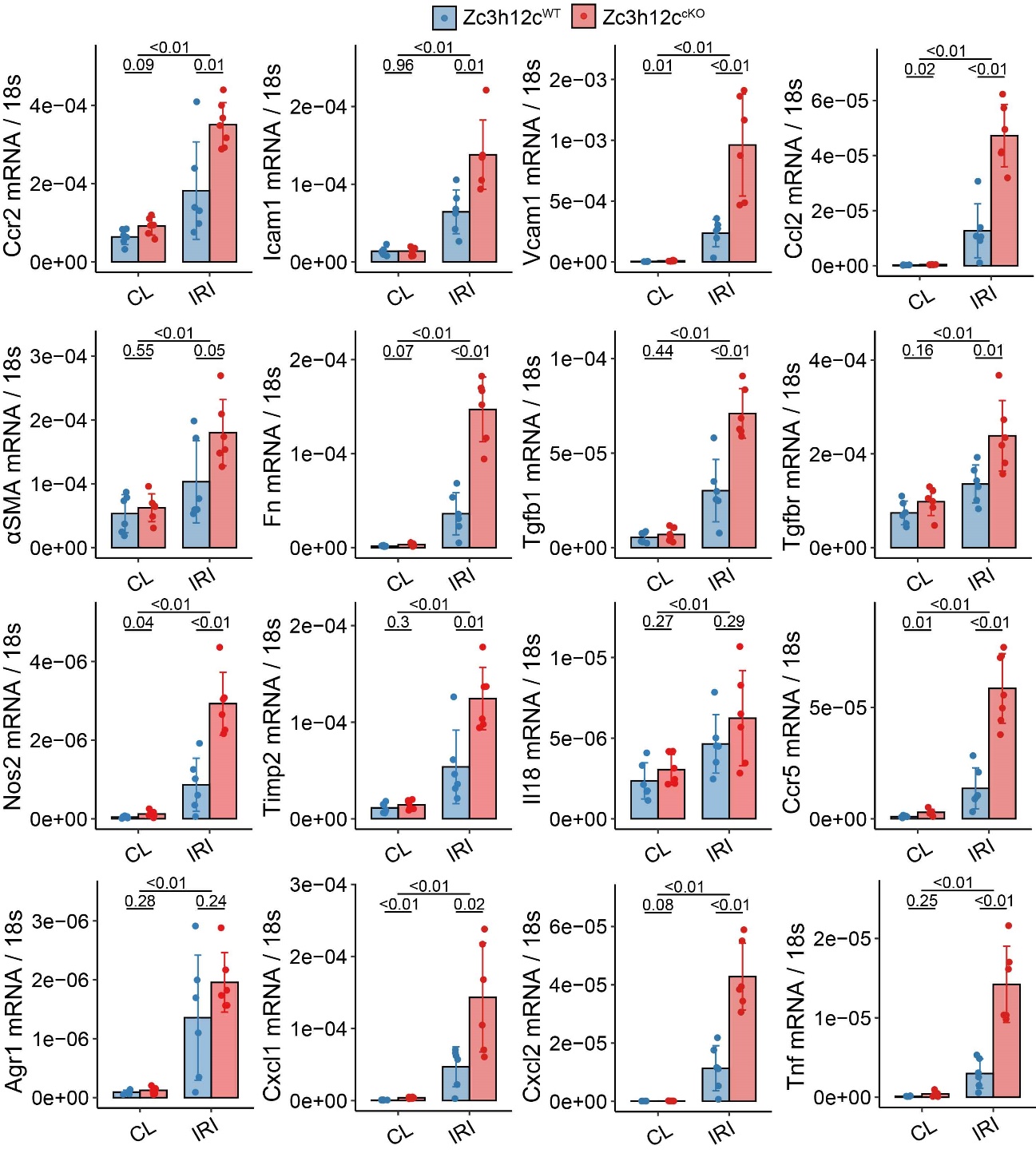


Supplementary Figure 8. The qPCR for selected genes in Tnfrsf11a-Zc3h12ccKO 21 days after 20 mins IRI.

The contralateral (CL) and ischemia-reperfusion injury (IRI) kidneys were harvested at day 21 after IRI. All quantitative data are means ± SD (n = 5~7 biological replicates). Two-way ANOVA or t-test was employed for the statistical examination.


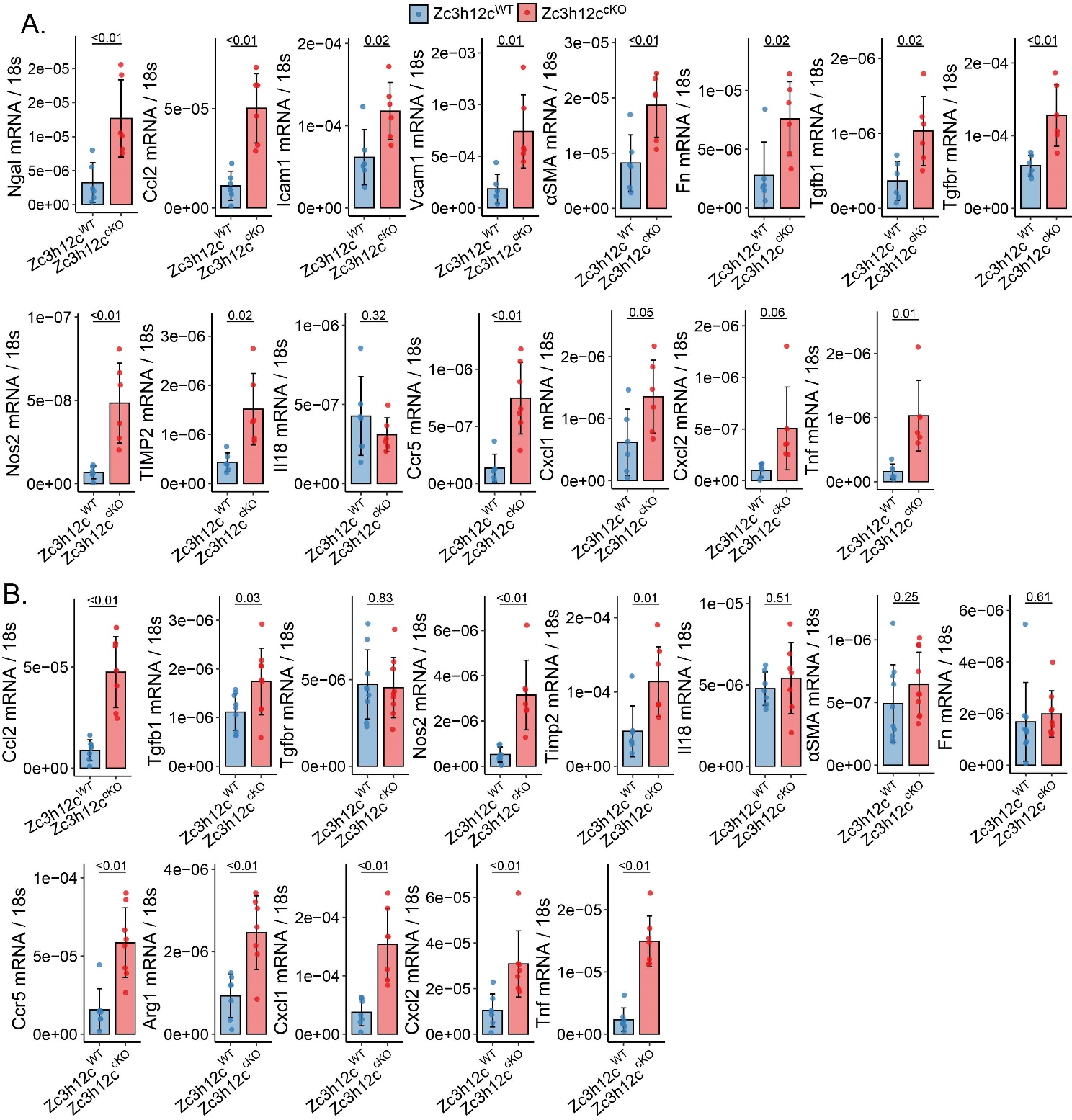


Supplementary Figure 9. The (A) Sirius Red staining and qPCR for kidney 21 days after (B) 16 mins IRI w/o NX and (C) CaOx model.

The kidneys were harvest at 21 days after IRI and 8 days after changing the CaOx-rich diet. All quantitative data are means ± SD (n = 5~7 biological replicates). Student t-test was employed for statistical comparison.


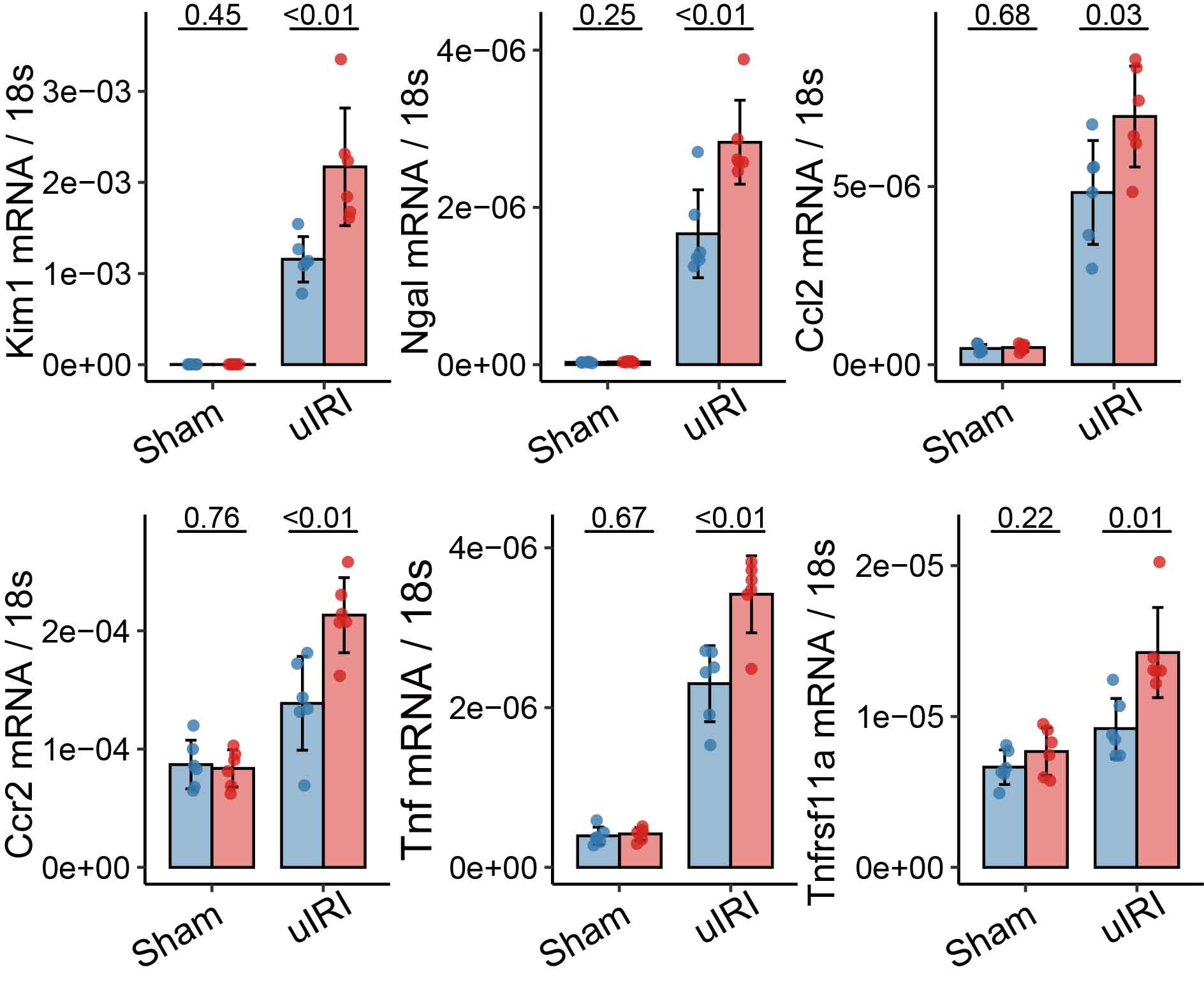


Supplementary Figure 10. The qPCR for Kim1, Ngal, Ccl2, Ccr2, Tnf and Tnfrsf11a in kidneys harvested on day 1 after uIRI.

The sham and ischemia-reperfusion injury (uIRI) kidneys were harvested at day 1 after IRI. All quantitative data are means ± SD (n = 5~7 biological replicates). Student t-test was employed for statistical comparison.


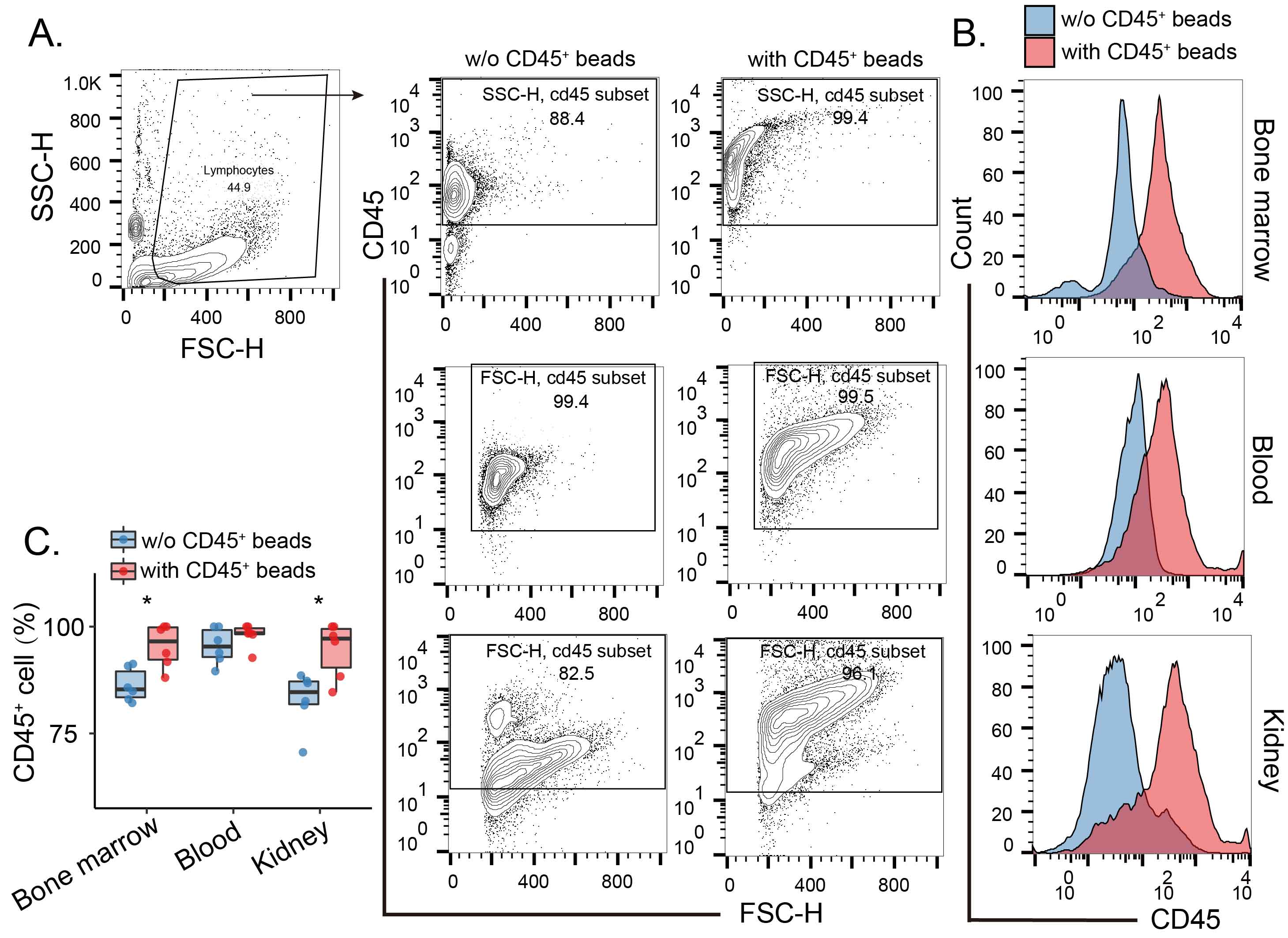


Supplementary Figure 11. CD45 magnetic beads sorted the immune cell from the blood, bone marrow, and kidney.

Gating strategy of CD45^+^ cell and the histogram for the CD45 in (A) blood, (B) bone marrow, and (C) kidney with or without CD45 magnetic beads. All quantitative data are means ± SD. T-test was employed for the statistical examination. *P-value lower than 0.05. All quantitative data are means ± SD (n = 5~7 biological replicates). Student t-test was employed for statistical comparison.


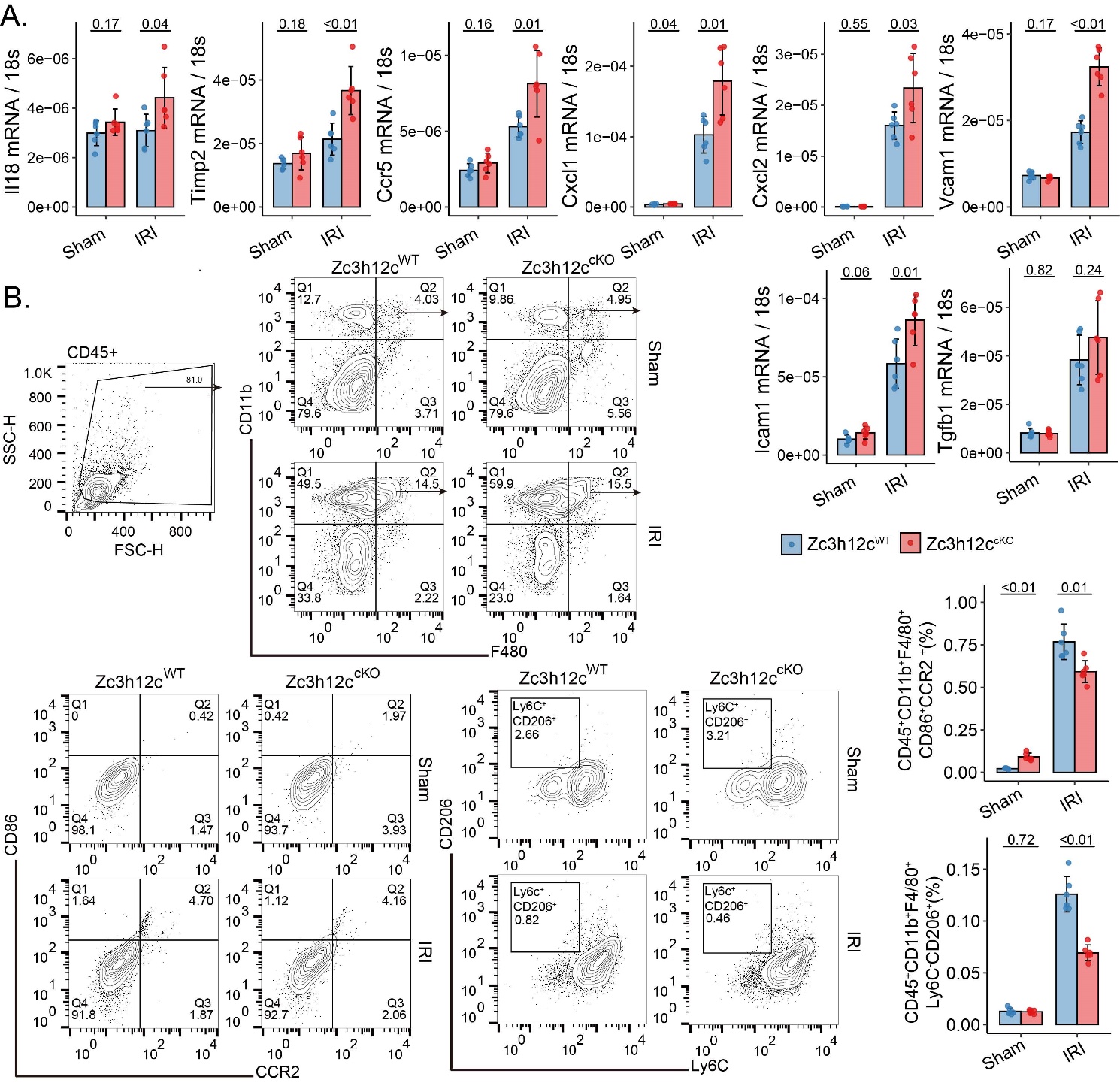


Supplementary Figure 12. The kidney pro-inflammatory and anti-inflammatory Mφ in of Tnfrsf11a-Zc3h12ccKO mice one day after IRI.

(A) qPCR for selected genes from IRI kidney (B) Gating strategy and quantitative for CD45^+^CD11b^+^F4/80^+^, CD45^+^CD11b^+^F4/80^+^CD86^+^CCR2^+^ and CD45^+^CD11b^+^F4/80^+^Ly6C^-^CD206^+^ cells in kidney one day post IRI by flow cytometry. All quantitative data are means ± SD (n = 5~7 biological replicates). Student t-test was employed for statistical comparison.


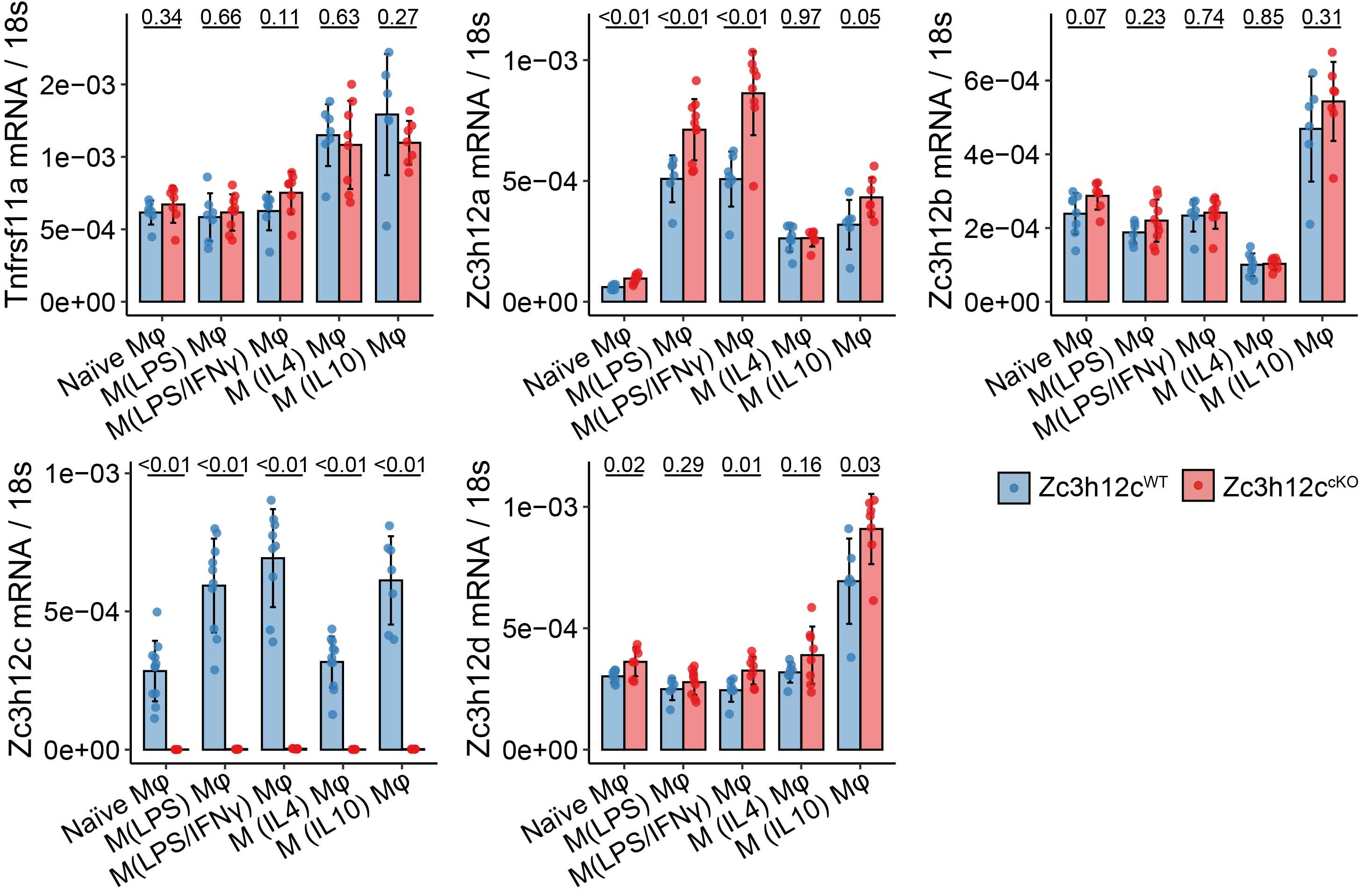


Supplementary Figure 13. Tnfrsf11a and Zc3h12 family member mRNA expression levels in pro-inflammatory or anti-inflammatory polarized Mφ in vitro.

The mRNA for Tnfrsf11a and Zc3h12a-d in naïve and LPS-, LPS/IFNγ-induced pro-inflammatory and IL4/IL10-induced anti-inflammatory Mφ. All quantitative data are means ± SD (n = 6~9 biological replicates). Student t-test was employed for statistical comparison.


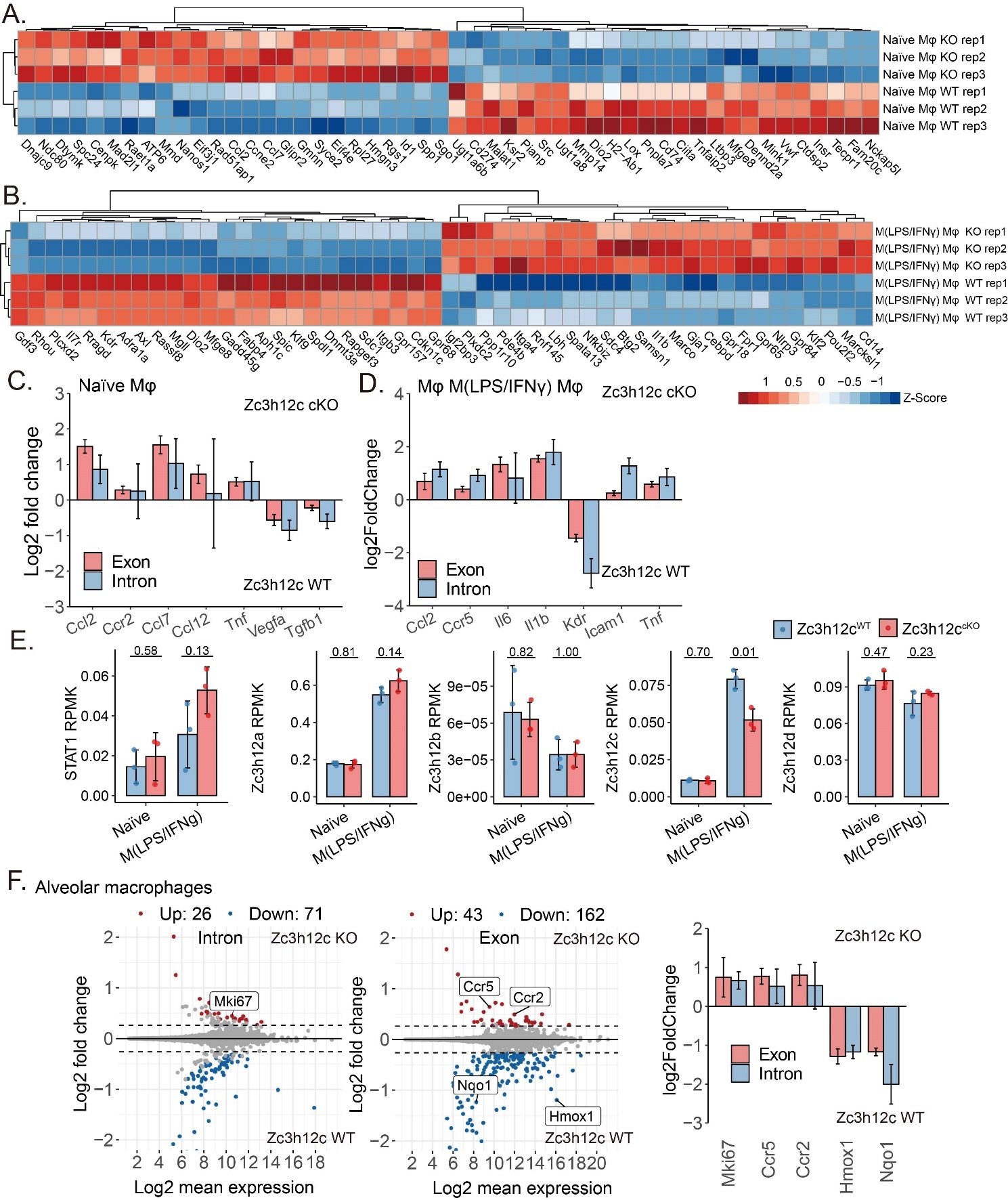


Supplementary Figure 14. Bulk RNA-seq on Zc3h12c knockout and wild type naïve pro-inflammatory bone-marrow-derived Mφ and alveolar Mφ.

Heat map of biological replicates of top differentially expressed genes in Tnfrsf11a-Zc3h12ccKO and WT (A) naïve and (B) pro-inflammatory Mφ. A heat map and dendrogram were used to display the differentially expressed genes’ z-scores of normalized counts. Selected gene expression in exon and intron at two conditions for (C) naïve and (D) M(LPS/IFNγ) Mφ. (E) Zc3h12 family and STAT1 mRNA level. All quantitative data are means ± SD (n = 3 biological replicates). T-test was employed for the statistical examination. (F) MA plot and selected gene expression for intron and exon in Zc3h12c KO alveolar Mφ.


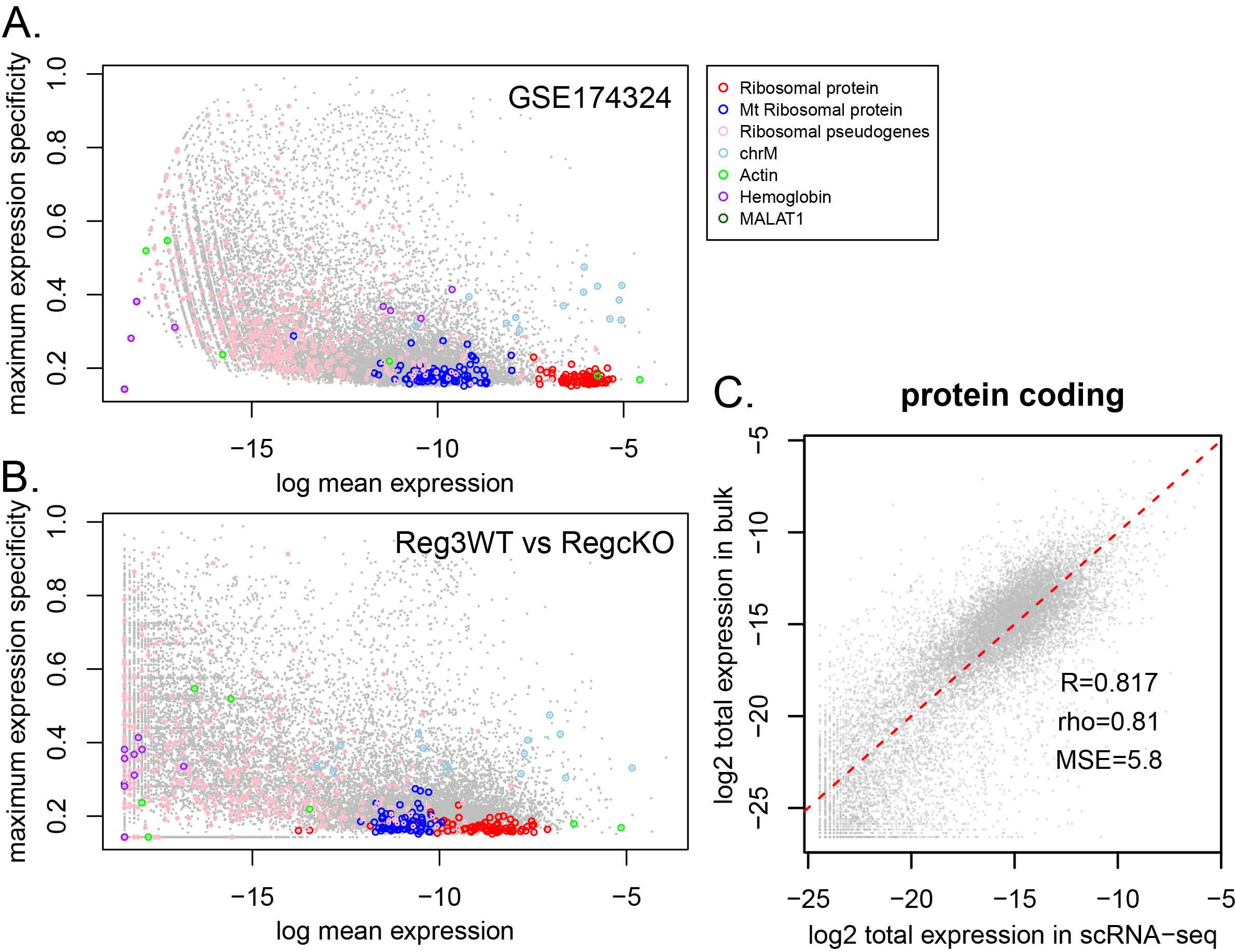


Supplementary Figure 15. Visualization on outlier genes and concordance plot for BayesPrism.

Visualization of the distribution of outlier genes for (A) scRNA-seq and (B) bulk RNA-seq. Two plots show the log of normalized mean expression (x-axis) and the maximum specificity (y-axis) of each gene, and if each gene belongs to a potential outlier category. Here, we just keep the protein coding genes for deconvolution. (C) concordance of gene expression between scRNA-seq and bulk RNA-seq.


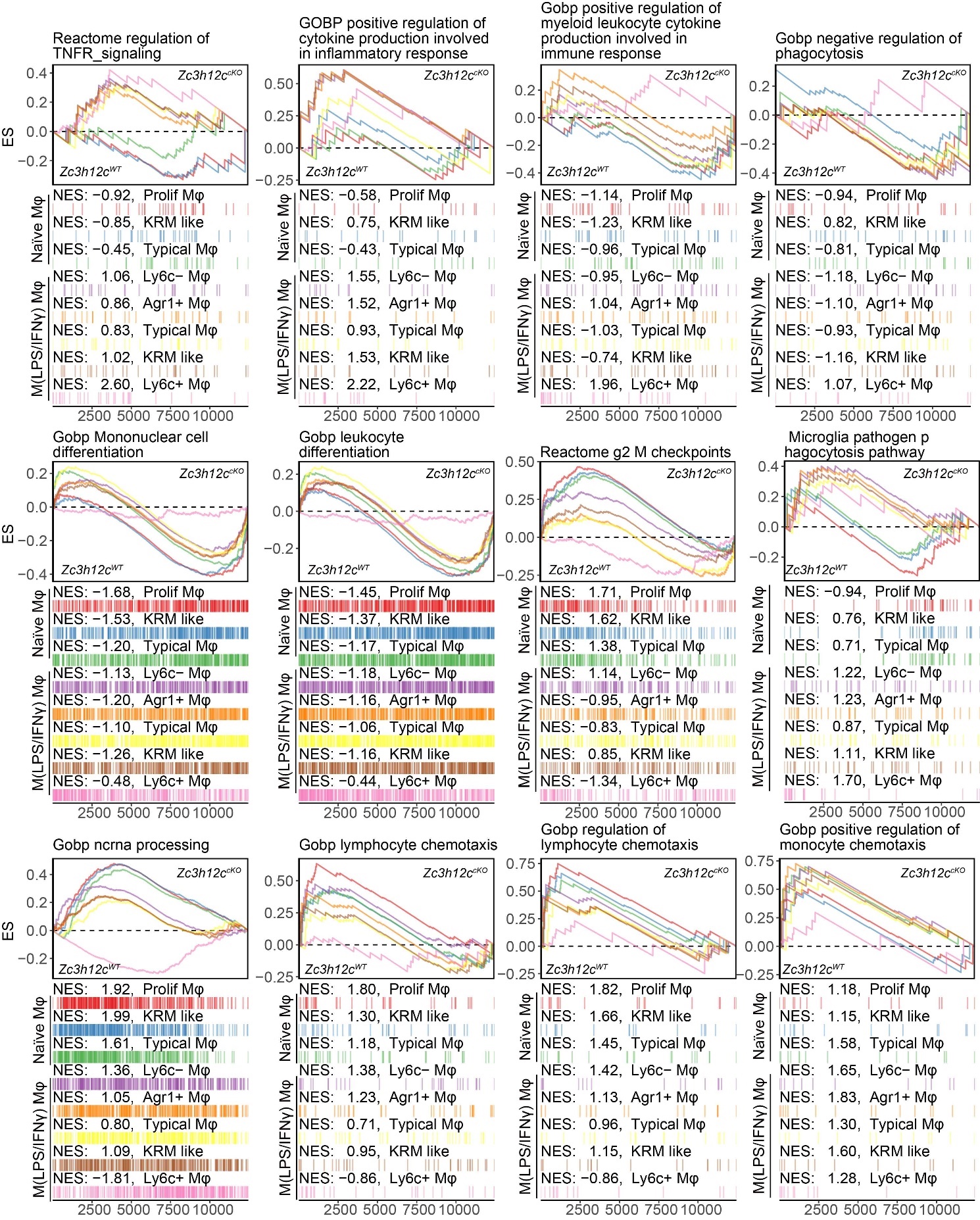


Supplementary Figure 16. Selected enrichment plots from the GSEA analysis.

The GSEA analysis based on the gene enrichment profiles on Tnfrsf11a-Zc3h12ccKO compared with WT refer to BayesPrism inferred Mφ subtype. The top portion of the plot shows the running enrichment score (ES). The bottom portion of the plot shows where the members of the gene set appear in the Tnfrsf11aed list of genes, with the normalized ES (NES).


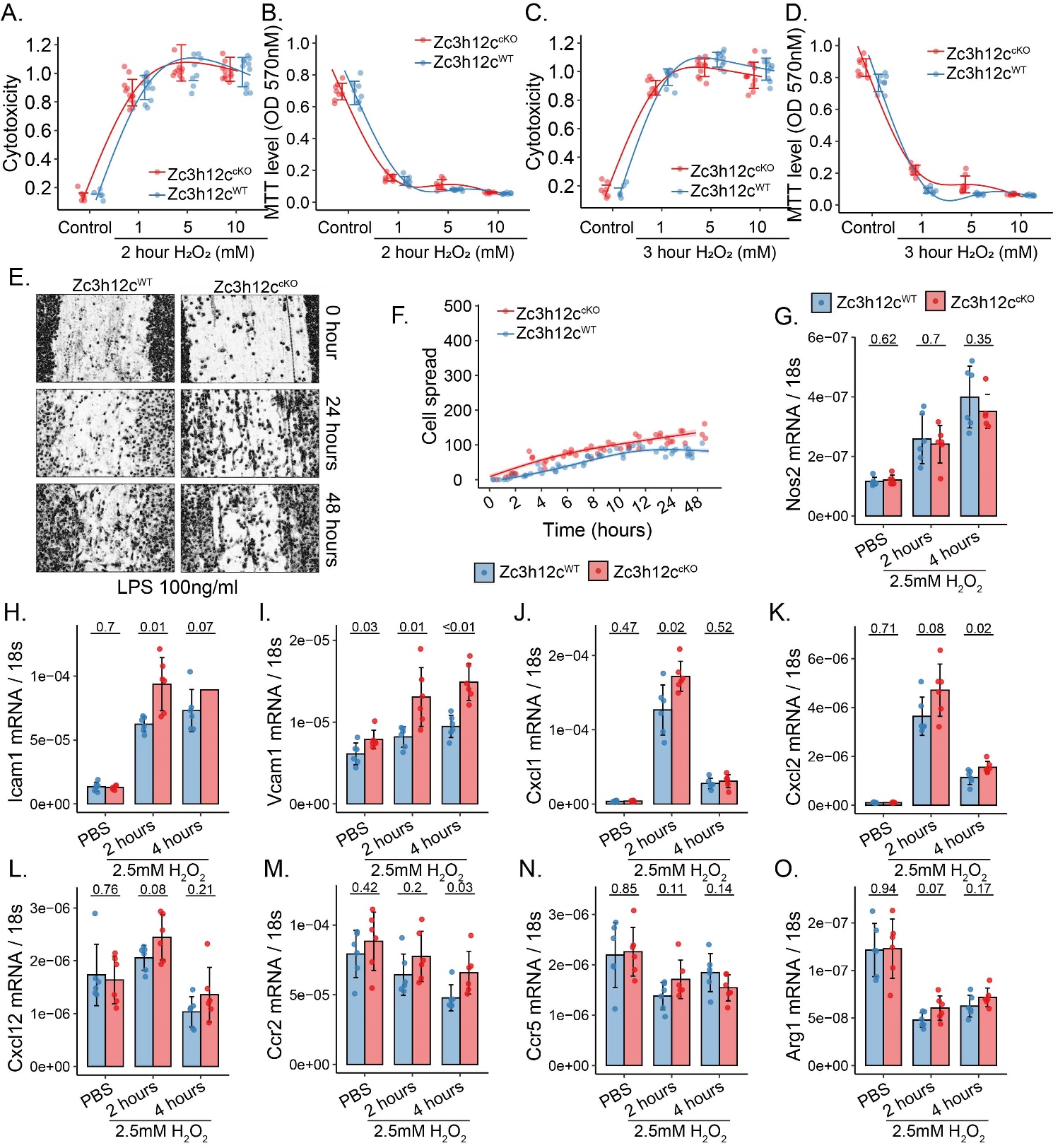


Supplementary Figure 17. Zc3h12c-deficiency modulates migration in Mφ.

(A) Cytotoxicity (calculated by the LDH OD450) and (B-D) metabolic activity in bone-marrow-derived Mφ (BMDM) from Tnfrsf11a-Zc3h12c^cKO^ and wildtype (WT) mice after stimulated with 1, 5 and 10 mM H_2_O_2_ for 2 and 3 hours. (E) Scratch-induced migration of BMDM from Tnfrsf11a-Zc3h12c^cKO^ and WT mice after H_2_O_2_ incubation. Images were taken at the 0, 24 and 48 hours after scratch in (F) after exposure to 100 ng/ml LPS for 24 hours. Cell spread represents the cell number that migrate into the gap. (G-O) The qPCR for the selected genes in BMDM, which were incubated with 2.5 mM H_2_O_2_ for 2 and 4 hours. All quantitative data are means ± SD (n = 6~8 biological replicates). T-test was employed for the statistical examination.


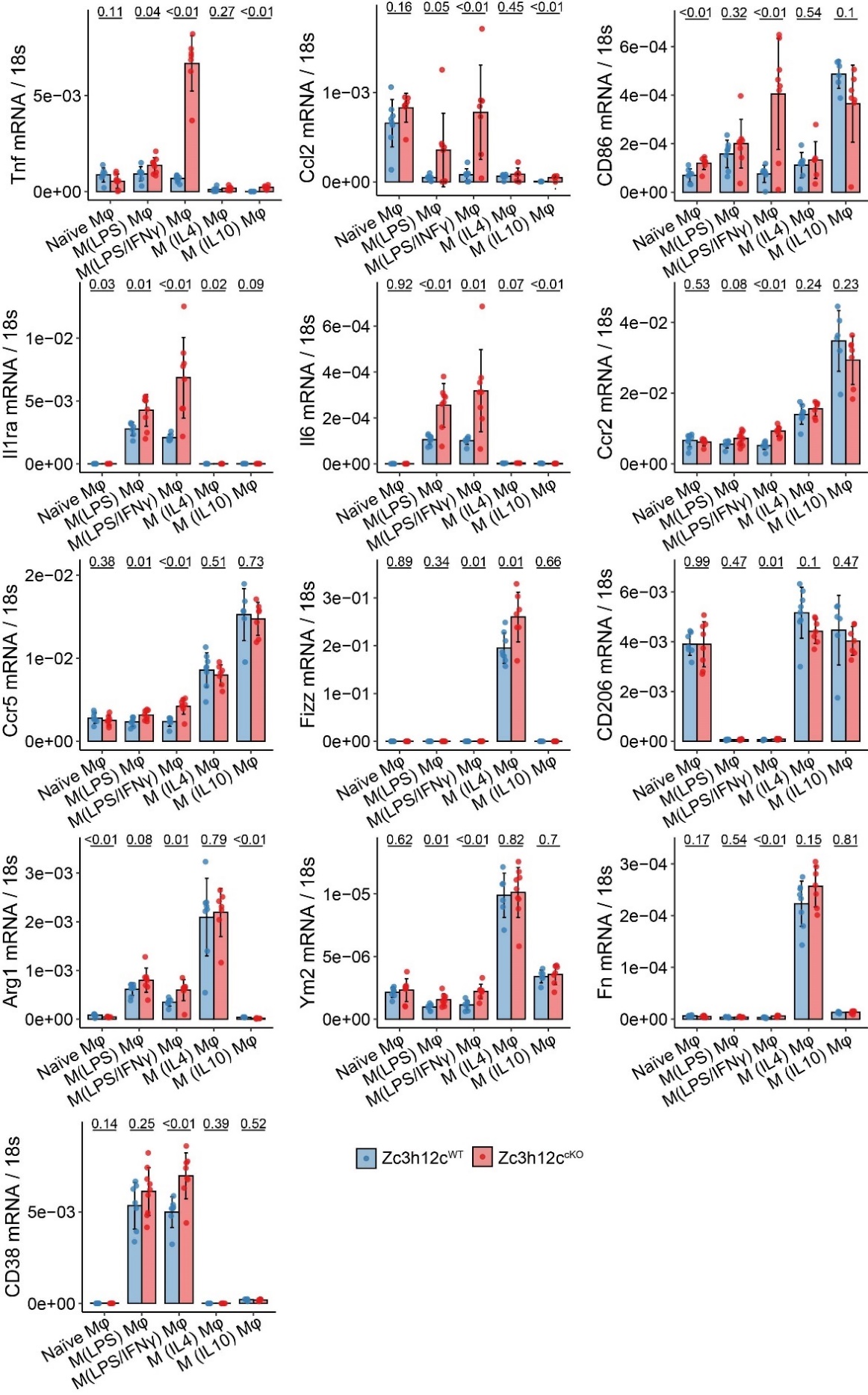


Supplementary Figure 18. The mRNA level for genes in pro-inflammatory and anti-inflammatory polarized Mφ.

The mRNA for Tnfrsf11a and Regnase 1 to 4 in unstimulated (naïve) and LPS, LPS/IFNγ induced pro-inflammatory and IL4/IL10-induced anti-inflammatory Mφ in wild-type (WT) and Tnfrsf11a-Cre-driven Zc3h12c conditional knockout (cKD) bone marrow-derived macrophages. All quantitative data are means ± SD (n = 6~9 biological replicates). T-test was employed for the statistical examination.


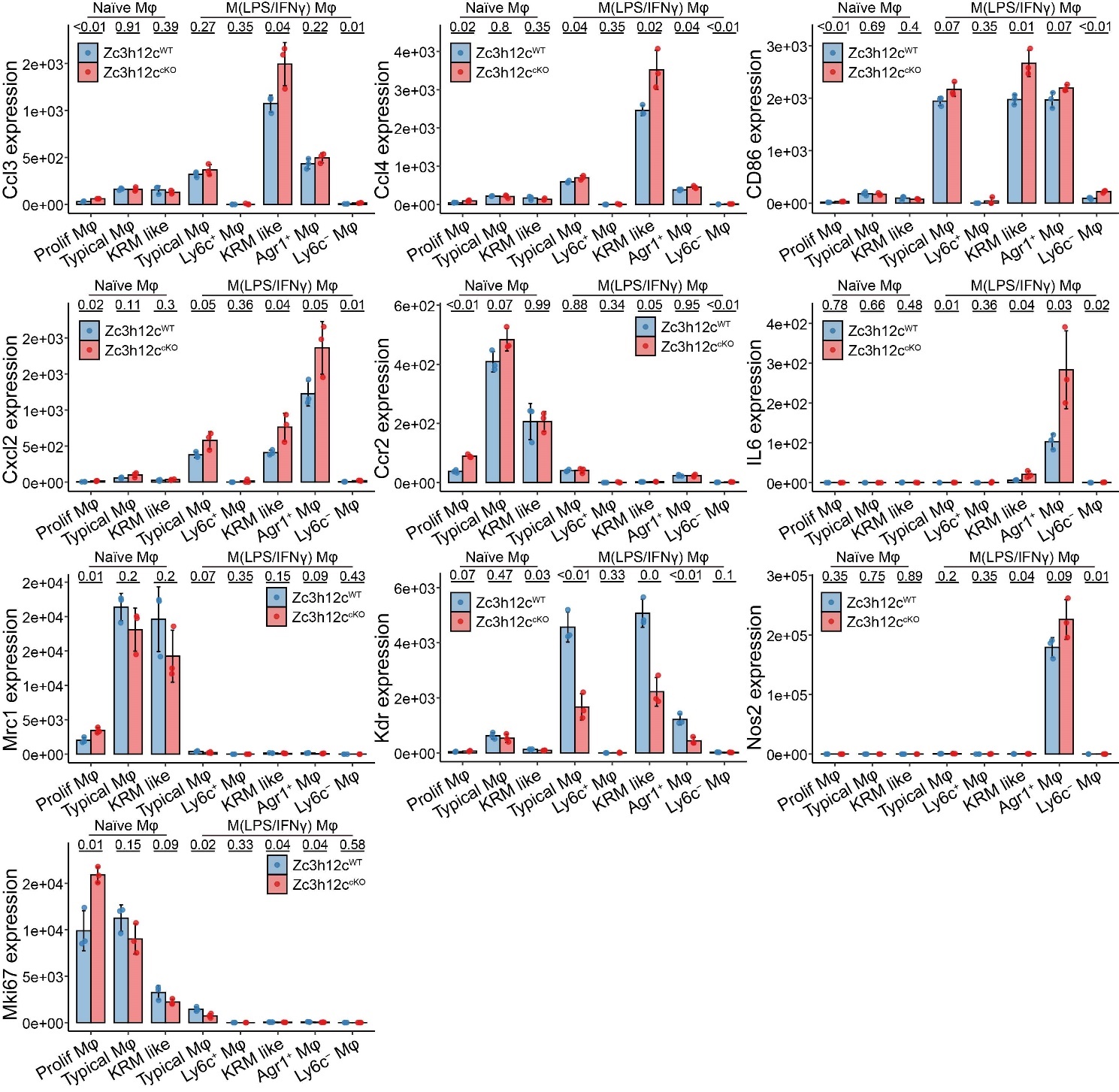


Supplementary Figure 19. Selected genes expression on BayesPrism inferred joint cell type fraction in Tnfrsf11a-Zc3h12c^cKD^ naïve and pro-inflammatory Mφ.

(A) Expression of selected genes across macrophage subpopulations in wild-type (WT) and Tnfrsf11a-Cre-driven Zc3h12c conditional knockout (cKD) bone marrow-derived macrophages. Cells were either unstimulated (naïve) or polarized to a pro-inflammatory state with LPS/IFNγ. Data represent BayesPrism-inferred cell-type-specific expression from bulk RNA-seq deconvolution using a murine kidney scRNA-seq reference (GSE174324). All quantitative data are means ± SD (n = 3 biological replicates). T-test was employed for the statistical examination.


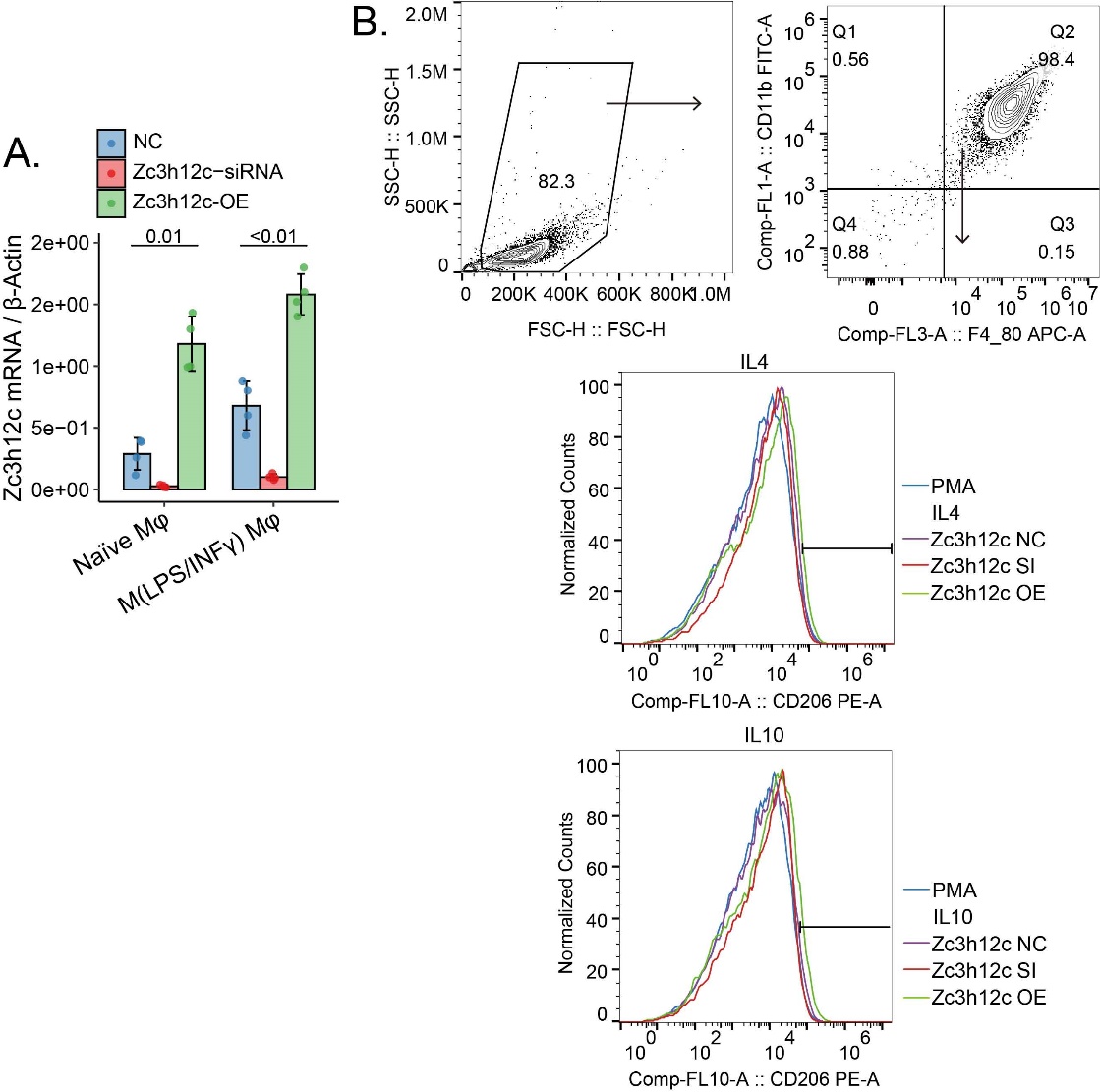


Supplementary Figure 20. Zc3h12c Modulates anti-inflammatory macrophage polarization marker CD206 expression in PMA-differentiated THP-1 macrophages.

(A) Zc3h12c mRNA levels in PMA-differentiated THP-1 macrophages transfected with control siRNA (NC), Zc3h12c-siRNA (siRNA knockdown), Zc3h12c-OE (overexpression plasmid). Data normalized to β-Actin and shown as mean ± SD (n = 3 biological replicates). (B) Flow cytometry analysis of macrophage activation: Top panel: Flow cytometry gating strategy. Bottom panel: Histogram showing CD206 (PE-A) expression in PMA-differentiated THP-1 macrophages polarized towards M2 phenotype with 10 ng/ml IL-4 or IL10 for 24 hours. Cells were transfected with control siRNA (Zc3h12c NC), Zc3h12c siRNA (Zc3h12c SI), or Zc3h12c overexpression vector (Zc3h12c OE), or treated with PMA alone (PMA) or IL-4 alone (IL4) as controls

Supplementary Data 1. Differential Expression Analysis of Naive BMDMs Zc3h12c cKO vs. WT

Supplementary Data 2. Differential Expression Analysis of LPS+IFNγ BMDMs Zc3h12c cKO vs. WT

Supplementary Data 3. Analysis of Primary Zc3h12c Motif Targets in the hg38 Genome

Supplementary Data 4. Analysis of secondary Zc3h12c Motif Targets in the hg38 Genome

Supplementary Data 5. Analysis of Primary Zc3h12c Motif Targets in the mm10 Genome

Supplementary Data 6. Analysis of secondary Zc3h12c Motif Targets in the mm10 Genome
